# Single-cell mapping of human plasma cell fate reveals a commitment checkpoint and Cav-1β expression associated with mTORC1 machinery

**DOI:** 10.1016/j.isci.2026.117865

**Published:** 2026-10-13

**Authors:** Gersende Caron, Fabienne Desmots, Mélanie Chabaud, Maud Lemarié, Vincent Olivier, Fabrice Chatonnet, Céline Nonn, Cassandra Jadeau, Simon Léonard, Céline Monvoisin, Kathleen Santamaria, Cédric M. Blouin, Noudjoud Attaf, Tarek Gharsalli, Pierre Milpied, Christophe Lamaze, Céline Pangault, Marion Haas, Thierry Fest

**Affiliations:** 1 Université de Rennes 1, INSERM, Établissement Français du Sang de Bretagne, UMR_S1236, 35043 Rennes, France; 2 Laboratoire d’hématologie, Pôle de Biologie, Centre Hospitalier Universitaire, 35033 Rennes, France; 3 Institut Curie – Centre de Recherche, PSL Research University, Membrane Mechanics and Dynamics of Intracellular Signaling Laboratory, Paris, France; 4 Institut National de la Santé et de la Recherche Médicale (INSERM), U1339 Paris, France; 5 Centre National de la Recherche Scientifique (CNRS), UMR 3666, Paris, France; 6 Aix Marseille University, CNRS, Inserm, CIML, Marseille, France

**Keywords:** human B cell differentiation, plasma cell differentiation, single-cell resolution mapping human plasma cell differentiation, XBP1-driven induction of CAV1, non-caveolar Cav-1β, endoplasmic reticulum, secretory machinery, and nutrient-sensing complexes, V-ATPase-RAGulator pathway

## Abstract

Human B cell differentiation into antibody-secreting plasma cells (PCs) requires coordinated transcriptional and organelle remodeling. Using single-cell transcriptomics in an *in vitro* human B cell differentiation system, we identified a Day-8 commitment checkpoint separating PC, memory B cell, and germinal center-like trajectories and defined an XBP1-associated transcriptional program linked to organelle remodeling. Within this program, *CAV1* was induced during PC commitment and encoded a non-caveolar Cav-1β isoform expressed in plasmablasts and PCs. Proteomic analyses showed that Cav-1β associates with endoplasmic reticulum, secretory, and nutrient-sensing proteins, including components of the V-ATPase-RAGulator-RAG complex. *CAV1* silencing had modest effects on PC differentiation but did not alter acute mTORC1 activation or antibody secretion.

These findings place Cav-1β within the membrane and organelle remodeling program of human PCs and highlight how intracellular architecture may be coupled to metabolic and secretory adaptation during plasma cell differentiation.

## Highlights

• Single-cell profiling identifies a commitment checkpoint in plasma cell differentiation
• XBP1 induces non-caveolar Caveolin-1β during plasma cell differentiation
• Caveolin-1β associates with nutrient-sensing and mTORC1 protein networks
• Caveolin-1β modestly influences plasma cell differentiation

## Graphical abstract

**Figure:**
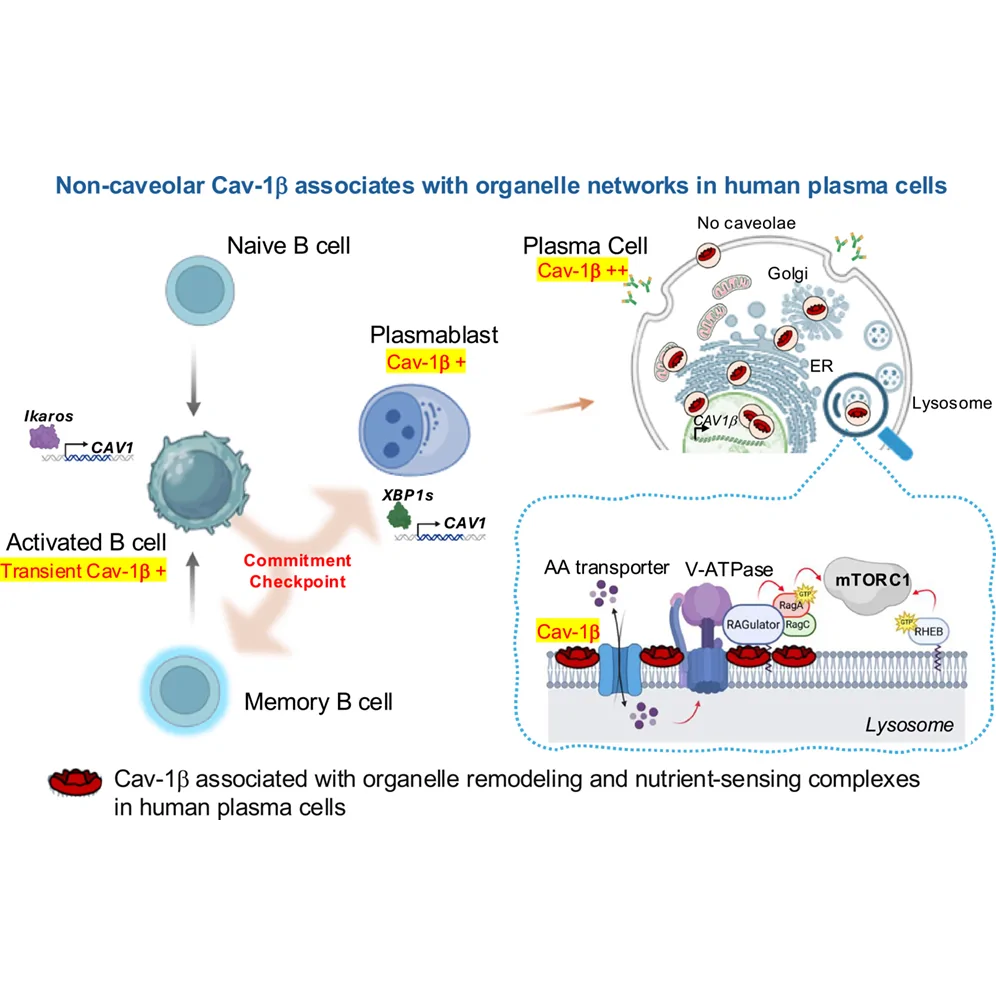

## Teaser

Cell biology; Functional aspects of cell biology; Integrative aspects of cell biology; Organizational aspects of cell biology

## Introduction

The differentiation of naive B cells into antibody-secreting plasma cells (PCs) requires extensive transcriptional and structural remodeling. Whereas resting B cells display a large nucleus and limited cytoplasm, terminal PCs undergo massive expansion of the endoplasmic reticulum (ER) and Golgi apparatus to sustain high-rate immunoglobulin secretion, often for decades in long-lived PCs.^1^^,^^2^^,^^3^ This secretory transformation activates adaptive stress pathways, most prominently the unfolded protein response (UPR). Within this network, the IRE1–XBP1 axis is indispensable for establishing the secretory and metabolic architecture of PCs and for coordinating the large-scale membrane biogenesis that supports antibody production.^4^

High-affinity PCs typically emerge following antigen engagement and CD4^+^ T cell help within germinal centers (GCs). GC B cells dynamically transit between proliferative dark zone states and selection in the light zone, where signals from the B cell receptor (BCR), CD40, and T follicular helper cells determine cell fate.^5^ Positively selected cells may re-enter the GC cycle, differentiate into memory B cells (MBCs), or commit to the PC lineage. This decision is accompanied by transient *MYC* induction and activation of mTORC1 signaling, which together promote metabolic reprogramming, proliferation, and differentiation.^6^^,^^7^^,^^8^^,^^9^^,^^10^ Recent single-cell analyses have positioned GC B cells along a continuum of proliferative, activated, and effector-committed states, with Myc^+^/mTORC1 activation marking a key transition toward terminal differentiation.^5^

mTORC1 integrates activation cues with metabolic and secretory remodeling during PC differentiation. It precedes full UPR engagement and supports successive waves of ER expansion and antibody production.^11^^,^^12^^,^^13^ In both murine and human B cells, mTORC1 activity precedes full UPR engagement and supports antibody secretion.^14^^,^^15^ Notably, mTORC1 can partially compensate for the loss of classical UPR effectors, underscoring its importance in coordinating metabolic and secretory adaptations during PC differentiation^11^ and suggesting that nutrient-sensing pathways are tightly coupled to the structural remodeling of intracellular membrane systems.

Despite increasing resolution of transcriptional and metabolic programs governing PC differentiation, how intracellular membrane systems are structurally coordinated during this transition remains incompletely understood. To address this question in a human context, we established an *in vitro* culture system that drives differentiation of tonsillar B cells into antibody-secreting cells (ASCs) over ten days, supported by stromal cells and defined cytokine signals.^16^^,^^17^ Single-cell transcriptomic analysis of this system revealed a reproducible bifurcation in B cell fate, separating cells committed to terminal PC differentiation from those retaining a B cell identity as MBCs or GC-like cells.

Integrative network analysis identified a PC-associated transcriptional module enriched for XBP1 targets and organelle biogenesis genes. Within this module, we identified *CAV1*, encoding Caveolin-1 (Cav-1), a membrane-associated scaffolding protein classically implicated in caveolae formation in mechanically stressed cell types such as endothelial cells and adipocytes.^18^ Beyond caveolae, Cav-1 has been detected in non-caveolar intracellular compartments, including the Golgi apparatus, ER, and endolysosomal membranes, and has been implicated in diverse processes such as membrane trafficking, autophagy, and cancer biology^19^^,^^20^ as well as in the spatial organization of signaling and metabolic complexes on intracellular membranes. Notably, *Cav1* expression is absent from murine PCs, suggesting potential species-specific mechanisms in plasma cell architectural organization.

Here, we combine single-cell transcriptomics, functional perturbation, and proteomic mapping to define Cav-1 expression and function during human PC differentiation. We identify a biphasic regulation of *CAV1* and demonstrate that human plasmablasts and PCs selectively express a non-caveolar Cav-1β isoform induced downstream of XBP1. Although Cav-1β only modestly influences differentiation dynamics, proteomic analysis reveals its extensive association with ER, lysosomal, and nutrient-sensing machinery, including components of the V-ATPase-RAGulator-RAG complex. These findings uncover a previously unrecognized membrane-associated scaffold integrated into the XBP1-driven remodeling program of PCs and provide new insight into how metabolic and secretory architectures are coordinated during human PC differentiation.

## Results

### Single-cell profiling reveals bifurcating differentiation trajectories in human B cell cultures

We established a two-step stromal co-culture system that drives human tonsillar B cells toward ASC differentiation. In the first phase, B cells were activated on CD40L-expressing stromal cells (MS40L) in the presence of BAFF, IL-4, IL-21, and IL-2. Cells were then transferred onto MS5 stromal feeder cells with IL-6 to promote terminal differentiation (Figure 1A), confirmed by the generation of CD20^lo^CD38^hi^CD138^+^ PCs (Figure 1B).

**Figure 1:**
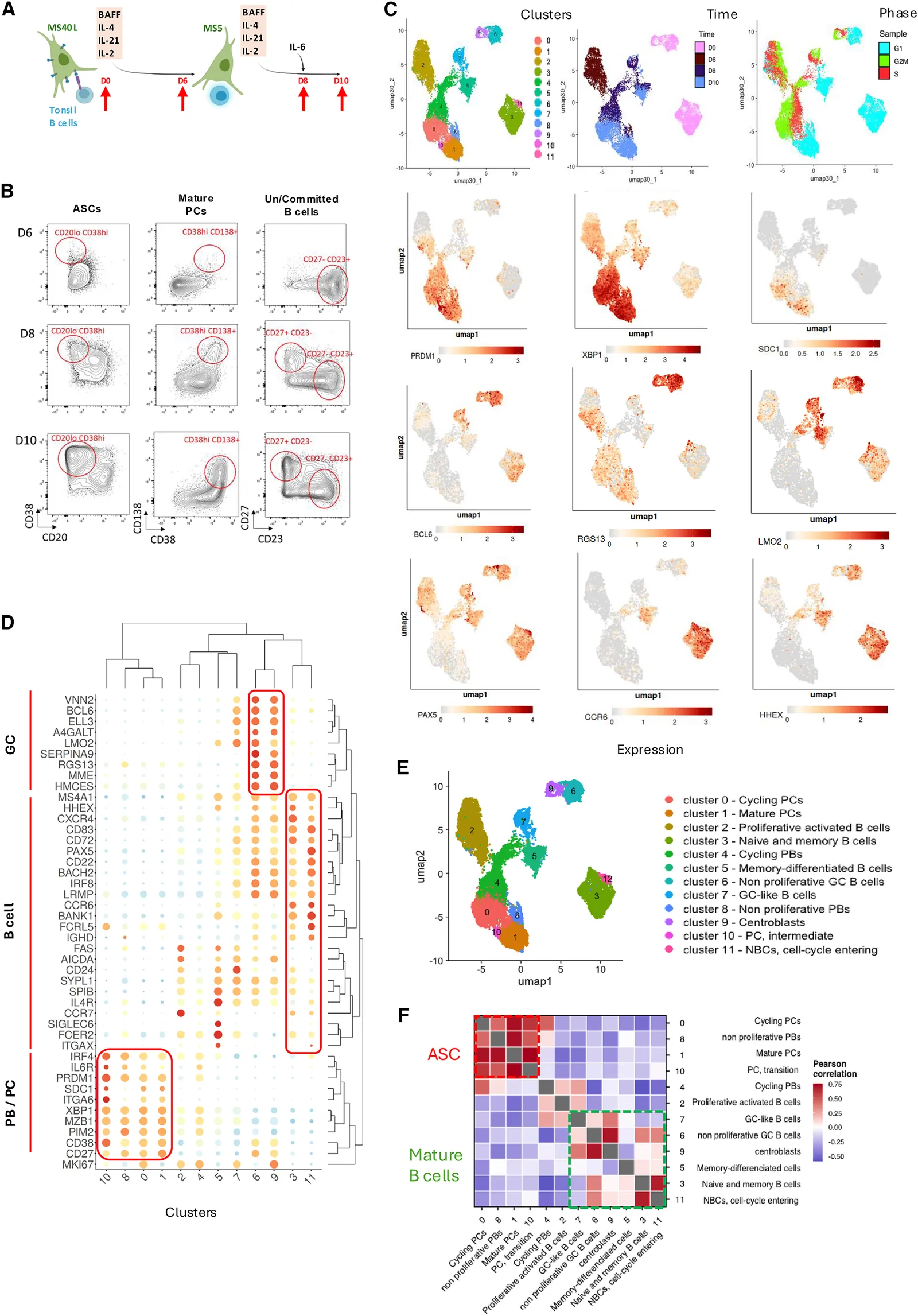
Single-cell transcriptomic analysis of *in vitro*-induced human tonsillar B cell differentiation (A) Schematic overview of the *in vitro* B cell differentiation model. Purified CD19^+^ B cells from three independent human tonsils were cocultured with MS-CD40L (MS40L) stromal cells and cytokines (IL-21, IL-4, BAFF, IL-2) for 6 days, followed by 4 additional days on MS-only stromal cells with IL-6 supplementation. Arrows indicate collection time points for single-cell RNA sequencing (scRNA-seq). (B) Flow cytometry analysis of *in vitro* B cell differentiation at days 6, 8, and 10. Four distinct populations were identified: (i) CD20lo CD38hi antibody-secreting cells (ASCs); (ii) CD38^hi^ CD138^+^ mature plasma cells (PCs); (iii) CD27^+^ CD23^−^ B cells committed to plasmablast fate; and (iv) CD27^−^ CD23^+^ post-activated uncommitted B cells. Data are representative of three independent experiments and donors. (C) UMAP representation of scRNA-seq data from *in vitro*-differentiated B cells. Cells are colored by Seurat cluster assignment (left), culture day (middle), and cell cycle phase (right). Expression of selected marker genes is projected in red, with color intensity indicating normalized expression per cell. (D) Bubble plot showing the expression of genes characteristic of B cells, germinal center (GC) cells, and plasmablast/plasma cell (PB/PC) populations across clusters. Dot size indicates the percentage of cells expressing each gene within a cluster, and color intensity reflects the average expression level. (E) Cluster annotation based on marker gene expression profiles. (F) Pearson’s correlation matrix showing the transcriptional relationships among clusters. Correlation coefficients were calculated from the average expression values of all genes in each cluster; warmer colors denote higher correlations with two main clusters named ASC in red and mature B cells in green.

To map transcriptional dynamics along this differentiation process, we performed scRNA-seq at day 0 (D0), D6, D8, and D10, profiling 17,220 cells from three donors. Unsupervised clustering identified 12 transcriptionally distinct populations (Figure 1C), with balanced donor representation across clusters, except for the D6 cluster that was underrepresented in a single donor (Figures S1A and S1B). Early clusters (D0–D6) corresponded to mature and GC B cells, D8 cells populated transitional states, and D10 cells aligned with ASCs. Cell-cycle analysis revealed strong proliferation at D6–D8 and cell-cycle exit in most D10 cells (Figure 1C).

Marker-based annotation delineated three principal cellular states: GC B cells (*BCL6, RGS13, LMO2*), mature B cells (*PAX5, CCR6, HHEX*), and ASCs (*PRDM1, XBP1, SDC1*), with transitional clusters bridging these states (Figures 1C and 1D). *AICDA* expression persisted from D0 through D8, consistent with ongoing activation and diversification processes (Figure S1C). GSEA further supported these annotations (Figure 1E and Table S1). Surface marker analysis segregated B cell-like and ASC-like populations (Figure S1D), and lineage similarity analysis revealed a bifurcation toward either ASC-associated clusters (0, 1, 8, and 10) or mature B cell-associated clusters (5 and 7) (Figure 1F).

Together, these data demonstrate that human B cell differentiation in this system follows bifurcating trajectories toward plasma cell or mature B cell fates, providing a framework for dissecting lineage-specific regulatory programs.

### Combinatorial cytokine and receptor signaling generates early heterogeneity in B cell fate commitment

D0 clusters recapitulated the expected heterogeneity of tonsillar B cells (Figure S1B). Cluster 3 contained a mixture of naive and memory B cells, with approximately one-third expressing *CD27*^+^ or *FCLR4*^+^, downregulating *IGHD*, and expressing class-switched Ig isotypes such as *IGHA1* (Figure S2A). Pathway enrichment showed TNFα and interferon responses, consistent with the inflammatory tonsillar context (Table S1). Cluster 9 exhibited features of dark zone (DZ) centroblasts, including high *STMN1* expression, whereas cluster 6 corresponded to light zone (LZ) centrocytes expressing *LMO2* and included *STMN1*^+^ G1 cells (Figure 2A).

**Figure 2:**
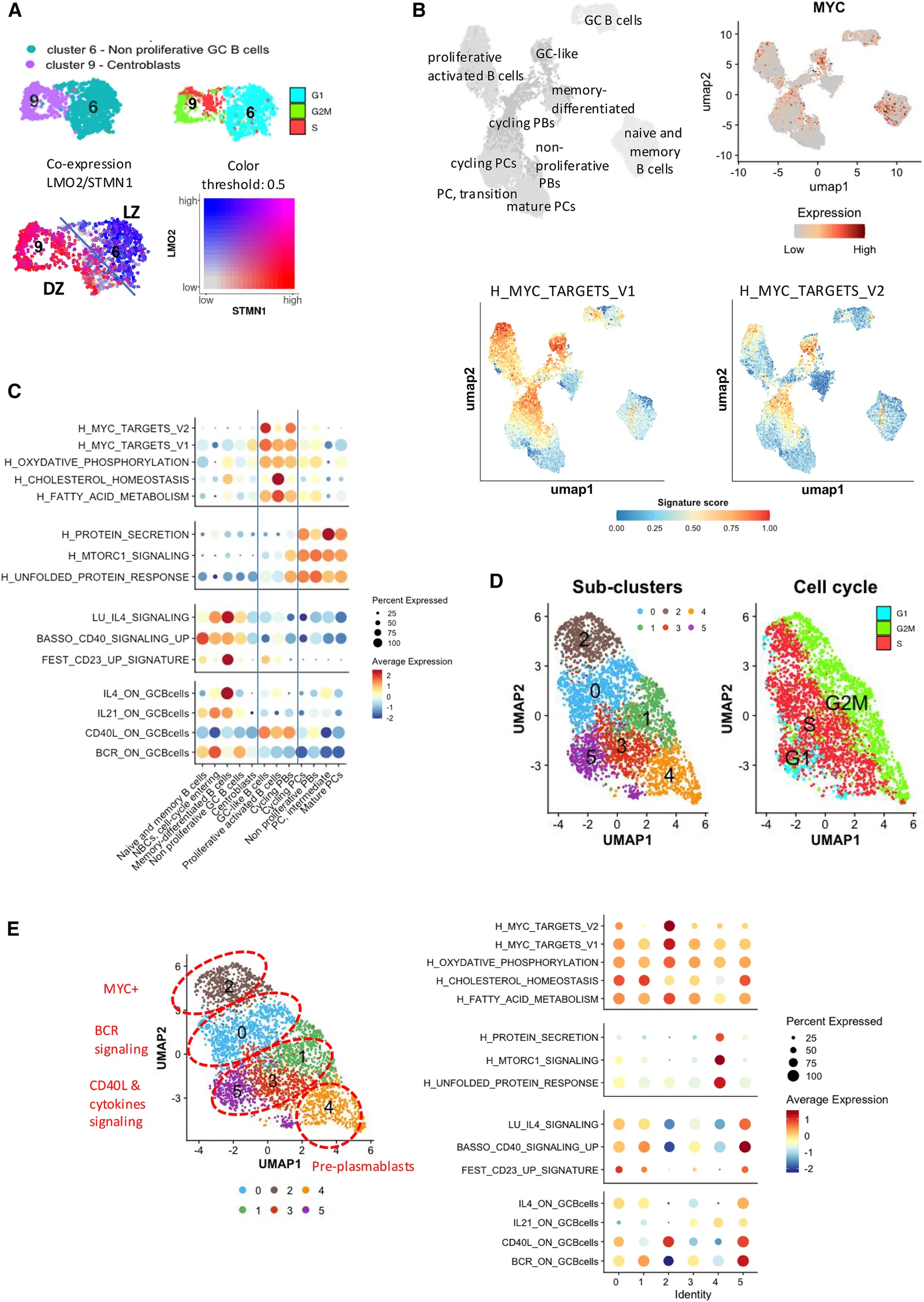
Signal-specific gene signatures reveal early heterogeneity in B cell activation and differentiation (A) Distribution of cell cycle phases and Germinal Center (GC) B cell states. Analysis of dark zone (DZ) and light zone (LZ) states at day 0 within clusters 6 and 9. These zones were delimited by the expression of marker genes *LMO2* and *STMN1,* as highlighted on the UMAP. The relative color scale (right) indicates higher expression of *LMO2* (blue) in the LZ and higher expression of *STMN1* (red) in the DZ. (B) UMAP plots show *MYC* transcript expression during B cell differentiation and enrichment of MYC target gene signatures (H_MYC_TARGETS_V1 and H_MYC_TARGETS_V2 from MSigDB; H indicates Hallmark collection). (C) Bubble heatmap shows expression levels of indicated related gene signatures across clusters. (D) UMAP plot of proliferative activated B cells (cluster 2) colored by subcluster identity (left) and cell cycle phase (right). Cells with low-quality transcriptomes are shown in light gray and excluded from further analysis. (E) Left: functional annotation of activated B cell subclusters based on marker gene expression. Right: bubble heatmap shows the enrichment of representative gene signatures in each subcluster.

As expected, proliferative clusters at D6 (cluster 2) and D8 (clusters 4 and 7) were enriched for MYC targets and metabolic programs, whereas quiescent naive and MBC populations at D0 (cluster 3) expressed MYC without activation of canonical MYC-driven transcriptional programs. In contrast, ASC-associated clusters (0, 1, 4, 8 and 10) showed enrichment for UPR, secretory, and mTORC1 signaling pathways (Figures 2B and 2C).

To connect these transcriptional states to upstream cues, we generated stimulus-specific gene signatures by RNA-seq profiling of GC B cells stimulated *ex vivo* with IL-4, IL-21, CD40L, or BCR engagement in 500-cell mini-bulk cultures (see STAR Methods; Table S2). Projection of these signatures onto the scRNA-seq atlas revealed preferential enrichment of IL-21 and BCR responses in naive/MBC and LZ GC-like clusters (clusters 3, 5, 6, and 11), IL-4 signaling in MBCs (cluster 5), and CD40L signaling in proliferative D6–D8 populations (clusters 2 and 4), coinciding with MYC-driven metabolic reprogramming (Figures 2C and S2B). Although this CD40L signature differed from a previously reported CD40L response in naive/MBCs,^21^ likely reflecting differences in cell type and stimulation duration, both signatures showed strong enrichment in D6 cells, consistent with sustained CD40L exposure in the culture system.

Consistent with IL-4–driven maintenance of B cell identity, a CD23^+^ B cell signature associated with STAT6 signaling and inhibition of PC differentiation^17^ was selectively enriched in cluster 5, supporting its annotation as a memory-like population persisting at D10 (Figure 2B).

Despite forming a single proliferative cluster, D6 cells segregated into six transcriptionally distinct proliferative subsets (Figures 2D and S2C). Sub-cluster 2 was enriched for MYC and CD40L targets and expressed *PTTG1*, a regulator of proliferation and MYC activity (Figure 2E and Table S3).^22^^,^^23^ Sub-cluster 0 retained BCR- and CD23-associated programs and expressed *IGHD*, *PAX5*, and *NIBAN3*, encoding the BCR signaling regulator BCNP1. Sub-clusters 1, 3, and 5 combined BCR responses with MHC class II and cytokine signaling genes. In contrast, sub-cluster 4 showed downregulation of IL-4/STAT6 and CD40L programs while upregulating *PRDM1*, *CD27*, and UPR- and secretion-related pathways, consistent with an early pre-plasmablast state poised for terminal differentiation that retained IL-21 responsiveness, reinforcing the early role of IL-21 in plasmablast differentiation.^24^

Together, these analyses reveal that early B cell activation is characterized by combinatorial cytokine and receptor inputs that generate a continuum of transcriptional states, thereby establishing early lineage bias before overt plasma cell commitment.

### Transcriptional bifurcation at day 8 uncovers early co-expression modules priming plasma cell commitment

To investigate the transcriptional bifurcation at D8, we reconstructed differentiation trajectories using Monocle3, with D6 proliferative cells set as the root. Three distinct fates emerged: terminal PC differentiation (Fate 1), MBC differentiation (Fate 2), and a GC-like state enriched in D8 cells (Fate 3) (Figures 3A and S3A). The bifurcation occurred within cluster 4, identifying D8 as a key transition point between antibody-secreting and non-secreting lineages (Figure 3B).

**Figure 3:**
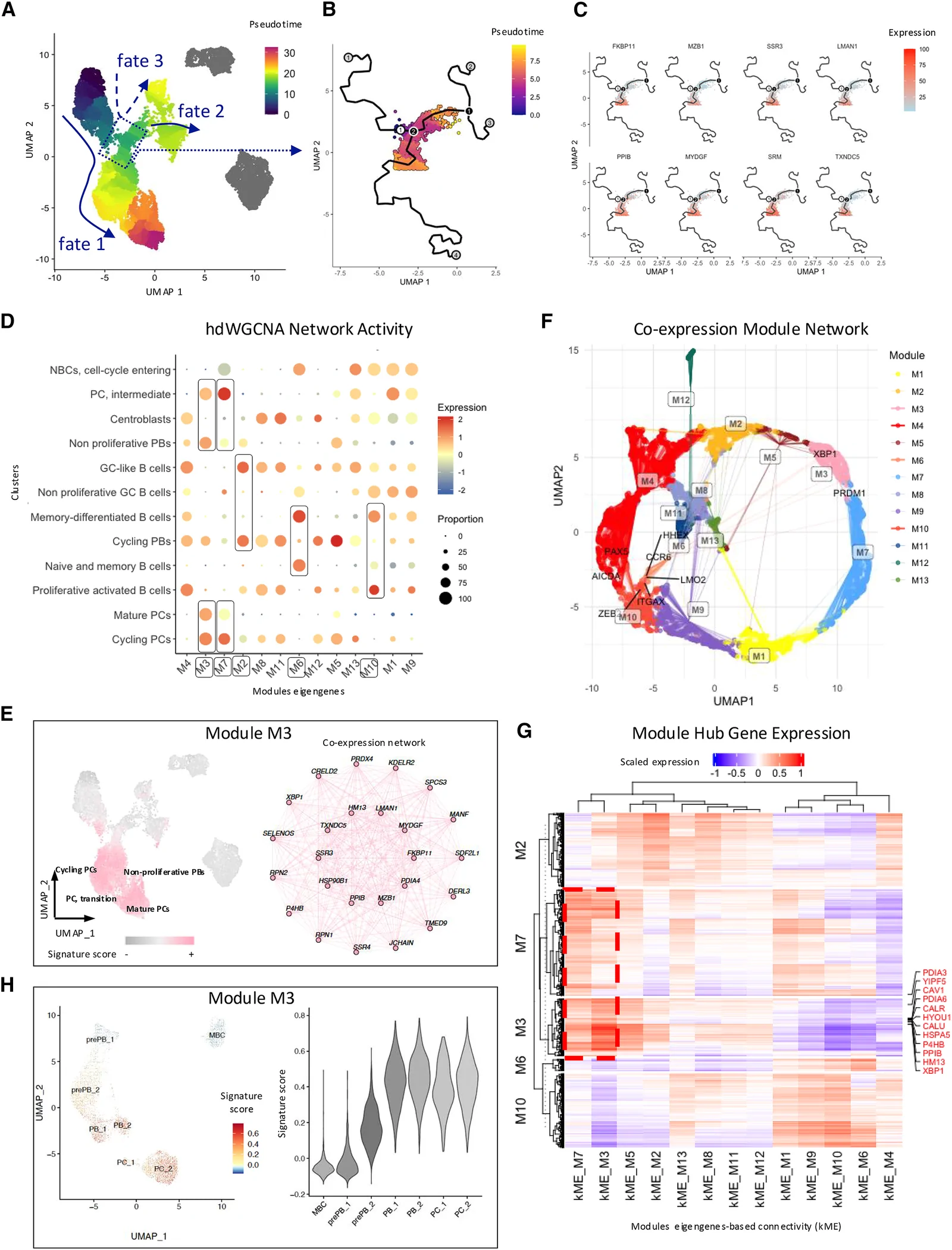
Pseudotime trajectory and hdWGCNA gene co-expression modules defining B cell differentiation (A) UMAP of Monocle3 pseudotime shows three different fates (Fate 1–3). (B) Pseudotime ordering of day-8 cells at the bifurcation point between two branches. (C) Pseudotime expression dynamics of representative Fate 1-marker genes illustrating the day-8 bifurcation fork. (D) Dot plot of module-specific hub genes (hdWGCNA) across clusters (dot size: % expressing cells; color: average expression). Key gene programs are associated with germinal center (GC) B cells, proliferating plasma cells (PCs), memory B cells, and naive B cells. (E) M3 module score on UMAP (left) and M3 co-expression network showing top 35 hub genes (right). (F) UMAP of module eigengenes showing inter-module relationships connections. (G) Heatmap of kME (eigengene-based connectivity) values across modules. Higher kME values indicate stronger intramodular network connectivity and highlight shared hub genes between modules M7 and M3 (red-dashed square). Module eigengene expression reveals twelve XBP1 target genes involved in the ERAD pathway are specifically enriched within M3. (H) Projection of the M3 module score on the UMAP (left) and violin plots (right), showing enrichment across differentiation stages in an independent *in vitro* plasma cell dataset.

Differential expression analysis identified 26 genes distinguishing Fate 1 from alternative branches (Figure S3B), including eight genes associated with early PC commitment (Figure 3C). Notably, canonical PC regulators such as *PRDM1* and *IRF4* were not yet robustly induced at this stage, indicating that Fate 1 cells represent a transcriptionally primed state preceding overt commitment (Figures S3B and S3C).

To capture coordinated transcriptional programs beyond individual genes, we applied high-dimensional weighted gene co-expression network analysis (hdWGCNA), identifying 13 co-expression modules (Figures 3D and S3D and Table S4). Five were strongly fate-associated: M3 and M7 with PCs, M6 and M10 with MBCs, and M2 with GC-like cells (Figure 3D). Each module was represented by its 10 highly connected hub genes and 15 peripheral interactors within the co-expression network (Figures 3E and S3E). Network topology revealed that 7 of the 10 hub genes of module M3 overlapped with Monocle-defined Fate 1 genes, reinforcing the association of M3 with early PC priming. The remaining hub gene, *SRM*, displayed transient expression at the bifurcation point before declining in mature PCs. *SRM* is part of the MYC-v1 gene signature, which was enriched in Fate 1 cells, consistent with MYC-driven proliferation preceding its repression by BLIMP1 during terminal PC differentiation (Figures S3B and S3F).^25^^,^^26^ In contrast, Fate 3-committed cells maintained expression of both *SRM* and *MYC*.

Module topology positioned M3 between PC-associated (M7) and GC-like (M2) modules, reflecting its intermediate role in the transition toward ASCs (Figure 3F). Gene-level clustering confirmed proximity between M3, M7, and M2, whereas memory-associated modules M6 and M10 clustered together (Figure 3G).

Consistent with the known heterogeneity of MBC populations,^27^^,^^28^^,^^29^^,^^30^ module M10 was separated from GC-like M2 by an intermediate activated module (M4) and was enriched for *ITGAX* (CD11c) and *ZEB2*, while lacking *CD27* and *CR2* expression— features consistent with an activated B cell state capable of secondary ASC differentiation^31^^,^^32^ (Figure S3G and Table S4).

Functional annotation of the PC-associated M3 module revealed enrichment for genes involved in membrane organization, intracellular trafficking, and organelle remodeling, processes largely governed by the XBP1 transcriptional program driving ER expansion during PC differentiation (Figure S3H). Among M3 genes, *CAV1* emerged as a previously unrecognized component in human PC differentiation (Figure 3G). *CAV1* expression was highest in PC-associated clusters 0 and 1 (Figures 1E and S3I), and analysis of an independent dataset^15^ further confirmed progressive induction of M3 genes toward PC states, concomitant with downregulation of memory-associated modules (Figures 3H, S3J and S3K).

Together, these analyses identify D8 as a pivotal branching point in human B cell differentiation, where fate-associated transcriptional modules encode early PC priming before overt induction of canonical regulators. Among these, the XBP1-linked M3 module—containing *CAV1*—marks early architectural remodeling of the emerging PC lineage.

### XBP1-dependent induction of *CAV1* accompanies plasma cell priming

Within the XBP1-enriched M3 module, *CAV1* emerged as one of the most highly expressed genes in PC-associated clusters 0 and 1 (Figure S3I). To determine whether *CAV1* is directly regulated by XBP1, we performed chromatin immunoprecipitation followed by quantitative PCR (ChIP-qPCR) using an anti-XBP1 antibody on differentiating B cells collected at D5 and D7 of the two-step PC differentiation culture system.^16^ Using primers flanking predicted XBP1-binding motifs identified in the HOCOMOCO database (Figures 4A, S4A and S4B), we detected significant enrichment of XBP1 occupancy at a site located approximately 450 bp upstream of the *CAV1* transcription start site in D7 cells (Figure 4B). This enrichment was comparable to that observed at the canonical XBP1 target *HSPA5* and was absent at a negative control locus (Figure S4C), consistent with direct XBP1 occupancy at the *CAV1* promoter.

**Figure 4:**
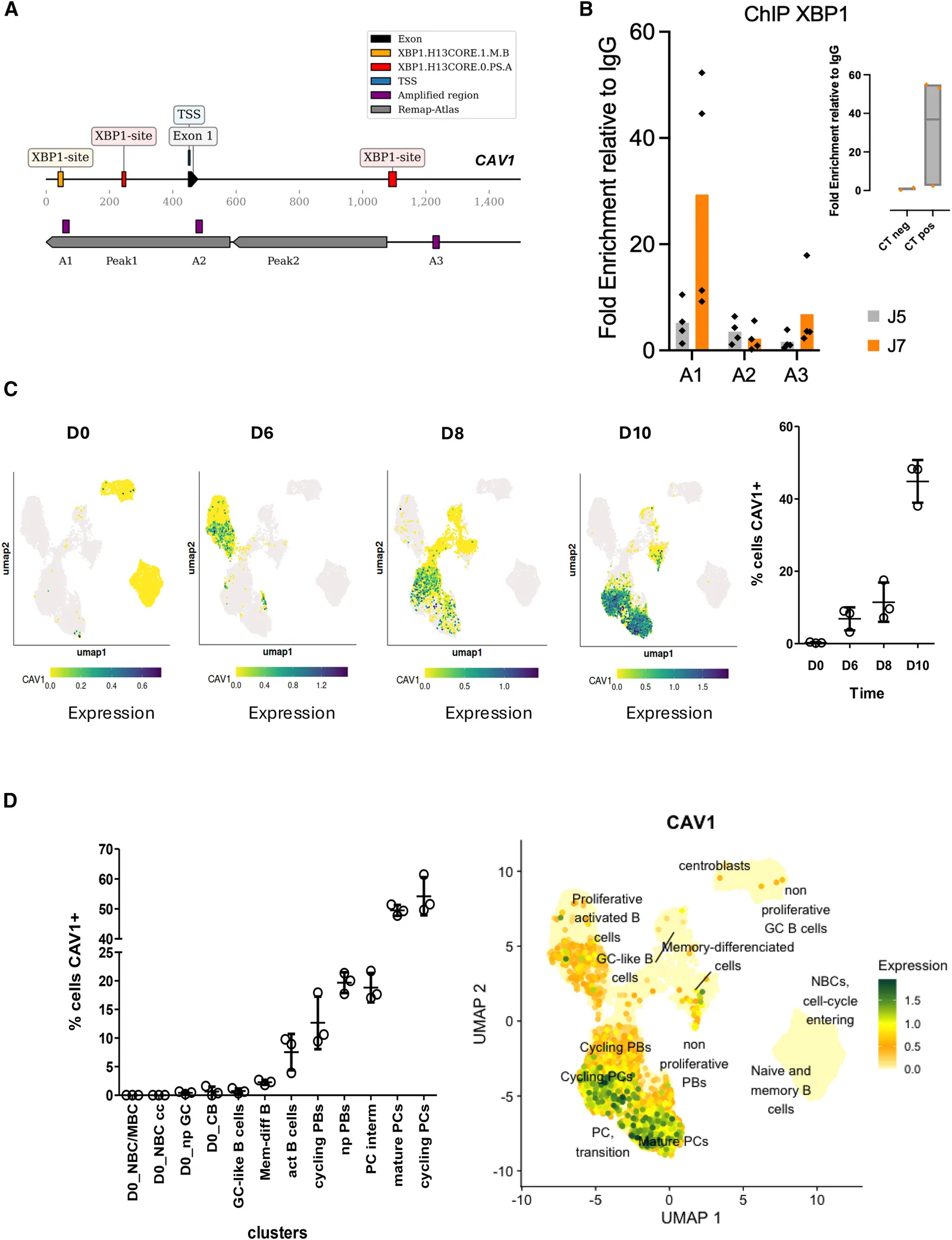
*CAV1* expression and XBP1-mediated regulation during B cell differentiation (A) Schematic representation of the *CAV1* locus (−500 bp to +1,500 bp relative to the transcription start site, including Exon 1). XBP1 consensus binding motifs (XBP1.H13CORE.1.M B and XBP1.H13CORE.0.PS) were identified using HOCOMOCO (HOmo sapiens COmprehensive MOdel COllection). Purple boxes indicate amplified regions for ChIP-qPCR. Peak1/Peak2 indicate ChIP-seq TF-binding-dense regions (ReMap Atlas). (B) XBP1 binding to the *CAV1* promoter assessed by ChIP-qPCR (A1–A3), with negative (neg CT) and positive (pos CT, *HSPA5*) controls. Enrichment is expressed as % input relative to the IgG control from four independent experiments. (C) UMAP representation of total *CAV1* expression dynamics. Data are derived from scRNA-seq of *in vitro*-differentiated B cells from three independent donors. The percentage of CAV1^+^ cells at each time point is indicated. Data are represented as mean ± SD. (D) Percentage of CAV1^+^ cells across identified cell subsets, and a colored representation of the CAV1 expression gradient is projected onto the UMAP (right image). Data are represented as mean ± SD.

Consistent with this regulatory relationship, *CAV1* transcripts were undetectable in resting B cells (D0) but became detectable in a small subset of activated cells by D6. *CAV1* expression then increased sharply at D8 and D10 along the pseudotime trajectory toward PC fate (Fate 1), reaching its highest levels in both proliferating plasmablasts and mature plasma cells (Figures 4C and 4D). This expression dynamic was independently reproduced in a separate scRNA-seq dataset of MBC-to-PC differentiation (Figure S4D), supporting the association of *CAV1* induction with the PC lineage.

Together, these findings indicate that the M3 module captures a core XBP1-linked transcriptional program, encompassing protein processing and membrane dynamics. The progressive induction of *CAV1*—previously unlinked to human PC differentiation—suggests that membrane-associated factors are integrated into the XBP1-dependent program that accompanies organelle expansion and secretory specialization.

### Biphasic Cav-1 expression marks early activation and plasma cell differentiation in human B cells

To place our findings on *CAV1* expression within a broader cellular context, we analyzed publicly available scRNA-seq datasets spanning multiple human tissues and B cell states. Consistent with prior reports, *CAV1* expression was prominent in endothelial cells, adipocytes, and smooth muscle cells, but largely absent from immune cells (Figure S5A).^33^ However, analysis of independent datasets revealed clear *CAV1* expression in tonsillar plasmablasts (Figure S5B)^34^ and bone marrow PCs (Figure S5C).^35^ In contrast, murine PCs lacked *Cav1* expression^36^ despite reports of transient *Cav1* induction in mouse B cells following LPS stimulation^37^ (Figure S5D), indicating that Cav-1 protein expression in ASCs represents a human-specific feature.

In our *in vitro* differentiation system, *CAV1* expression followed a biphasic pattern. Bulk RNA-seq using a simplified model of naive B cell differentiation discriminating pre-plasmablasts from MBC-like B cells based on CD23 expression (Figure 5A), revealed a transient induction of *CAV1* transcripts within 24 h of activation, followed by rapid downregulation and subsequent re-expression at days 5–6 during pre-plasmablast and plasmablast differentiation (Figures 5A and S5E). This early pulse was observed following activation of both IgM^+^ and class-switched memory B cells in previous RNA-seq data.^14^ By D6, *CAV1* expression levels were comparable to those in tonsillar plasmablasts (Figure 5A). Protein analyses confirmed this dynamic pattern and revealed that *in vitro*-generated PCs exclusively expressed the β-isoform of Cav-1 (∼19 kDa) (Figure 5B). *Ex vivo*, *CAV1* expression was restricted to tonsillar plasmablasts and bone marrow PCs, with plasmablasts expressing exclusively the β-isoform and mature PCs displaying low-level α-isoform transcripts (Figures 5C and S5F).

**Figure 5:**
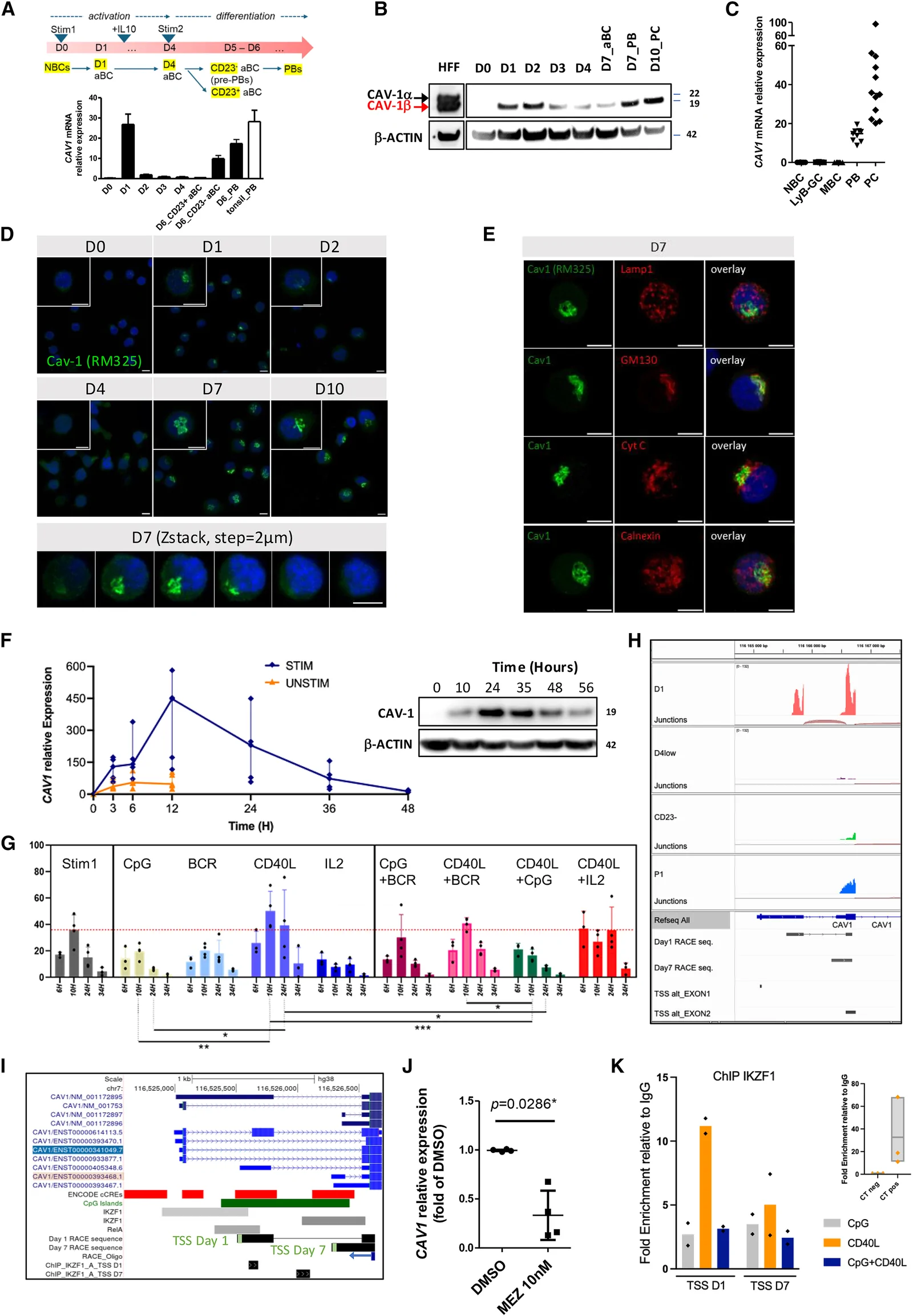
Biphasic Cav-1 expression marks early activation and plasma cell differentiation in human B cells (A) *Upper* image*,* Schematic overview of the *in vitro* naive B cell differentiation model used for bulk RNA-seq and functional assays. *Lower* image*,* RT-qPCR validation of total *CAV1* (all isoforms) expression levels during B cell differentiation compared to tonsil plasmablasts (*n* = 3). (B) Western blot detection of Cav-1 protein during B cell differentiation. Human fibroblasts (HFF) lysates were used as positive control. (C) Expression of total *CAV1* (all isoforms) in *in vivo* B cell subsets. (D) Immunofluorescence and z stack analysis of Cav-1 (green, RM325 antibody) localization at indicated time points; nuclei stained with DAPI (blue). Scale bars, 5 μm. (E) Co-staining of Cav-1 (green, RM325 antibody) with organelle markers (red): LAMP1 (lysosomes), GM130 (Golgi), CytC (mitochondria), and Calnexin (ER) in day 7 plasmablasts. Nuclei counterstained with DAPI. Scale bars, 5 μm. (F) Time-course of total *CAV1* (all isoforms) mRNA expression in unstimulated (UNSTIM) and stimulated (STIM) naive B cells (*n* = 5), and of Cav-1 protein expression after B cell activation (one representative experiment out of four). Data are represented as mean ± SD. (G) Relative *CAV1* expression (all isoforms) in B cells across multiple activation kinetics. Transcripts were measured by RT-qPCR at indicated time points (*n* = 3 to *n* = 4 independent donors per group). Data are expressed as mean ± SD relative to the unstimulated control. Statistical analysis was performed using a linear mixed-effects model with a Restricted Maximum Likelihood (REML) approach to account for inter-donor variability and the unbalanced experimental design, followed by Tukey’s post-hoc test for multiple comparisons (95% CI). Data are represented as mean ± SD. (H) IGV tracks of RNA-seq reads from differentiating naive B cells (D1, D4 proliferating D4lo, D5 pre-plasmablasts CD23^−^, plasmablasts P1) aligned to hg19. D1 shows reads in unannotated regions. (I) UCSC genome browser view of 5′ *CAV1* region (hg38). Novel TSS at D1 and previously published at D7 identified by 5′RACE-PCR (green line upstream the black boxes); reverse primer indicated by blue arrow. Aggregated IKZF1 and RelA ChIP-seq regions (gray), predicted regulatory elements (orange/red, ENCODE) and CpG islands (green). Position of ChIP-IKZF1 amplified regions for qChIP-PCR near the TSS at Day1 and Day7 are indicated. (J) Total *CAV1* (all isoforms) mRNA (RT-qPCR, right) after 16 h stimulation ± Mezigdomide (MEZ) or vehicle (DMSO). Mann-Whitney test (*n* = 4). Data are represented as mean ± SD. (K) ChIP-qPCR analysis of Ikaros binding at the TSS Day1 and TSS Day7 *CAV1* loci vs. IgG in B cells stimulated for 16 h with CpG, CD40L, or both (*n* = 2 experiments). The inset image show ChIP-qPCR of negative (genomic desert region) and positive (promoter of *MS4A1* gene) controls. Abbreviations: D, day; NBC, naive B cells; aBC, activated B cells; PB, plasmablasts; B-GC, germinal center B cells; MBC, memory B cells; BM-PC, bone marrow plasma cells; “n” values represent the number of independent donors and experiments. *p* ≤ 0.05 (∗), *p* ≤ 0.01 (∗∗); *p* ≤ 0.001 (∗∗∗); and *p* ≤ 0.0001 (∗∗∗∗).

Single-cell analyses further resolved the differentiation-stage specificity of *CAV1* expression. At D6, *CAV1* was strongly enriched in the pre-plasmablast subcluster, where over 90% of *CAV1*^+^ cells co-expressed *PRDM1*, whereas subclusters dominated by CD40L- or cytokine-responsive programs (subclusters 1, 3 and 5) displayed greater heterogeneity (Figure S5G). At D10, *CAV1*^+^ cells were also detected among MBCs, with approximately 40% co-expressing *PRDM1* (cluster 5), consistent with a population transcriptionally primed for PC differentiation.

Despite robust *CAV1* expression, neither activated B cells nor plasmablasts expressed key components required for caveolae formation, including Cavin family proteins and EHD2, across bulk and single-cell datasets (Figures S5H and S5I). Immunofluorescence analyses using antibodies targeting either the N- or C-terminal regions of Cav-1 revealed predominantly intracellular localization at D1, D7 and D10 of differentiation, with consistent co-localization with the Golgi marker GM130 and limited plasma membrane association detectable only with the N-terminal antibody (Figures 5D, 5E and S5J). No co-localization with ER, mitochondrial, or endolysosomal markers was observed. A similar non-caveolar, isoform-specific expression pattern was observed in multiple myeloma cell lines, although Cav-1α expression and membrane localization varied between lines (Figures S5K–S5M).

The reproducible detection of *CAV1* transcripts at very early stages of B cell activation prompted us to investigate the regulatory basis and functional relevance of this initial expression pulse. *In vitro* activation of naive B cells triggered rapid *CAV1* induction, peaking at approximately 12 h at the transcript level and 24 h at the protein level, before declining within 48 h (Figure 5F). Dissection of activation signals revealed CD40L as the dominant driver of early *CAV1* induction, whereas BCR stimulation had minimal effect and TLR9 engagement markedly suppressed CD40L-driven expression (Figure 5G). Analysis of transcript structure demonstrated that early and late *CAV1* expression arose from distinct transcript variants, although all encoded the β-isoform of Cav-1 (Figures 5H, 5I, and S5N).

Integration of ENCODE datasets identified binding sites for IKZF1 (Ikaros) and NF-κB p65 within the *CAV1* promoter, with RelA occupancy restricted to early activation (Figure 5I). Pharmacological degradation of Ikaros with Mezigdomide^38^ significantly reduced CD40L-induced *CAV1* expression (Figures 5J and S5O), and ChIP-qPCR confirmed Ikaros binding to the *CAV1* locus following CD40L stimulation, an interaction attenuated by TLR9 signaling (Figure 5K).

Despite its robust induction, the functional relevance of this transient Cav-1 pulse during early B cell activation remains unclear. Whereas Cav-1 regulates Syk activation upon BCR clustering in mice,^39^ phosphoflow cytometry experiments revealed that *CAV1* silencing did not affect BCR-proximal signaling after human B cell activation (Figures S5P and S5Q). Silencing *CAV1* also failed to alter phosphorylation of STAT5, STAT3, ERK1/2, p65, or AKT (Figure S5R), pathways in which Cav-1 has been implicated in other contexts.^40^ Because *CAV1* knockdown reduces immune synapse formation in chronic lymphocytic leukemia cells,^41^ we tested whether Cav-1 plays a similar role in nonmalignant activated B cells. After co-culture with stimulated allogeneic CD4^+^ T cells in the presence of a superantigen, flow cytometry across time showed no difference in T:B conjugate formation after CAV1 silencing (Figure S6S). Similarly, *CAV1* knockdown did not impact B cell proliferation or plasmablast differentiation (Figure S6T).

Together, these data identify two temporally and mechanistically distinct *CAV1* expression programs in human B cells: an early, CD40L- and Ikaros-dependent but functionally silent activation-associated pulse, and a later, sustained, non-caveolar β-isoform-restricted expression tightly linked to PC differentiation. These findings position Cav-1β as a differentiation-associated component of the human PC program, distinct from both canonical Caveolin biology and early activation responses.

### Cav-1 modestly restrains plasma cell differentiation and regulates adhesion-associated gene expression

To assess whether Cav-1 contributes functionally to PC differentiation, we silenced *CAV1* in pre-plasmablasts at D5 using siRNAs. This approach efficiently reduced *CAV1* mRNA and Cav-1 protein levels by D7 compared with scrambled controls (Figure S6A). Cav-1 knockdown did not affect cell viability but resulted in a modest yet reproducible increase in the frequency of *in vitro*-produced CD138^+^ PCs (Figure 6A). This increase was not accompanied by a significant change in total immunoglobulin secretion measured by ELISA, although ELISPOT analysis showed a trend toward an increased number of ASCs (Figures 6B and S6B). Together, these findings indicate that Cav-1 is largely dispensable for acute antibody production under these experimental conditions, while modestly influencing the efficiency of PC differentiation.

**Figure 6:**
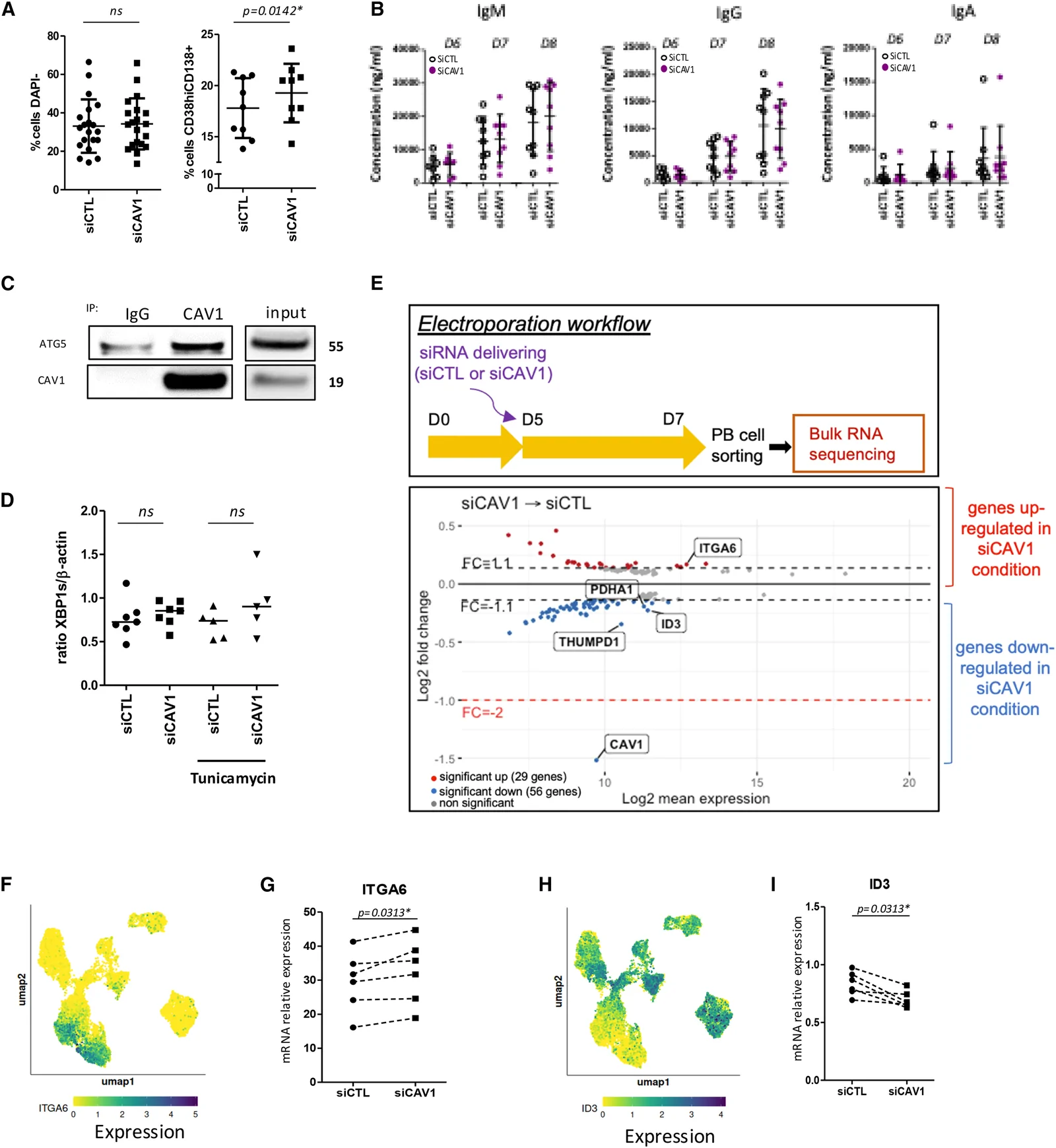
Cav-1 knockdown modestly alters plasma cell differentiation and associated gene expression (A) Flow cytometric analysis of cell viability and PC differentiation following *CAV1* silencing. B cells were electroporated with control (siCTL, squares) or CAV1 (siCAV1, circles) siRNA on day 5 and analyzed on day 7. Left, percentage of viable cells (DAPI^−^; *n* = 20 independent experiments). Right, percentage of mature PCs (CD38^hi^CD138^+^; *n* = 9 independent experiments). *CAV1* silencing did not affect cell viability but resulted in a modest yet significant increase in the proportion of CD38^h^iCD138^+^ PCs. Each symbol represents one donor. *p* values were determined using the paired Wilcoxon test. ns, not significant. Data are represented as mean ± SD. (B) Quantification of immunoglobulin secretion following *CAV1* silencing. IgM, IgG, and IgA concentrations (ng/mL) were measured by ELISA in culture supernatants collected on days 6, 7, and 8 after electroporation with siCTL (squares) or siCAV1 (circles) on day 5 (*n* = 9 independent experiments). Each symbol represents one donor. Lines connect paired samples. No significant differences were detected between the two conditions. Data are represented as mean ± SD. (C) Co-immunoprecipitation of Cav-1 and ATG5 in day 7 plasmablasts. Endogenous Cav-1 was immunoprecipitated using a goat anti-human Cav-1 antibody or goat IgG isotype control. Immunoprecipitates were analyzed by immunoblotting with anti-ATG5 and anti-Cav-1 antibodies. INPUT corresponds to 10% of the total protein lysate. (D) Quantification of XBP1s protein expression. XBP1s/β-actin ratios were quantified by ImageJ from immunoblots of day 7 plasmablasts electroporated with siCTL or siCAV1 and treated, where indicated, with tunicamycin (5 μg/mL, 4 h) (*n* = 7 independent experiments). *p* values were determined using the paired Wilcoxon test. ns, not significant. (E) Transcriptomic consequences of *CAV1* silencing. Left, schematic of the experimental design. Right, MA plot showing differentially expressed genes 48 h after siRNA electroporation. Genes downregulated (fold change ≤ −1.1) are shown in blue and upregulated genes (fold change ≥1.1) in red. CAV1 was reduced by more than 2-fold following siRNA-mediated silencing. (F) UMAP visualization of *ITGA6* expression during B cell differentiation. (G) RT-qPCR validation of increased *ITGA6* expression following *CAV1* silencing. *ITGA6* mRNA levels were measured in day 7 plasmablasts after siCTL or siCAV1 electroporation. Each symbol represents one donor. *p* values were determined using the paired Wilcoxon test. (H) UMAP representation showing *ID3* expression during B-cell differentiation. *ID3* repression is associated with PC commitment. (I) RT-qPCR validation confirming decreased ID3 mRNA expression in day 7 plasmablasts following CAV1 silencing. Each symbol represents one independent donor. *p* values were determined using the paired Wilcoxon test. Abbreviations: ns, stand for no significant.

Given reports implicating caveolins in autophagy regulation in other cell types,^42^^,^^43^^,^^44^ and the established role of autophagy in sustaining long-lived PCs,^45^ we examined whether Cav-1 modulates autophagic activity in plasmablasts. Co-immunoprecipitation (co-IP) experiments in D7 plasmablasts revealed an interaction between Cav-1 and Atg5 (Figure 6C). However, functional assays assessing autophagic flux (Cyto-ID staining and LC3 lipidation) detected no significant differences following *CAV1* silencing, either at baseline or after rapamycin treatment (Figure S6C). These results suggest that Cav-1 association with the autophagy machinery does not translate into measurable functional effects in differentiating human PCs, consistent with the cell-type or context-specific of caveolin-dependent autophagy regulation, as previously observed in lung and endothelial cells.^43^^,^^46^

Because PC differentiation critically relies on the UPR, particularly through XBP1s, we next evaluated whether Cav-1 modulates ER stress signaling. In contrast to observations in metastatic tumor models where *CAV1* silencing enhances XBP1s induction under ER stress,^47^ depletion of Cav-1 in primary plasmablasts did not consistently alter XBP1s protein levels, either under basal conditions or following tunicamycin treatment (Figures 6D and S6D).

To determine whether Cav-1 influences transcriptional programs associated with PC identity, we performed bulk RNA-seq on sorted CD38^+^/CD20^low^ plasmablasts at D7 following *CAV1* knockdown. Using stringent statistical thresholds (fold change > 1.5, adjusted *p* value <0.01), no genes other than *CAV1* were significantly altered. Relaxing these criteria (fold change > 1.1, adjusted *p*-value < 0.05) revealed modest but reproducible transcriptional changes, with 29 genes upregulated and 56 downregulated (Figure 6E and Table S5). Several differentially expressed genes—including *DNAJB9*, *AGAP1*, *PLEKHA8*, and *ITGA6*—belonged to transcriptional modules associated with PC identity (modules M3 and M7). Among these, *ITGA6*, encoding integrin α6, was consistently upregulated upon *CAV1* silencing (Figures 6F and 6G). This observation aligns with scRNA-seq data showing *ITGA6* expression predominantly in D10 mature PCs and in a subset of MBCs primed for PC differentiation (Figures 6F and S5H). Given the reported role of integrin α6 in PC retention within the bone marrow niche,^48^ and its reduced expression in more motile multiple myeloma cells,^49^ these findings raise the possibility that Cav-1 contributes to the fine-tuning of adhesion-related gene expression during terminal differentiation.

Conversely, among downregulated genes, *ID3* emerged as a notable target, as its repression is associated with PC commitment^50^^,^^51^ (Figures 6H, 6I and S6E).

Together, these results indicate that Cav-1 does not function as a dominant transcriptional regulator of PC identity but instead fine-tunes differentiation-associated gene expression, potentially by modulating the expression of lineage- and niche-associated genes such as *ID3* and *ITGA6*.

### Cav-1β scaffolds organelle networks associated with nutrient sensing and mTORC1 signaling in human plasma cells

The β-isoform of Cav-1 lacks the Tyr14 phosphorylation site and is thought to form oligomeric assemblies associated with intracellular membranes, characterized by rapid turnover and non-caveolar functions.^52^ In Cav-1–rich tissues, such as cardiomyocytes, non-caveolar Cav-1 has been implicated in lipid trafficking, signaling as a regulator capable of direct interactions with multiple client proteins, and endocytosis, though its physiological relevance remains incompletely defined.^53^^,^^54^

Our data identify human PCs as a context in which Cav-1β is selectively and robustly expressed, providing an opportunity to explore its function outside canonical caveolae.

To define Cav-1β-associated protein networks, we performed co-IP followed by mass spectrometry in XG21 multiple myeloma cells, identifying 348 interacting proteins (Table S6). These interactors were enriched for components of the endoplasmic reticulum, Golgi apparatus, mitochondria, lysosomes, and secretory granules, and were functionally associated with translation, glycosylation, protein trafficking, oxidative phosphorylation, ER organization, the UPR, and mTORC1 signaling (Figure 7A). The most abundant interactor was Stomatin (STOM), a cholesterol- and sphingolipid-associated protein that shares structural and functional features with caveolins and may partially compensate for Cav-1β loss in PCs.^55^

**Figure 7:**
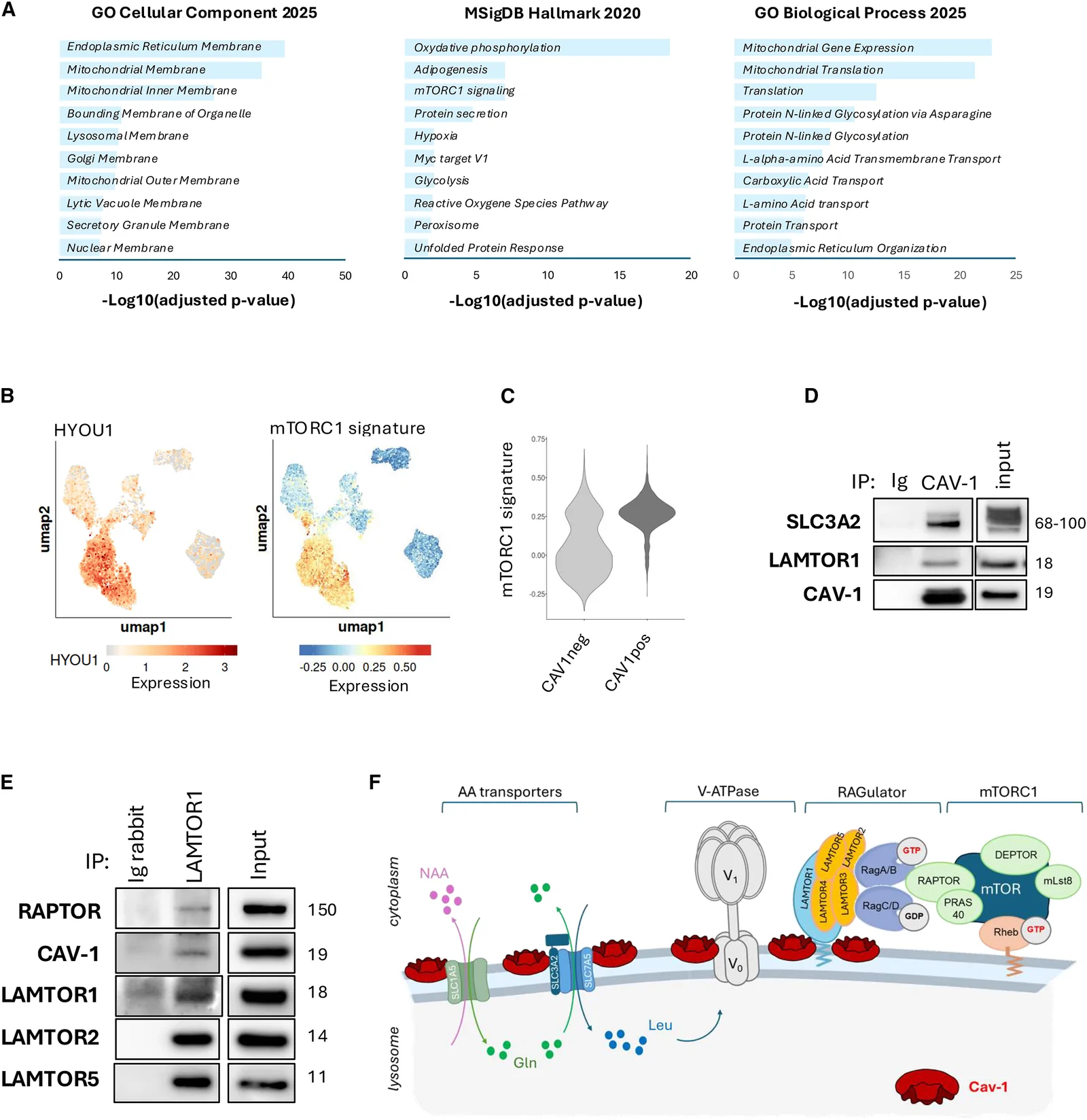
Cav-1β isoform connects organelle networks and the mTORC1 signaling platform (A) GO and MSigDB Hallmark enrichment of Cav-1 partners in the XG21 MM cell line. (B) UMAP plot of *HYOU1* expression (left) and mTORC1 signature (right) during B cell differentiation. (C) Violin plot mTORC1 signature enrichment in CAV1-positive cells. (D) Western blot of SLC3A2, LAMTOR1, and Cav-1 after Cav-1 immunoprecipitation in XG21 lysates (representative of three independent experiments). (E) Detection of Cav-1, RAPTOR, and RAGulator complex components (LAMTOR2 and LAMTOR5) after LAMTOR1 immunoprecipitation in XG21 lysates (representative of two independent experiments). (F) Schematic of Cav-1 interactions with lysosomal membrane proteins. Association with amino acid transporters and the V-ATPase/RAGulator suggests a role in mTORC1 recruitment and activation at the lysosomal surface. NAA, neutral amino acid; Gln, glutamine; Leu, leucine.

STRING network analysis revealed a high-confidence physical interaction network organized into 27 functional modules, with prominent clusters corresponding to mitochondrial translation and transport, the oligosaccharyltransferase (OST) complex, DNA replication, oxidative phosphorylation, and amino acid-regulated mTORC1 activation (Figure S7A). Notably, 28 Cav-1β interactors were encoded by XBP1 target genes within transcriptional module M3 (Figure S3H and Table S6), including key components of the OST complex and multipass translocon (DDOST, STT3A, SEC61A1), ER chaperones, and ER-associated degradation factors (DERL1/3), supporting a role for Cav-1β within the XBP1-driven ER remodeling program.

Among these interactors, Cav-1β is associated with HYOU1, an XBP1-regulated ER chaperone implicated in mTORC1 activation.^13^^,^^14^ HYOU1 expression was enriched in cells committed to the PC fate and closely paralleled the expression dynamics of *CAV1* and the mTORC1 gene signature (Figure 7B). Consistently, single-cell analyses demonstrated enrichment of mTORC1-associated transcriptional programs in *CAV1*^+^ cells (Figure 7C and Table S6). Cav-1β also interacted with SNAP23, an SNARE protein essential for vesicle fusion and immunoglobulin secretion,^56^ further linking Cav-1β to the secretory machinery of PCs.

At the lysosomal level, Cav-1β interacted with multiple components of the nutrient-sensing machinery upstream of mTORC1 activation, including the RAGulator complex (LAMTOR1/2/3), Rag GTPases (RRAGC/D), V-ATPase subunits, and the amino acid transporter CD98/LAT1 (SLC3A2/SLC7A5), which is critical for leucine and glutamine uptake in PCs.^57^ Additional interactors included LYCHOS (GPR155) and MCOLN1, regulators of cholesterol sensing, lysosomal function, and mTORC1 activation.^58^^,^^59^ Independent co-IP experiments validated key interactions within this network (Figures 7D and 7E), positioning Cav-1β as a scaffold-associated protein with amino acid-sensing and metabolic signaling platforms.^60^

Despite this extensive physical association with mTORC1-related machinery, functional assays revealed that Cav-1β is not required for acute amino acid-induced mTORC1 activation. In contrast to Caveolin-3 deficiency in skeletal muscle,^61^ depletion of Cav-1 in primary PCs or multiple myeloma cell lines did not markedly alter mTORC1 signaling, even under cholesterol-depleted conditions. *CAV1* silencing did not affect RAGulator assembly or RAPTOR recruitment (Figure S7B), and expression of Cav-1β in DG-75 B cells restored LAMTOR1 association without enhancing RAPTOR binding (Figure S7C). Thus, Cav-1β acts as a scaffold-associated protein with metabolic and secretory organelle networks rather than as a direct regulator of mTOR catalytic activity.

Together, these findings support a model in which Cav-1β integrates ER, Golgi, mitochondrial, and lysosomal protein networks in human PCs, positioning nutrient-sensing and stress-adaptive machineries in close proximity (Figure 7F). Although Cav-1β does not directly control mTORC1 activation, its sustained expression suggests that Cav-1β contributes to the architectural organization of the PC cytoplasm to balance secretory capacity, metabolic signaling, and differentiation state.

## Discussion

Single-cell analysis of human B cell differentiation reveals a continuum of activation states that resolve into divergent PC, MBC, and GC-like fates. Using a two-step stromal feeder-based culture system, we establish a robust *in vitro* model that recapitulates major transcriptional and phenotypic trajectories of human B cell differentiation. Single-cell profiling across sequential stages reveals a continuum of activation states that resolve into divergent PC, MBC, and GC-like fates. This system provides high-resolution insight into lineage decisions during human ASC differentiation and offers an experimentally tractable framework to investigate the cellular programs underlying human plasma cell commitment.

A key insight from this analysis is the pronounced heterogeneity observed during early B cell activation, particularly at D6, where transcriptionally distinct subclusters coexist within a single cluster. These subclusters integrate different combinations of CD40L, cytokine, MYC, and UPR-related programs, highlighting the plasticity of early activated B cells. Some cells retain strong proliferative and CD40L-responsive signatures, whereas others downregulate these pathways and initiate PRDM1-and XBP1-linked programs characteristic of pre-plasmablasts. This gradual and asynchronous reprogramming is consistent with previous studies showing that PC differentiation proceeds through a series of metastable states before irreversible commitment.^62^^,^^63^ These findings indicate that PC differentiation is initiated within a heterogeneous and metabolically dynamic landscape, in which lineage-specific programs are progressively consolidated rather than abruptly imposed.

By D8, following withdrawal of CD40L signaling, this heterogeneity resolves into a pivotal bifurcation, marking divergence toward three major fates: terminal PC differentiation, MBC differentiation, or a GC-like state. Network-level analysis revealed coordinated transcriptional modules that encode early fate bias. In particular, module M3 emerged as a transitional, PC-priming program positioned between GC-like and mature PC modules. M3 was enriched for XBP1-regulated genes involved in ER remodeling and membrane organization and showed strong overlap with genes defining the Monocle PC fate. The inclusion of *CAV1* within this module identified it as part of the transcriptional priming that precedes full PC maturation and suggested that membrane-associated scaffolding components may already be engaged during early commitment to the secretory lineage.

A notable finding is the biphasic regulation of *CAV1* during human B cell differentiation. *CAV1* expression occurs in two distinct phases: a transient, CD40L-driven pulse during the first 24 h of B cell activation, followed by stable expression during the plasmablast-to-PC transition. The early induction depends on CD40L and Ikaros and is transcriptionally distinct from the later PC-associated transcript. Despite prior reports in mice implicating Caveolin-1 in BCR nanoclustering through Tyr14 phosphorylation,^39^ the human Cav-1β isoform lacks this residue, and we find no evidence that transient Cav-1β expression affects BCR signaling, proliferation, T-B conjugate formation, or early differentiation. These findings suggest that early Cav-1β induction represents an activation-associated program without readily detectable functional consequences, possibly reflecting transient membrane remodeling or metabolic priming during the early phases of B cell activation.

In contrast, the later and sustained expression of Cav-1β is tightly linked to PC commitment. Cav-1β is a direct transcriptional target of XBP1 and is selectively expressed in human plasmablasts and PCs, where caveolae are absent due to lack of Cavin1 and EHD2. Proteomic analyses revealed that Cav-1β associates with a broad network of proteins spanning the ER, Golgi, mitochondria, lysosomes, and secretory machinery. These interactors are enriched for XBP1-regulated components involved in protein translocation, glycosylation, ER homeostasis, and vesicle trafficking, positioning Cav-1β as a structural component of the architectural remodeling that accompanies acquisition of the PC secretory state and suggesting a role in organizing membrane-associated protein networks within the expanding secretory cytoplasm.

A particularly notable feature of the Cav-1β interactome is its association with nutrient-sensing and mTORC1-related components, including the RAGulator complex, Rag GTPases, V-ATPase subunits, amino acid transporters, and the XBP1-regulated chaperone HYOU1. Consistent with these physical interactions, *CAV1*^+^ cells are enriched for an mTORC1 transcriptional signature. However, functional perturbation experiments demonstrate that Cav-1β is not required for acute mTORC1 activation in response to amino acids, nor for RAGulator assembly or RAPTOR recruitment. These findings argue against a direct regulatory role for Cav-1β in mTORC1 signaling. Instead, together with its extensive interactome, they are consistent with the hypothesis that Cav-1β contributes to the spatial organization of nutrient-sensing and stress-responsive protein networks within the expanding PC cytoplasm, while leaving the catalytic activity of these signaling pathways largely unaffected.

The modest yet reproducible effects of CAV1 silencing are compatible with a role for Cav-1β in intracellular organization rather than as a dominant lineage determinant. Cav-1 depletion slightly enhanced PC differentiation and altered the expression of a limited set of genes linked to lineage commitment and niche interactions, including downregulation of *ID3* and upregulation of *ITGA6*. Given the role of integrin α6 in PC retention within the bone marrow niche,^48^^,^^49^ these changes suggest that Cav-1β may influence aspects of differentiation, adhesion, or localization without being required for the overall PC fate decision.

Importantly, *CAV1* expression in PCs appears to be restricted to human PCs in our comparative analysis with murine PCs, which lack detectable Cav1 expression. This species difference suggests that alternative scaffolding mechanisms may operate in mice and highlights potential differences in PC architectural organization across species. Our human-based differentiation system therefore provides an experimentally accessible platform to investigate regulatory programs that are difficult to study directly in primary human PCs and may be particularly relevant to human diseases such as multiple myeloma or autoimmune disorders.

In summary, our study identifies Cav-1β as a component of the human PC differentiation program. We show that Cav-1β is induced downstream of XBP1, selectively expressed in human plasmablasts and PCs, and associated with an extensive network of ER, lysosomal, secretory, and nutrient-sensing proteins. Although functional perturbation revealed only modest effects on PC differentiation and no detectable role in acute mTORC1 activation or antibody secretion under our experimental conditions, our findings place Cav-1β within the membrane remodeling program that accompanies terminal PC differentiation.

Together, our data support a model in which Cav-1β is integrated into the specialized intracellular architecture of human PCs rather than functioning as a primary regulator of differentiation or mTORC1 signaling. Whether this association with membrane and nutrient-sensing networks contributes to the long-term organization of the secretory apparatus, metabolic adaptation, or persistence of long-lived PCs remains to be determined.

### Limitations of the study

Our study has several limitations that should be considered when interpreting the functional implications of Cav-1β during human PC differentiation. First, our experiments rely primarily on an *in vitro* differentiation system using human primary B cells, which provides experimental accessibility but does not fully reproduce the tissue microenvironment, cellular interactions, and long-term survival conditions experienced by PCs *in vivo*. Second, the selective expression of Cav-1β in human PCs, together with the limited availability and genetic tractability of primary human PCs, constrained our ability to perform extensive loss-of-function and long-term functional analyses in fully mature PCs. Consequently, our functional experiments primarily address early and intermediate stages of differentiation and acute responses rather than the maintenance and function of long-lived PCs. Third, although our proteomic analyses identify extensive associations between Cav-1β and ER, lysosomal, secretory, and nutrient-sensing proteins, these associations do not establish direct molecular interactions or demonstrate that Cav-1β is required for the activity of these pathways. Consistent with this limitation, CAV1 depletion produced only modest effects on PC differentiation and did not measurably affect acute mTORC1 activation or overall immunoglobulin secretion under our experimental conditions. Finally, the absence of detectable Cav-1 expression in murine PCs limits extrapolation of these findings to mouse models and suggests that the functional consequences of Cav-1β expression may depend on features of human PCs. Future studies using improved human PC models and approaches enabling long-term manipulation will be important to determine whether Cav-1β contributes to secretory adaptation, cellular maintenance, or PC persistence in physiological and pathological settings.

## Resource availability

### Lead contact

Further information and requests for resources and reagents should be directed to and will be fulfilled by the Lead Contact, Professor Thierry FEST (thierry.fest@univ-rennes.fr).

### Materials availability

All data supporting the findings of this study are available within the article and its supplementary materials. Additional information, materials, and reagents are available from the corresponding authors upon reasonable request.

### Data and code availability

• The single-cell RNA-sequencing data generated in this study have been deposited in the European Genome-phenome Archive (EGA) under accession code EGAS50000001470 (https://ega-archive.org/studies/EGAS50000001470) and dataset accession EGAD50000002118. Access to the controlled data is available through the EGA data access process. Bulk RNA-seq data comparing plasmablasts with or without CAV1 depletion are deposited in EGA under accession EGAS50000001471and accessible under the following link: https://ega-archive.org/studies/EGAS50000001471. Previously published datasets reanalyzed in this study are publicly available from GEO under the accession numbers cited in the text. The mass spectrometry proteomics data have been deposited in the PRIDE repository under accession code PXD079880 and are accessible under the following link: https://www.ebi.ac.uk/pride/archive/projects/PXD079880.
• This paper does not report original code.
• Any additional information required to reanalyze the data reported in this paper is available from the lead contact upon request.

## Acknowledgments

We thank all members of the Fest Laboratory for helpful discussions. We thank Garnett Kelsoe for providing us with the MS40L^lo^ human cell culture system. Cell sorting was performed at the Biosit CytomeTRI facility (UMS 6480 Biosit, Rennes, France). Immunofluorescence studies were conducted at the MRic photonics facility (UMS 6480 Biosit). We thank Johanna Bruce and Emilie-Fleur Gautier for the nLC-MS/MS experiments in the Proteom’IC facility, Université Paris Cité, CNRS, INSERM, Institut Cochin, F-75014 PARIS, France. This work was supported by an internal grant from the Service d’Hématologie Biologique, CHU Rennes, and by a specific grant for this research program from the Agence Nationale de la Recherche (ANR-19-CE15-0020-01). M.H. received a doctoral fellowship from FHU CAMIn and the Ligue Contre le Cancer (Comité d’Ille-et-Vilaine) and is supported by the “CCA–Inserm–Bettencourt Schueller” Fund. Proteom’IC facility, Université Paris Cité, CNRS, INSERM, Institut Cochin, F-75014 PARIS, France is supported by the DIM Thérapie Génique Paris Ile-de-France Région, IBiSA, and the Labex GR-EX. P.M. was granted by ANR-17-CE15-0009-01 (MoDEx-GC), and the Centre d'immunologie de Marseille-Luminy, which receives its core funding from Aix Marseille University, CNRS, and INSERM.

## Author contributions

G.C., F.D., and T.F. conceived the study. G.C., M.L., F.C., F.D., C.B., C.J., C.L., S.L., M.P., C.P., and T.F. developed the methodology. G.C., M.L., V.O., F.D., F.C., K.S., C.M., N.A., T.G., M.H., and M.C. performed the investigations. T.F. acquired funding and administered the project. G.C. and T.F. supervised the research. G.C., F.D., and T.F. wrote the original draft and the revised manuscript, and all authors reviewed and edited the final manuscript.

## Declaration of interests

The authors declare no competing interests.

## Declaration of generative AI and AI-assisted technologies in the manuscript preparation process

During the preparation of this work the authors used OpenAI’s ChatGPT to improve the clarity and quality of the English language. After using this tool, the authors reviewed and edited the content as needed and take full responsibility for the content of the published article.

## STAR★Methods

### Key resources table

**Table:** 

REAGENT or RESOURCE	SOURCE	IDENTIFIER
**Antibodies**		

anti-IgD (FITC, clone IA6-2),	BioLegend	cat# 348206
anti-CD3 (BV421, clone UCHT1)	BioLegend	cat# 300434
anti-CD38 (APC, clone HIT2),	BioLegend	cat# 303510
anti-CXCR4 (PE, clone 12G5),	BioLegend	cat# 306506
anti-CD83 (PE875Dazzle, clone HB15e),	BioLegend	cat# 306506
anti-CD10 (PE-Cy7, clone HI10a)	BioLegend	cat# 312214
anti-CD20-FITC (clone 2H7)	BioLegend	cat# 302304
anti-CD45-V500 (clone 2D1),	BD Biosciences	cat# 560779
anti-CD19-PE-Cy7 (clone SJ25C1),	BD Biosciences	cat# 557835
anti-CD20-BUV395 (clone L27),	BD Biosciences	cat# 740204
anti-CD23-BV605 (clone M-L233),	BD Biosciences	cat# 740414
anti-CD27-BV711 (clone M-T271),	BD Biosciences	cat# 564893
anti-CD38-PerCP-Cy5.5 (clone HIT2),	BD Biosciences	cat# 551400
anti-CD138-BUV737 (clone MI15)	BD Biosciences	cat# 612834
F(ab’)_2_ goat anti-human IgM (H + L) anti-IgM/IgG/IgA	Jackson Immunoresearch	cat# 109–006-129
anti-CD138-PE (clone 44F9)	Miltenyi Biotec	cat# 130–098-937
anti-IKZF1 (clone D6N9Y)	Cell Signaling Technology	cat# 14859
control IgG (clone DA1E)	Cell Signaling Technology	cat# 3900
anti-XBP1s (clone 143F)	BioLegend	cat# 647505
mouse IgG2a isotype control (clone MOPC-173)	BioLegend	cat# 400201
anti-Syk (pY348)-Alexa Fluor 488 (clone I120-722)	BD Biosciences	cat# 560081
anti-BLNK (pY84)-PE (clone J117-1278)	BD Biosciences	cat# 558442
anti-PLC-g2(pY759)-Alexa Fluor 488 (clone K86–689.37),	BD Biosciences	cat# 558507
anti-BTK (pY180)-PE (clone N35-86)	BD Biosciences	cat# 562753
anti-ERK1/2 (pT202/pY204)-Alexa Fluor 647 (clone 20A)	BD Biosciences	cat# 612593
CD79A (pY182)-Alexa Fluor 647 (clone D1B9).	Cell Signaling Technology	cat# 29742S
Cav-1 (goat pAb, AF-5736)	R&D Systems	cat# AF5736
α-Actin (mouse mAb, clone AC-15)	Sigma-Aldrich	cat# A5441
Ikaros (rabbit mAb, clone D6N9Y)	Cell Signaling Technology	cat# 14859
STAT5 pY694 (mouse mAb, clone 47/Stat5(pY694)	BD Biosciences	cat# 612567
STAT5 (mouse mAb, clone 89/Stat5)	BD Biosciences	cat# 610191
STAT3 pY705 (rabbit mAb, clone D3A7)	Cell Signaling Technology	cat# 9145
STAT3 (mouse mAb, clone 124H6)	Cell Signaling Technology	cat# 9139
ERK1/2 pT202/pY204 (rabbit mAb, cloneD13.14.4E)	Cell Signaling Technology	cat# 4370
ERK1/2 (rabbit mAb, clone 137F5)	Cell Signaling Technology	cat# 4695
NF-kB p65 S536 (rabbit mAb, clone 93H1)	Cell Signaling Technology	cat# 3033
NF-kB p65 (rabbit mAb, clone D14E12)	Cell Signaling Technology	cat# 8242
AKT pS473 (rabbit pAb, 9271)	Cell Signaling Technology	cat# 9271
AKT (rabbit pAb, 9272)	Cell Signaling Technology	cat# 9272
ATG5 (mouse mAb clone177.19)	BioLegend	cat# 847402
XBP1s (mouse mAb, clone 143F)	BioLegend	cat# 647502
RAPTOR (rabbit mAb, clone 24C12)	Cell Signaling Technology	cat# 2280
LAMTOR1 (rabbit mAb, clone D11H6)	Cell Signaling Technology	cat# 8975
LAMTOR2 (rabbit mAb, clone D7C10)	Cell Signaling Technology	cat# 8145
LAMTOR5 (rabbit pAb, A2606, 1107)	ABclonal	cat# A2606
SLC3A2(mouse mAb, clone 2B10F5)	Proteintech	cat# 66883-1-Ig
HRP Donkey anti-rabbit IgG	BioLegend	cat# 406401
HRP Goat anti-mouse IgG	BioLegend	cat# 405306
HRP Donkey anti-goat IgG	Bethyl Laboratories	cat# A50-100P
recombinant rabbit monoclonal anti-Cav-1 (clone RM325)	ThermoFisher Scientific	cat# MA5-33085
endoplasmic reticulum (clone AF18)	ThermoFisher Scientific	cat# MA3-027
anti-GM130 for the Golgi apparatus (clone 35)	BD Biosciences	cat# 610822
anti-cytochrome c for mitochondria (clone 6H2.B4)	BD Biosciences	cat# 556432
and anti-LAMP-1 for lysosomes (clone H4A3)	BD Biosciences	cat# 328601
donkey anti-rabbit AF488	Jackson ImmunoResearch	cat# 711–546-152
donkey anti-mouse AF647	Jackson ImmunoResearch	cat# 715–606-151
rabbit polyclonal anti-Cav-1	ABclonal	cat# A0059
goat anti-human Ig	Jackson ImmunoResearch Laboratories	cat# 109–005-064
HRP-coupled antibodies g chain	Jackson ImmunoResearch Laboratories	cat# 109–035-098
HRP-coupled antibodies a chain	Jackson ImmunoResearch Laboratories	cat# 109–035-011
HRP-coupled antibodies m chain	Jackson ImmunoResearch Laboratories	cat# 109–035-129
PE-conjugated goat anti-human IgA	SouthernBiotech	cat# 2050–09
PE-conjugated goat anti-human IgG	SouthernBiotech	cat# 2040–09
FITC-conjugated goat anti-human IgM	SouthernBiotech	cat# 2020–02

**Biological samples**		

Fresh tonsils	Pediatric tonsillectomies performed at the Clinique de La Sagesse (Rennes, France)	celine.pangault@univ-rennes1.fr
Human peripheral blood B cells	Buffy coats obtained from healthy blood donor volunteers recruited according to national French guidelines of the Établissement Français de Sang (EFS)	celine.pangault@univ-rennes1.fr

**Chemicals, peptides, and recombinant proteins**		

IL-4	*for GC B cell culture;* R&D Systems	cat# 204-IL
IL-10	*for GC B cell culture;* R&D Systems	cat# 217-IL
anti-BCR F(ab’)_2_ fragments	*for GC B cell culture;* Jackson ImmunoResearch	cat# 109–006-127
IL-21	*for GC B cell culture;* Miltenyi Biotec	cat# 130–095-768
recombinant human soluble CD40L	*for GC B cell culture;* NCI-Frederick	cat# D758060-X
IL-2	*for Tonsil B cell culture;* Proleukin	cat# 03400956215867
IL-21	*for Tonsil B cell culture;* PeproTech	cat# 200–21
IL-4	*for Tonsil B cell culture;* CellGenix	cat# 204-IL
BAFF	*for Tonsil B cell culture;* PeproTech	cat# 310–13
IL-6	*for Tonsil B cell culture;* PeproTech	cat# 200–06
CpG ODN2006	*for blood B cell culture;* Miltenyi Biotech	cat# 130–100-105
IFN-α	*for blood B cell culture;* R&D Systems	cat# 11105–1

**Critical commercial assays**		

Naive B Cell Isolation Kit II	Miltenyi Biotech	Cat# 130–091-150
B Cell Isolation Kit II	Miltenyi Biotech	Cat# 130–091-151
MojoSort Human Pan B Cell Isolation Kit	BioLegend	Cat# 480049
Chromium Next GEM Single Cell 5′ Reagent Kit v2	10× Genomics	Cat# 1000263
SMARTer RACE cDNA Amplification Kit	Takara Bio	Cat# 634923

**Deposited data**		

Mass spectrometry proteomics data	PRIDE repository	https://www.ebi.ac.uk/pride/archive/projects/PXD079880; accession code PXD079880
Single-cell RNA-seq data	European Genome-phenome Archive (EGA)	https://ega-archive.org/studies/EGAS50000001470; accession code EGAS50000001470 and EGAD50000002118-dataset accession
Bulk RNA-seq data	European Genome-phenome Archive (EGA)	https://ega-archive.org/studies/EGAS50000001471; accession code EGAS50000001471 and EGAD50000002119-dataset accession
Existing datasets that were used in the study	GEO repository	Alaterre et al.^15^ GSE242330 Hildreth et al.^33^ GSE155960 & GSE156110 King et al.^34^ GSE165860 Duan et al.^35^ GSE230705 Duan et al.^36^ GSE180920

**Experimental models: Cell lines**		

murine MS40L stromal cell line	Dr. David Baltimore’s lab and generously provided by Pr. Garnett Kelsoe	ghkelsoe@duke.edu
DG-75 (ACC 83)	DSMZ (Braunschweig, Germany)	cat# ACC 83
OPM2 (ACC 50)	DSMZ (Braunschweig, Germany)	cat# ACC 50
SKMM2 (ACC 430)	DSMZ (Braunschweig, Germany)	cat# ACC 430
U266 (TIB-196)	ATCC (American Tissue Culture Collection, Rockville, MD, USA)	cat# TIB-196
RPMI8226 (CCL-155)	ATCC (American Tissue Culture Collection, Rockville, MD, USA)	cat# CCL-155
XG2, XG7 and XG21 Multiple Myeloma cell lines (MMCLs)	Provided by Pr. Moreaux, University of Montpellier, France.	jerome.moreaux@igh.cnrs.fr

**Oligonucleotides**		

CAV1 A1 region: Forward GAAAACATTCGCTCTCCCCG/Reverse AAAGGTTTGTTCTGCTCGCG	XBP1_CHIP in this paper; Eurogenetec	N/A
CAV1 A2 region: Forward CAAATATCCCGACTGCTGCC; Reverse CCCAATCTCAGGACCCCAAT	XBP1_CHIP in this paper; Eurogenetec	N/A
CAV1 A3 region: Forward ATTGGGGTCCTGAGATTGGG; Reverse AATAACACCTCCACCCTGCA	XBP1_CHIP in this paper; Eurogenetec	N/A
HSPA5 promoter: Forward CAACCCCACTTACCAGGAGT; Reverse AGATGAAGCTCTCCCTGGTG.	XBP1_CHIP in this paper; Eurogenetec	N/A
TSS D1: Forward ACTCCTTCTGCATCTTGGCG; Reverse AGTGATGGTGGCAGTTTGGG	IKZF1 ChIP in this paper; Eurogenetec	N/A
TSS D7: Forward TAAAGGGAACATTCCACGGG; Reverse GCTTCACCTGTTTCGCGTT	IKZF1 ChIP in this paper; Eurogenetec	N/A
gene desert: Forward ACACACACCCTCACCTCCAT; Reverse CTTGCTCCCTCCTGCTTTCA	IKZF1 ChIP in this paper; Eurogenetec	N/A
MS4A1 promoter: Forward CCTACCATCCACACCTGCTTAT; Reverse TGGGCTTCGCTCCAATTACT	IKZF1 ChIP in this paper; Eurogenetec	N/A
CAV1 exon 2 (5′-TGTGCGCGTCGTACACTTGCTTCTCGCT-3′).	RACE in this paper; Eurogenetec	N/A
Primers for expression; see Table S7	LIFE TECHNOLOGIES SAS	https://www.thermofisher.com/taqman/gene-expression/assay

**Recombinant DNA**		

ON-TARGET plus Human CAV1 siRNA pool	Horizon Discovery	cat# L-003467-00-0005
ON-TARGET plus Non-targeting Control Pool	Horizon Discovery	cat# D-001810–10-05
CAV1β–Emerald fusion protein	Generously provided by Dr. Christophe Lamaze	christophe.lamaze@curie.fr
Empty control plasmid	Generously provided by Dr. Christophe Lamaze	christophe.lamaze@curie.fr

**Software and algorithms**		

R v4.5.1	Hornik et al.^64^	https://cran.r-project.org/
Seurat v4.3.0	Hao et al.^65^	https://satijalab.org/seurat/
10× Genomics Cell Ranger v76.1.1	Zheng et al.^66^	https://www.10xgenomics.com/support/software/cell-ranger/downloads
Single Cell Toolkit v2.18.0	Hong et al.^67^	https://www.camplab.net/sctk/
Monocle3	Trapnell et al.; Qiu et al.; Cao et al.^68^^,^^69^^,^^70^	https://cole-trapnell-lab.github.io/monocle3/
tradeSeq v1.5.10	Berge et al.^71^	https://statomics.github.io/tradeSeq/index.html
ClusterGVis	Jun et al.^72^	https://junjunlab.github.io/ClusterGvis-manual/
hdWGCNA	Morabito et al.^73^	https://smorabit.github.io/hdWGCNA/articles/basic_tutorial.html
ImageJ	Schneider et al.^74^	ImageJ.net/ij/download.html
STAR v2.5.2	The development of STAR is supported by the National Human Genome Research Institute of the National Institutes of Health	https://www.encodeproject.org/software/star/
featureCounts v1.5.0	Liao et al.^75^	https://subread.sourceforge.net/featureCounts.html
Prism v11.0.	GraphPad Software, Boston, Massachusetts USA, www.graphpad.com	https://www.graphpad.com
Perseus v3.0.1.0	Tyanova et al.^76^	N/A
FlowJo v10	BD Life Sciences, Ashland, OR, USA	https://flowjo.com/flowjo10/
GeneSys v1.9.2	Syngene, Cambridge, UK	https://www.synoptics.co.uk/syngene/support/software/

**Other**		

Illumina NovaSeq 6000	ICGex-NGS Platform, Institut Curie Genomics (Paris)	cedric.blouin@curie.fr; patricia.legoix@curie.fr
Dionex UltiMate 3000 HPLC nanoflow system (Thermo Fisher Scientific, Les Ulis, France) coupled to a TIMS-TOF Pro mass 1148 spectrometer (Bruker Daltonik GmbH, Bremen, Germany)	nLC–MS/MS analyses;Proteom’IC facility, Université Paris Cité, CNRS, INSERM, Institut Cochin, F-75014 PARIS, France	virginie.salnot@inserm.fr

### Experimental model and study participant details

#### Human tonsil specimens

Fresh human tonsils were obtained as residual surgical material from pediatric patients undergoing routine tonsillectomy. Samples were collected as residual surgical material and were irreversibly anonymized upon leaving the operating room, prior to any processing or analysis, precluding re-identification of individual donors. All samples corresponded to reactive tonsils from non-immunocompromised individuals. Because the specimens were irreversibly anonymized before receipt by the research laboratory, individual demographic information, including age, sex, gender, ancestry, race, and ethnicity, was not available to the investigators and could not be associated with experimental results. Accordingly, the potential influence of these variables on the experimental outcomes could not be assessed. No experimental allocation based on donor demographic characteristics was performed. Because the specimens were irreversibly anonymized before receipt by the research laboratory, written informed consent was not required under the applicable French regulatory framework (MR-003). No additional intervention or follow-up of the patients was performed for this study.

#### Human peripheral blood B cells

Human peripheral blood B cells were isolated from buffy coats obtained from healthy adult blood donors recruited through the Établissement Français du Sang (EFS) in accordance with applicable French national guidelines. Donors provided informed consent for the use of their blood cells for research. Buffy coats were provided to the investigators as donor-derived research material; demographic information regarding age, sex, gender, ancestry, race, and ethnicity was not available to the investigators or was not retained in a form linked to the experimental samples. Consequently, the potential influence of these variables on the experimental results could not be evaluated. No allocation of samples to experimental groups was performed on the basis of demographic characteristics. Individual donor samples were used as biological replicates, and paired experimental comparisons, where applicable, were performed using cells derived from the same donor.

#### Processing of human tonsil tissues and peripheral blood B cell

Tonsil tissues were mechanically dissociated through a 70-μm cell strainer in RPMI 1640 (Gibco) supplemented with 10% heat-inactivated fetal calf serum (FCS; Eurobio) and 1% penicillin–streptomycin (Gibco). Red blood cells were removed using EasyLyse Erythrocyte-Lysing Reagent (Dako).

For germinal center (GC) B-cell enrichment, total tonsil cells were first processed with the MojoSort Human Pan B Cell Isolation Kit (BioLegend, Cat#480049) according to the manufacturer’s instructions. To further enrich for GC B cells prior to flow-cytometric sorting, naive IgD^+^ B cells were depleted by adding a biotinylated anti-IgD antibody (clone IA6-2, BD Biosciences, Cat#555778) during magnetic separation.

For *in vitro* differentiation assays and single-cell transcriptomic analyses, total tonsil B cells were isolated by negative selection using the B Cell Isolation Kit II (Miltenyi Biotec), followed by magnetic separation with LS columns (Miltenyi Biotech).

Human peripheral blood B cells were isolated from buffy coats after Ficoll gradient centrifugation, naive B cells (NBCs; CD19^+^/CD27^-^) and total B cells (CD19^+^) were purified by negative selection using the Naive B Cell Isolation Kit II or B Cell Isolation Kit II (Miltenyi Biotech), followed by magnetic separation on the AutoMACS Pro using the “depletion sensitive” program (Miltenyi Biotech).

#### Cell lines

DG-75 (ACC 83), OPM2 (ACC 50), and SKMM2 (ACC 430) were obtained from the German Collection of Microorganisms and Cell Cultures (DSMZ, Braunschweig, Germany). RPMI8226 (CCL-155) and U266 (TIB-196) were obtained from the American Type Culture Collection (ATCC, Rockville, MD, USA). XG2, XG7, and XG21 multiple myeloma cell lines were provided by Jérôme Moreaux (University of Montpellier, France). The MS40L stromal cell line was originally established in the laboratory of Dr. David Baltimore and was generously provided by Prof. Garnett Kelsoe (Duke University, USA).

Cell line identity was authenticated by HLA sequencing and comparison with the expected HLA genotype. Cell lines were routinely tested for mycoplasma contamination using the MycoAlert Mycoplasma Detection Kit (Lonza) and were found to be negative.

Cell and multiple myeloma cell lines were cultured in RPMI 1640 GlutaMAX medium supplemented with 10% fetal calf serum (FCS) and antibiotics. For the IL-6-dependent XG cell lines, recombinant IL-6 (2 ng/mL; PeproTech) was added to the culture medium.

MS40L cells were expanded in Iscove’s Modified Dulbecco’s Medium (12440-053, Invitrogen) supplemented with 10% FBS, 1% penicillin-streptomycin (15140-122, Invitrogen), and 55 μM β-mercaptoethanol.

### Method details

#### Cell sorting of GC B cell subsets

Enriched tonsil B cells were resuspended in FACS buffer (PBS containing 2% FCS and 2 mM EDTA) and stained for 30 min at 4°C with the following fluorochrome-conjugated antibodies: anti-IgD (FITC, clone IA6-2), anti-CD3 (BV421, clone UCHT1), anti-CD38 (APC, clone HIT2), anti-CXCR4 (PE, clone 12G5), anti-CD83 (PE-Dazzle594, clone HB15e), and anti-CD10 (PE-Cy7, clone HI10a) (all from BioLegend).

Sorting was performed on a BD FACSAria IIu (BD Biosciences).

Sorted populations were defined as follows:

• Germinal center (GC) cells: CD3^−^ IgD^−^ CD38^+^ CD10^+^
• Centrocytes: CD3^−^ IgD^−^ CD38^+^ CD10^+^ CXCR4^-^ CD83^+^
• Centroblasts: CD3^−^ IgD^−^ CD38^+^ CD10^+^ CXCR4^+^ CD83^−^
• Plasmablasts: CD3^−^ IgD^−^ CD38^hi^
• Post-sort purity was >98% for all populations.

#### Cell culture of GC B cells and signature generation

To explore cytokine responses in GC B cell subsets, sorted GC cells were plated in 96-well round-bottom plates at 500 cells/well in 100 μL RPMI 1640 + 10% FCS +1% penicillin/streptomycin and cultured for 6 h under 31 distinct stimulation conditions in triplicate.

Stimuli included IL-4 (10 ng/mL; R&D Systems), IL-21 (50 ng/mL; Miltenyi Biotec), IL-10 (12 ng/mL; R&D Systems), recombinant CD40L (0.2 μg/mL; NCI-Frederick), and anti-BCR F(ab’)_2_ fragments (2.4 μg/mL; Jackson ImmunoResearch).

Following culture, cells were centrifuged, washed in PBS, and lysed in RLT buffer (Qiagen) before storage at −80°C. After thawing, mRNA was purified using AmpureXP beads (2.2× ratio) and eluted in 4 μL of FB5P-seq lysis buffer. The FB5P-seq protocol was performed as previously described^77^ with modifications: reverse transcription and cDNA amplification volumes were doubled (6 μL and 15 μL respectively), and PCR amplification was performed for 16 cycles instead of 22. Well barcodes identified culture conditions and replicates, while plate barcodes distinguished individual tonsil samples.

Data analysis was performed as previously described,^77^ after demultiplexing identified donors and culture conditions. Approximately 12,000 genes per well were detected on average (range: 8,000–15,000). Downstream analysis was performed using Seurat in R. Gene signatures were calculated using Seurat *FindMarkers*, retaining only positive marker genes with mean log fold change >0.25 and adjusted *p*-value <0.05. Individual stimulus signatures were determined by comparing conditions containing each stimulus versus all conditions lacking that stimulus.

#### *In vitro* tonsil B cell differentiation model

The murine MS40L stromal cell line was used as feeder support as described earlier.^78^ MS40L cells were irradiated at 70 Gy and plated at 3 × 10^4^ cells/cm^2^ in 6--well plates 4 h prior to coculture. Purified tonsillar B cells (40,000 cells/well/2 mL) were plated onto MS40L monolayers in complete RPMI 1640 containing 1% GlutaMAX, 1% penicillin/streptomycin, 10 mM HEPES, 1 mM sodium pyruvate, 1% MEM nonessential amino acids (all from Gibco), supplemented with 5% Human AB Serum (Sigma-Aldrich), IL2 50U/mL (Proleukin), IL4 10 ng/mL (CellGenix), IL21 10 ng/mL (PeproTech), and BAFF 10 ng/mL (PeproTech), locally named as R5-4C. Half of the medium containing cytokines was renewed once (day-3) during culture. At day-6, in a second culture phase, B cells were transferred on MS5 fibroblasts in R5-4C. Half of the medium was renewed at day-8 with addition of 20 ng/mL IL-6 (PeproTech) for 2 additional days to promote terminal differentiation.

Immunophenotyping at different time points was performed using anti- CD45-V500 (clone 2D1), CD19-PE-Cy7 (clone SJ25C1), CD20-BUV395 (clone L27), CD23-BV605 (clone M-L233), CD27-BV711 (clone M-T271), CD38-PerCP-Cy5.5 (clone HIT2), CD138-BUV737 (clone MI15), and Fixable Viability Stain 780 (FVS780), all from BD Biosciences, to monitor differentiation.

#### *In vitro* blood B cell differentiation model

Purified naive B cells (NBCs) or total peripheral blood B cells were cultured at 0.75 × 10^6^ cells/mL in 24-well plates and stimulated with *cocktail 1*. When necessary, cells were labeled with 1 μM Violet Proliferation Dye (VPD) 450 (BD Biosciences) to follow cell division. For NBCs, stimulation was performed using F(ab’)_2_ goat anti-human IgM (H + L) (Jackson ImmunoResearch), whereas total B cells were stimulated with F(ab’)_2_ anti-IgM/IgG/IgA (Jackson ImmunoResearch) at 2.4 μg/mL. Both cell types received recombinant human soluble CD40L (10 ng/mL; Immunex, NCI Frederick, MD, US), CpG ODN2006 (2 μg/mL; Miltenyi Biotech), and IL-2 (Proleukin 50 U/mL). After 2 days, antigen-primed B cells were supplemented with IL-10 (4 ng/mL; R&D Systems).

On Day 4, activated B cells were washed and recultured at 0.5 × 10^6^cells/mL with *cocktail 2* consisting of IL-2, IL-10 (12 ng/mL), and IL-4 (5 ng/mL; R&D Systems). At predefined time points, cells were collected, washed, stained, and either sorted based on fluorescence parameters or processed for downstream functional assays.

To generate plasma cells, on Day 7 viable total B cells were isolated by removing dead cells (Miltenyi Biotech) and then recultured with *cocktail 3*, composed of IL-2, IL-10, IFN-α (250 U/mL; R&D Systems), and IL-6 (20 ng/mL; PeproTech). Cells were again harvested at designated time points for flow cytometric analysis, sorting, or functional experiments. Differentiation and viability were followed by flow cytometry after staining with CD20-FITC (clone 2H7; Biolegend), CD38-APC (clone HIT2; Biolegend), CD138-PE (clone 44F9; Miltenyi Biotec) and DAPI (Sigma Aldrich) to exclude dead cells.

#### Single-cell RNA sequencing

A mean of 20,000 viable human B cells per donor (three biological replicates) were hashed with cell-surface oligo (HTO) barcodes and pooled at days 0, 6, 8, and 10 of culture. Single cell capture and cDNA library preparation were performed using the Chromium Next GEM Single Cell 5′ Reagent Kit v2 (10× Genomics) according to the manufacturer’s protocol. Libraries were sequenced on an Illumina NovaSeq 6000 (2 × 150 bp) with an average depth of ≥50,000 reads per cell.

Reads were processed using Cell Ranger v6.1.1 in intron-inclusion mode and aligned to the GRCh38 scRNA-seq–optimized transcriptomic reference v1.0 (10× Genomics), as described in Pool et al. (75). Samples were aggregated using the Cell Ranger aggr pipeline with default normalization, which equalizes the number of mapped reads per cell across libraries.

Cells were filtered out if they expressed <1,000 or >6,000 genes, >15,000 UMIs, or >10% mitochondrial reads. Doublets were identified and removed using the singleCellTK package v2.8.0. The median gene count per cell ranged from 5,400 to 9,800.

#### scRNA-seq data analysis

Filtered datasets were integrated using FindIntegrationAnchors and IntegrateData functions in the Seurat package (v4.3.0) in R (v4.5.1) (20 dimensions). PCA was followed by UMAP projection and clustering (30 nearest neighbors, resolution = 0.5). with a filtering step to removed Ig-genes, that were added back to the Seurat object for further analysis.

Differentially expressed genes were identified using FindAllMarkers functions from the Seurat package (Wilcoxon test). Cluster annotation was based on EnrichR analysis and enrichment of literature-derived B cell signatures calculated via AddModuleScore function from the Seurat package. For each gene signature, a score was calculated for every cell based on the median expression of the genes included in the signature. For M3, M6 and M10 signatures 295, 60 and 378 genes were used respectively. To account for background expression, scores were normalized using control gene sets automatically selected by Seurat.

For trajectory inference, Monocle3 was used with day 6 cells set as the trajectory root. Pseudotime-dependent expression dynamics were modeled with tradeSeq (fitGAM) using a negative binomial generalized additive model. Genes with adjusted *p* < 0.05 and |log_2_FC| > 0.5 were visualized by plotSmoothers function from tradeSeq package and pseudotime-ordered heatmaps function from ClusterGVis package.

#### Single-cell co-expression network analysis

Metacells were constructed using MetacellsByGroups function from the hdWGCNA package.^73^ Module networks were visualized with ModuleNetworkPlot function, displaying the top 35 hub genes per module (10 central, 25 peripheral).

Global hub-gene UMAPs were generated using HubGeneNetworkPlot function with 10% edge retention for clarity. Edge opacity reflects co-expression strength.

#### Chromatin immunoprecipitation (ChIP) and qPCR

##### XBP1 ChIP

Promoter and intragenic regions consensus binding motifs were retrieved from HOCOMOCO v13,^79^ and candidate regions in *CAV1* and *HSPA5* were selected (XBP1.H13CORE.1.M B and XBP1.H13CORE.0.PS). Primers flanking predicted sites were designed using Primer3 and synthesized by Eurogenetec: *CAV1* A1 region: Forward GAAAACATTCGCTCTCCCCG; Reverse AAAGGTTTGTTCTGCTCGCG; CAV1 A2 region: Forward CAAATATCCCGACTGCTGCC; Reverse CCCAATCTCAGGACCCCAAT; *CAV1* A3 region: Forward ATTGGGGTCCTGAGATTGGG; Reverse AATAACACCTCCACCCTGCA; Positive control (*HSPA5* promoter): Forward CAACCCCACTTACCAGGAGT; Reverse AGATGAAGCTCTCCCTGGTG.

ChIP was performed with the ChIP-IT High Sensitivity Kit (Active Motif) following the manufacturer’s protocol. Cells were cross-linked with 0.1% formaldehyde for 2 min at room temperature, quenched with 20 mM glycine, and lysed. Chromatin was sonicated (Covaris M220, 75 W, 5% duty cycle, 200 cycles/burst, 5 min) to 200–500 bp. Immunoprecipitations used anti-XBP1s (clone 143F, BioLegend) or mouse IgG2a isotype control (clone MOPC-173, BioLegend).

After reverse crosslinking, purified DNA was analyzed by qPCR (LightCycler 480, Roche) using SYBR Green detection. Primers specific for predicted XBP1 binding regions within the *CAV1* and *HSPA5* genes described above were used. Fold enrichment over input DNA (1%) was calculated relative to IgG controls.

##### IKZF1 ChIP

Cells were fixed with 1% formaldehyde for 10 min, quenched with 20 mM glycine, and lysed. Chromatin was sonicated using an EpiShear Probe Sonicator (Active Motif) (30 s on/off, 42% power, total 20 min). Immunoprecipitation was performed overnight at 4°C with anti-IKZF1 (clone D6N9Y, Cell Signaling Technology) or control IgG (clone DA1E, CST), followed by capture with protein A/G magnetic beads. Washes were performed in high-salt, low-salt, LiCl, and TE buffers.

DNA purification used the ChIP DNA Clean & Concentrator Kit (Zymo Research). Enrichment was quantified by SYBR Green qPCR (Applied Biosystems StepOnePlus) using selected primers for Region near TSS at D1: Forward ACTCCTTCTGCATCTTGGCG; Reverse AGTGATGGTGGCAGTTTGGG; for Region near TSS at D7: Forward TAAAGGGAACATTCCACGGG; Reverse GCTTCACCTGTTTCGCGTT. Primers for Negative control (gene desert): Forward ACACACACCCTCACCTCCAT; Reverse CTTGCTCCCTCCTGCTTTCA; and Positive control (MS4A1 promoter): Forward CCTACCATCCACACCTGCTTAT; Reverse TGGGCTTCGCTCCAATTACT were also selected. Data were normalized to input and expressed as fold enrichment relative to IgG control.

#### 5′ RACE PCR

Total RNA was extracted from B cells cultured for 1 or 7 days using NucleoSpin RNA Plus (Macherey-Nagel). 5′ RACE was performed with the SMARTer RACE cDNA Amplification Kit (Takara Bio) using a 3′-end primer targeting *CAV1* exon 2 (5′-TGTGCGCGTCGTACACTTGCTTCTCGCT-3′).

Amplified cDNAs were sequenced by Genewiz, and sequences were analyzed by BLAST to identify transcription start sites and compared against Ensembl, UCSC, and NCBI annotations to detect novel transcripts variants.

#### RNA-Seq analysis

On day 7, B cells previously electroporated on day 5 with siCTL or siCAV1 (*n* = 4 donors) were sorted as DAPI^−^ CD20^lo^ CD38^hi^ plasmablasts on a FACSAria IIu cell sorter (BD Biosciences) then processed for bulk RNA-seq. RNA integrity was verified using a Bioanalyzer 2100 (Agilent). Libraries were prepared and sequenced at Institut Curie (Paris) on an Illumina NovaSeq 6000 (2 × 100 bp; ≥50 million reads/sample; ≥80% ≥ Q30).

Reads were quality-checked using FastQC v0.11.5, aligned to GRCh38 (release 87) using STAR v2.5.2, and quantified with featureCounts v1.5.0.

Differential expression was analyzed in DESeq2 v1.22.2, with Benjamini Hochberg-adjusted *p* < 0.05 considered significant.

#### Quantitative RT-PCR analysis

RNA was extracted using a NucleoSpin® RNA Plus XS kit (Macherey-Nagel) and reverse transcribed to cDNA using a Superscript II kit (Invitrogen). RT-qPCR was performed using Applied Biosystem TaqMan (Thermo Fisher Scientific) Gene Expression Master Mix. Gene expression levels were quantified using *ABL1* as an endogenous control. The 2ˆ(-ΔΔCt) method was used to determine the relative expression of each gene.

TaqMan Gene Expression Assays (FAM) used is listed in Table S7.

#### Transient transfections

At Day 0 (D0) or Day 5 (D5) of the *in vitro* peripheral blood B cell differentiation culture, cells were collected and resuspended in 50 μL of MaxCyte electroporation buffer (sufficient for 8–10 × 10^6^ cells). Cell suspensions were supplemented with 320 pmol of ON-TARGETplus Human CAV1 siRNA (SMARTpool; Horizon Discovery) or a non-targeting control pool (Horizon Discovery). Electroporation was performed using the MaxCyte ATx instrument with the “B cell 2” program. Following a brief recovery at 37 °C, cells were counted and cultured in complete medium containing the appropriate cytokine cocktails for downstream functional assays or RNA sequencing.

The same electroporation protocol was applied to the XG21 plasma cell line, using the MaxCyte ATx “THP1” program.

DG75 cells were electroporated with either a plasmid encoding the CAV1β–Emerald fusion protein or an empty control plasmid (1 μg DNA) using the Nucleofector Kit V (Lonza) and the O-17 program on the Amaxa Nucleofector 1 device. After 48 h, transfection efficiency was assessed by flow cytometry, with >90% of cells expressing Emerald.

#### Protein extraction and western blot analysis

For total protein extraction, cell pellets were lysed in RIPA lysis buffer (Pierce) in the presence of protease inhibitors (PIC, 04693116001; Roche). After centrifugation, protein concentration was determined by the Bradford method (Biorad). Denatured protein extracts (at least 10 μg) were then separated on a NuPAGE 4–12% Bis-Tris acrylamide gel (Invitrogen) and transferred to nitrocellulose membranes using an iBlot 2 transfer device (Invitrogen). After blocking and incubation with specific primary antibodies, immunoblots were revealed with HRP-conjugated secondary mAb or, for immunoprecipitation, Clean-Blot IP Detection Reagent (Thermo Scientific; 21230) after addition of ECL plus (Thermo Scientific; 32132) or SuperSignal West Femto Maximum Sensitivity Substrate (Thermo Scientific; 34094) using the G:BOX Chemi imaging system (Syngene) and analyzed with GeneSys software. β-actin was used as loading control. Immunoblots shown in the figures were sometimes obtained with samples derived from the same experiment but processed on separate gels for different markers. When needed, membranes were stripped (Restored Plus Stripping buffer; Thermo Scientific), blocked and reprobed.

List of primary antibodies used: Cav-1 (goat pAb, R&D Systems, AF-5736); β-Actin (mouse mAb, clone AC-15, Sigma-Aldrich); Ikaros (rabbit mAb, clone D6N9Y, CST); STAT5 pY694 (mouse mAb, clone 47/Stat5(pY694), BD Biosciences); STAT5 (mouse mAb, clone 89/Stat5, BD Biosciences); STAT3 pY705 (rabbit mAb, clone D3A7, CST); STAT3 (mouse mAb, clone 124H6, CST); ERK1/2 pT202/pY204 (rabbit mAb, clone D13.14.4E, CST); ERK1/2 (rabbit mAb, clone 137F5, CST); NF-kB p65 S536 (rabbit mAb, clone 93H1, CST); NF-kB p65 (rabbit mAb, clone D14E12, CST); AKT pS473 (rabbit pAb, 9271, CST); AKT (rabbit pAb, 9272, CST); ATG5-HRP (mouse mAb clone 177.19, Biolegend); XBP1s (mouse mAb, clone 143F, Biolegend); RAPTOR (rabbit mAb, clone 24C12, CST); LAMTOR1 (rabbit mAb, clone D11H6, CST); LAMTOR2 (rabbit mAb, clone D7C10, CST); LAMTOR5 (rabbit pAb, A2606, ABClonal); SLC3A2 (mouse mAb, clone 2B10F5, Proteintech).

List of secondary antibodies used: HRP Donkey anti-rabbit IgG (Biolegend); HRP Goat anti-mouse IgG (Biolegend); HRP Donkey anti-goat IgG (Bethyl Laboratories).

#### Protein extraction and immunoprecipitation

Cells were lysed on ice in NP-40 lysis buffer (50 mM Tris-HCl, pH 7.5; 150 mM NaCl; 1% NP-40; 1 mM PMSF; 1 mM protease inhibitor cocktail). Lysates were cleared by centrifugation, and protein concentration was measured using the Bradford assay. For immunoprecipitation, ∼1 mg of total protein was incubated overnight at 4 °C with either a polyclonal goat anti-human Cav-1 antibody (R&D Systems, AF-5736) or a monoclonal rabbit anti-LAMTOR1 antibody (Cell Signaling Technology, clone D11H6). Immune complexes were captured by incubation for 1h at 4 °C with Dynabeads Protein G (Invitrogen).

Beads were washed four times using a DynaMag (Invitrogen) with NP-40 wash buffer (50 mM Tris-HCl, pH 7.5; 400 mM NaCl; 1% NP-40; 1 mM PMSF; 1 mM protease inhibitor cocktail). Antibody–protein complexes were resuspended in reducing sample buffer, heat-denatured, and placed on a magnet to recover supernatants for SDS–PAGE and immunoblot analysis.

#### Proteomic: Affinity purification-mass spectrometry

##### Immunoprecipitation

Dry pellets from 100 × 10^6^ XG-21 cells were lysed in 2 mL NP-40 lysis buffer supplemented with 1 mM MgCl_2_ and 1,500 U Benzonase (Merck). Lysates were cleared by centrifugation, and protein concentration was measured using the Bradford assay. For affinity purification, ∼4 mg of total protein was incubated overnight at 4 °C with 2 μg goat anti-human Cav-1 antibody (R&D Systems, AF-5736) (IP_CAV1) or goat IgG isotype control (IP_Ig), followed by a 1 h incubation with Dynabeads Protein G (Invitrogen) at 4 °C. Beads were washed sequentially: three times in wash buffer containing 1% NP-40, three times in wash buffer containing 0.1% NP-40, and three times in PBS. Bound proteins were eluted by resuspension in Laemmli buffer containing DTT, heat-denatured, and separated from beads using a magnet. Supernatants were collected and stored at −20 °C until mass spectrometry analysis.

##### Liquid chromatography coupled to mass spectrometry (nLC-MS/MS)

Co-immunoprecipitated samples were solubilized in lysis buffer (4% SDS, 400 mM Tris-HCl (pH 8.5), 20 mM TCEP, 100 mM chloroacetamide). Bottom-up experiments’ tryptic peptides were obtained by S-Trap Micro Spin Column according to the manufacturer’s instructions (Protifi, NY, USA). Briefly, proteins were digested during 14h at 37°C with 1 μg Trypsin sequencing grade (Promega). The S-Trap Micro Spin Column was used according to the manufacturer’s protocol. After speed-vacuum drying, eluted peptides were solubilized in 10% ACN and 0.1% TFA.

nLC–MS/MS analyses were performed on a Dionex UltiMate 3000 HPLC nanoflow system (Thermo Fisher Scientific, Les Ulis, France) coupled to a TIMS-TOF Pro mass spectrometer (Bruker Daltonik GmbH, Bremen, Germany). A total of 1 μL of each sample was loaded, concentrated and washed for 3 min on a C18 reversed-phase precolumn (3 μm particle size, 300 μm inner diameter, 5 mm length; Thermo Fisher Scientific). Peptides of IP eluates were then separated on an Aurora C18 reverse phase resin (1.6 μm particle size, 100 Å pore size, 75 μm inner diameter, 25 cm length) (IonOpticks, Middle Camberwell Australia) with a 60 min overall run-time gradient from 99% of solvent A containing 0.1% formic acid in milliQ-grade H2O to 40% of solvent B containing 80% ACN, 0.085% formic acid in mQH2O with a flow rate of 400 nL/min.

The mass spectrometer acquired data throughout the elution process in a positive mode and operated in DIA PASEF mode with a 1.89 s/cycle, with Timed Ion Mobility Spectrometry (TIMS) enabled. Ion accumulation and ramp time in the dual TIMS analyzer were set to 166 ms each. The MS1 spectra were collected in the m/z range of 100–1,700. The diaPASEF window scheme ranged in dimension 1/K0 from 0.60 to 1.60. The collision energy was set by linear interpolation between 59 eV at an inverse reduced mobility (1/K0) of 1.60 versus/cm2 and 20 eV at 0.6 versus/cm^2^.

##### Protein identifications and quantifications

The mass spectrometry data were analyzed using DIA-NN version 1.8.1.^80^ The database used for *in silico* generation of spectral library was a concatenation of Human sequences from the Swissprot database (release Mai 2022) and a list of contaminant sequences from Maxquant and from the cRAP (common Repository of Adventitious Proteins). M-Terminus exclusion and carbamidomethylation of cysteins was set as permanent modification and one trypsin mis-cleavage was allowed. Precursor false discovery rate (FDR) was kept below 1%. The normalization and the “match between runs” (MBR) option was allowed.

For downstream statistical analysis, DIA-NN outputs were imported into the Perseus software version 1.6.15.0. Reverse and contaminant proteins were excluded from analysis. For comparisons between IP_CAV1 vs. IP_Ig, two-sample t-tests (*p*-value) were performed on proteins present in at least 3/5 IP_CAV1, with LFQ intensities (IP_CAV1)>1000 and a ratio LFQ(IP_CAV1)/LFQ(IP_Ig) > 3. Additional t-tests with Benjamini-Hochberg FDR correction (q-value) were performed under the same criteria.

#### Immunofluorescence

Round Ø 14 mm glass coverslips were coated with 0.01% Poly-L-Lysine (Sigma Merck, #P8920) for 1h at room temperature and washed with PBS 1×. Cells were then seeded onto the PLL-coated coverslips in complete medium at a concentration of 2.10^6^ cells/mL and incubated for 1 h at 37 °C, 5% CO_2_. Cells were then fixed with 4% paraformaldehyde (PFA) for 10 min at room temperature. After washing with PBS 1×, nonspecific antibody-binding sites were blocked using a solution containing 1× PBS supplemented with 2% bovine serum albumin, 4% donkey serum, and 0.1% saponin for 1 h at room temperature.

Cells were incubated with primary antibodies overnight at 4 °C in a humidified chamber. Caveolin-1 was stained using either a rabbit polyclonal anti-Cav-1 (ABclonal, #A0059) or a recombinant rabbit monoclonal anti-Cav-1 (clone RM325, ThermoFisher Scientific, #MA5-33085). Intracellular compartments were stained using the following mouse monoclonal antibodies: anti-calnexin for the endoplasmic reticulum (clone AF18, ThermoFisher Scientific, #MA3-027), anti-GM130 for the Golgi apparatus (clone 35, BD Biosciences, #610822), anti-cytochrome c for mitochondria (clone 6H2.B4, BD Biosciences, #556432), and anti-LAMP-1 for lysosomes (clone H4A3, BioLegend, #328601).

After washing with PBS/0.1% saponin, cells were incubated for 1 h at room temperature with the fluorophore-coupled secondary antibodies, a Donkey anti-rabbit AF488 (Jackson Immunoresearch, #711–546-152) and a donkey anti-mouse AF647 (Jackson Immunoresearch, #715–606-151). Finally, coverslips were washed with PBS 1× and mounted onto glass slides using DAPI-Fluoromount-G® (SouthernBiotech; #0100-20).

Imaging was performed on an inverted confocal microscope using a Zeiss LSM880 Airyscan equipped with a 63×, 1,4 NA objective. Image analysis was carried out using ImageJ software (National Institutes of Health). Images shown in the figures correspond to maximum-intensity projections (MIP) of z-stacks (8 steps) acquired with a z-step size of 1 μm.

#### Analysis of Ig secretion

Cells electroporated at D5 with control or CAV1 siRNA were seeded at 5.10^5^ cells/mL, and culture supernatants were collected at D6, D7 and D8. Immunoglobulin secretion was assessed by ELISA, whereas the frequency of antibody-secreting cells was determined by ELISPOT.

##### ELISA

For sandwich ELISA, cells were plated in 96-well Maxisorp plates (Nunc) coated with a goat anti-human Ig (Jackson Immunoresearch Laboratories; #109–005-064, 4.8 μg/mL). After blocking with TBS/Tween/5% BSA, serial dilutions of supernatant in TBS/Tween/1% BSA were added for 2 h at RT. Purified IgG, IgA and IgM (Jackson Immunoresearch Laboratories) were used as standards. Revelation of captured IgG, IgA and IgM was performed using secondary HRP-coupled antibodies specific for γ (#109–035-098), α (#109–035-011) or μ (#109–035-129) chain, respectively (Jackson Immunoresearch Laboratories, 1/15,000 dilution). After washing, plates were developed for 5–10 min with TMB substrate (BD Biosciences, #555214) and then stopped with 0.2 M H_2_SO_4_. Absorbance was measured at 450 nm, with background correction at 570 nm, using a Bio-Rad microplate reader.

##### ELISPOT

For ELISPOT assays, 96-well MultiScreen plates (Millipore; MSIPS4510) were coated overnight with AffiniPure goat anti-human IgM, IgA, and IgG (H + L) antibodies (Jackson ImmunoResearch). After washing with PBS, the plates were blocked with culture medium for 2h at 37°C. After 3 washes with PBS, 2,500 cells per well were seeded and incubated for 24 h at 37°C. Then, plates were washed three times with PBS before incubation with detection antibodies: PE-conjugated goat anti-human IgA (0.625 μg/mL), PE-conjugated goat anti-human IgG (0.3125 μg/mL), and FITC-conjugated goat anti-human IgM (5 μg/mL) (SouthernBiotech). Following the detection step (2h in the dark at room temperature), plates were washed three additional times with PBS, air-dried, and immunospots were enumerated using an ImmunoSpot® S6 Universal Analyzer (CTL, Cleveland, OH, USA).

#### Flow cytometry analyses

Flow cytometry acquisitions were performed on LSR Fortessa X20 (BD Biosciences) and CytoFLEX S (Beckman Coulter) instruments and analyzed using FlowJo Software v10 (FlowJo).

For BCR experiments, NBCs were electroporated at D0 with siCTL or siCAV1 and cultured 24 h with 100 ng/mL CD40L to induce Cav-1 expression. After dead cell removal (Dead Cell Removal kit; Miltenyi Biotec), cells were starved in RPMI medium for 1 h at 37°C and stimulated with F(ab’)_2_ goat anti-human IgM (H + L) (20 μg/mL) for 5 min. Cells were then fixed and permeabilized using the Transcription Buffer Set (BD Biosciences) and stained with phospho-specific antibodies: anti-Syk (pY348)-Alexa Fluor 488 (clone I120-722), BLNK (pY84)-PE (clone J117-1278), PLC-γ2 (pY759)-Alexa Fluor 488 (clone K86–689.37), BTK (pY180)-PE (clone N35-86), ERK1/2 (pT202/pY204)-Alexa Fluor 647 (clone 20A) (all from BD Biosciences), and CD79A (pY182)-Alexa Fluor 647 (clone D1B9; Cell Signaling Technology).

For autophagy assessment, cells were first stained with anti-CD38–APC (clone HIT2; BD Biosciences), followed by the CYTO-ID Autophagy Detection Kit 2.0 (Enzo Life Sciences) according to the manufacturer’s instructions.

For *in vitro* assessment of B:T conjugates formation, NBCs were electroporated at D0 with siCTL or siCAV1 and stimulated with F(ab’)_2_ goat anti-human IgM (H + L) (2.4 μg/mL) for 24 h, then stained with VPD450 (BD Biosciences). Cells were subsequently loaded for 1 h at 37°C with superantigens (SAg): Toxin Shock Syndrom Toxin-1, Staphylococcus Enterotoxin A and Staphylococcus Enterotoxin B (2 μg/mL each; Toxin Technology). Allogeneic blood CD4^+^ T cells were isolated using the CD4^+^ T cell Isolation (Miltenyi Biotec) and cultured for at least 2 days in the presence of anti-CD3/anti-CD28 (0.2 μg/mL each; Sanquin) and IL-2 (100 U/mL). Activated T cells were labeled with CFSE (e-Bioscience) and co-cultured with B cells at a 1:1 ratio in round-bottom 96-well plates. At the indicated time points, cells were gently harvested and analyzed by flow cytometry.

### Quantification and statistical analysis

Data are presented as mean ± SD or median with range as indicated.

For comparisons between two groups, the Wilcoxon signed-rank test (paired) or Mann–Whitney U test (unpaired) was used. For multiple comparisons, Friedman (paired) or Kruskal–Wallis (unpaired) tests with Dunn’s post hoc correction were applied.

One-way or two-way ANOVA was used for parametric data.

Pearson’s correlation coefficient (r) was calculated for correlation analyses.

All tests were two-tailed and performed in GraphPad Prism v10, with *p* < 0.05 considered significant.

“n” values represent the number of independent donors and experiments. *p* ≤ 0.05 (∗), *p* ≤ 0.01 (∗∗); *p* ≤ 0.001 (∗∗∗); *p* ≤ 0.0001 (∗∗∗∗).

For the comparative kinetic analysis of early B-cell activation depicted in Figure 5G, data were collected from independent biological replicates (*n* = 3 to *n* = 4 human donors per subgroup) across multiple longitudinal time points (6h, 10h, 24h, and 34h). Due to the expansive number of experimental conditions and the finite yield of primary human naive B cells isolated per donor, not all activation cocktails could be evaluated concurrently for every donor. This logistics constraint resulted in an unbalanced experimental design with missing observations in specific subsets.

To rigorously model this architecture without losing statistical power or introducing bias, data were analyzed using a Linear Mixed-Effects Model fit via Restricted Maximum Likelihood (REML) estimation, followed by Tukey’s post-hoc test for multiple pairwise comparisons. The biological donor was specified as a random effect to account for the nested, paired nature of the longitudinal measurements and to isolate baseline inter-donor variations. Treating the data matrix within a mixed-effects framework effectively accommodates the missing replicates, providing robust *p*-value estimations despite the unbalanced design. Individual biological data points are explicitly displayed on all plots to ensure full transparency regarding primary human tissue variability. All statistical computations were performed using R.4.5.0 package lme4.

## Supplemental material

Document S1. Figures S1–S7 and Data S1

Table S1. TRRUST Transcription Factors and MSigdDB Hallmark signatures enrichment in B cell differentiation clusters, related to Figures 1 and 2

Table S2. List of genes composing GC B cells signatures and published signatures used for the scRNA-seq analysis, related to Figure 2

Table S3. Top transcription factors and gene signatures enrichment in the six subclusters of activated B cells, related to Figure 2

Table S4. List of genes associated to hdWGCNA modules (M1 to M13), related to Figure 3

Table S5. List of differentially expressed genes between siCAV1 and siCTL-D7 plasmablasts, related to Figure 6

Table S6. List of proteins interacting with Cav-1 obtained by affinity purification - mass spectrometry (LFQ intensities), related to Figure 7 XBP1 targets present in module M3 are highlighted in yellow.

Table S7. List of TaqMan Gene Expression Assays (FAM)
