# Supplementary material for: Single-cell mapping of human plasma cell fate reveals a commitment checkpoint and Cav-1β expression associated with mTORC1 machinery
Source: iScience. 2026 Oct 13;29(11):117865. doi: 10.1016/j.isci.2026.117865 (PMC13682384; doi:10.1016/j.isci.2026.117865)
Supplement: Supplementary file 1 [file mmc1.pdf]

iScience, Volume *29*

Supplemental information

Single-cell mapping of human plasma cell fate reveals a commitment checkpoint and Cav-1β expression associated with mTORC1 machinery

Gersende Caron, Fabienne Desmots, Mélanie Chabaud, Maud Lemarié, Vincent Olivier, Fabrice Chatonnet, Céline Nonn, Cassandra Jadeau, Simon Léonard, Céline Monvoisin, Kathleen Santamaria, Cédric M. Blouin, Noudjoud Attaf, Tarek Gharsalli, Pierre Milpied, Christophe Lamaze, Céline Pangault, Marion Haas, and Thierry Fest

Figure S1. Quality control and validation of in vitro human tonsillar B cell differentiation, related to Figure 1.

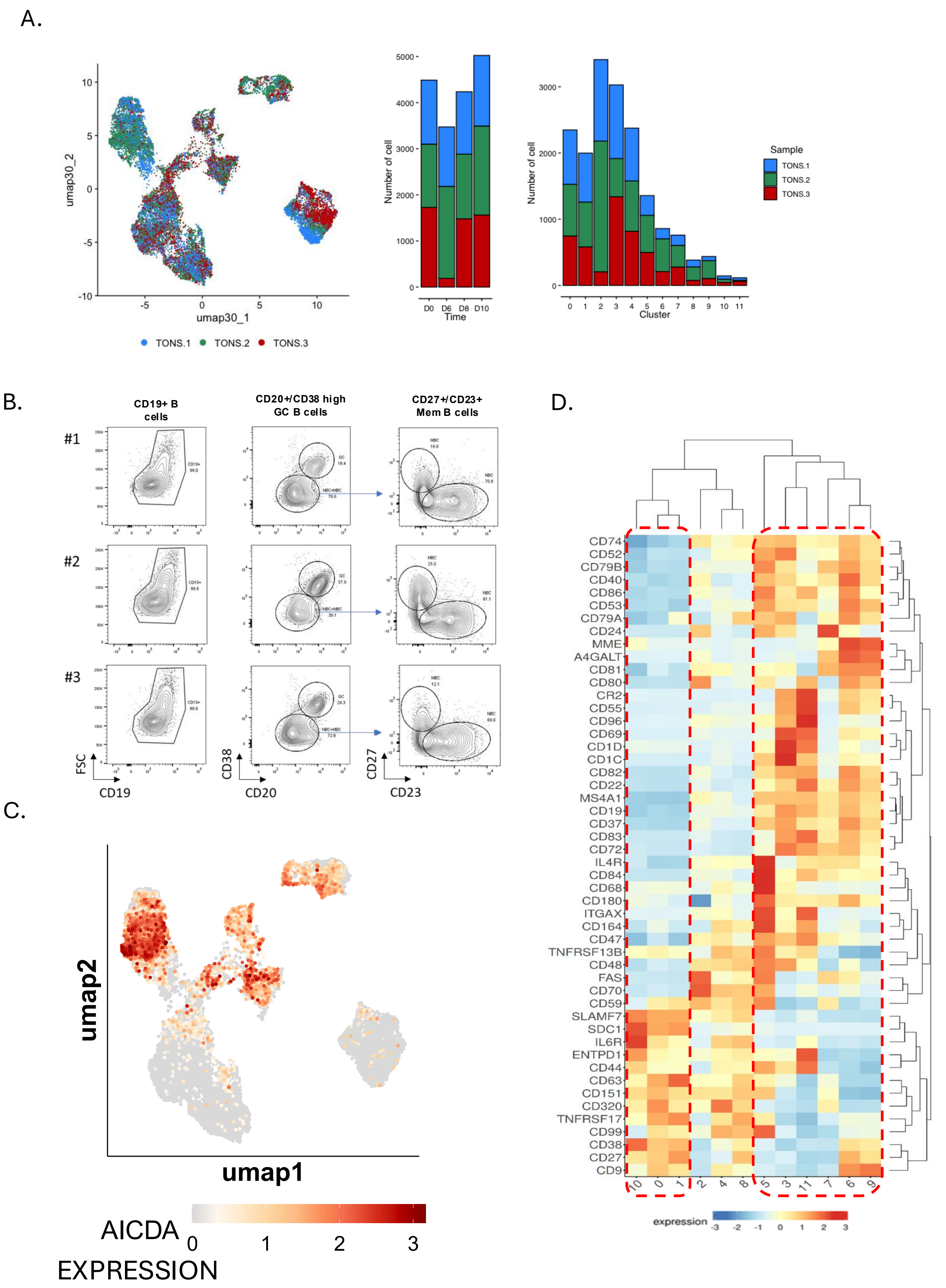

A) UMAP plot showing the distribution of cells derived from three independent tonsil donors across time points and clusters (TONS, tonsil).

B) Flow cytometry profiles of day 0 CD19-positive B cells, determined in the lymphoid window, from the three tonsil samples (GC, germinal center; NBC, naive B cells; MBC, memory B cells).

C) Expression of *AICDA* during B cell differentiation, highlighting transcriptional activity associated with B cell activation.

D) Heatmap of 50 cell surface marker genes across clusters. Color intensity indicates relative expression per cluster.

Figure S2. Transcriptional characterization of naive, memory, and activated B cell states, relative to Figure 2.

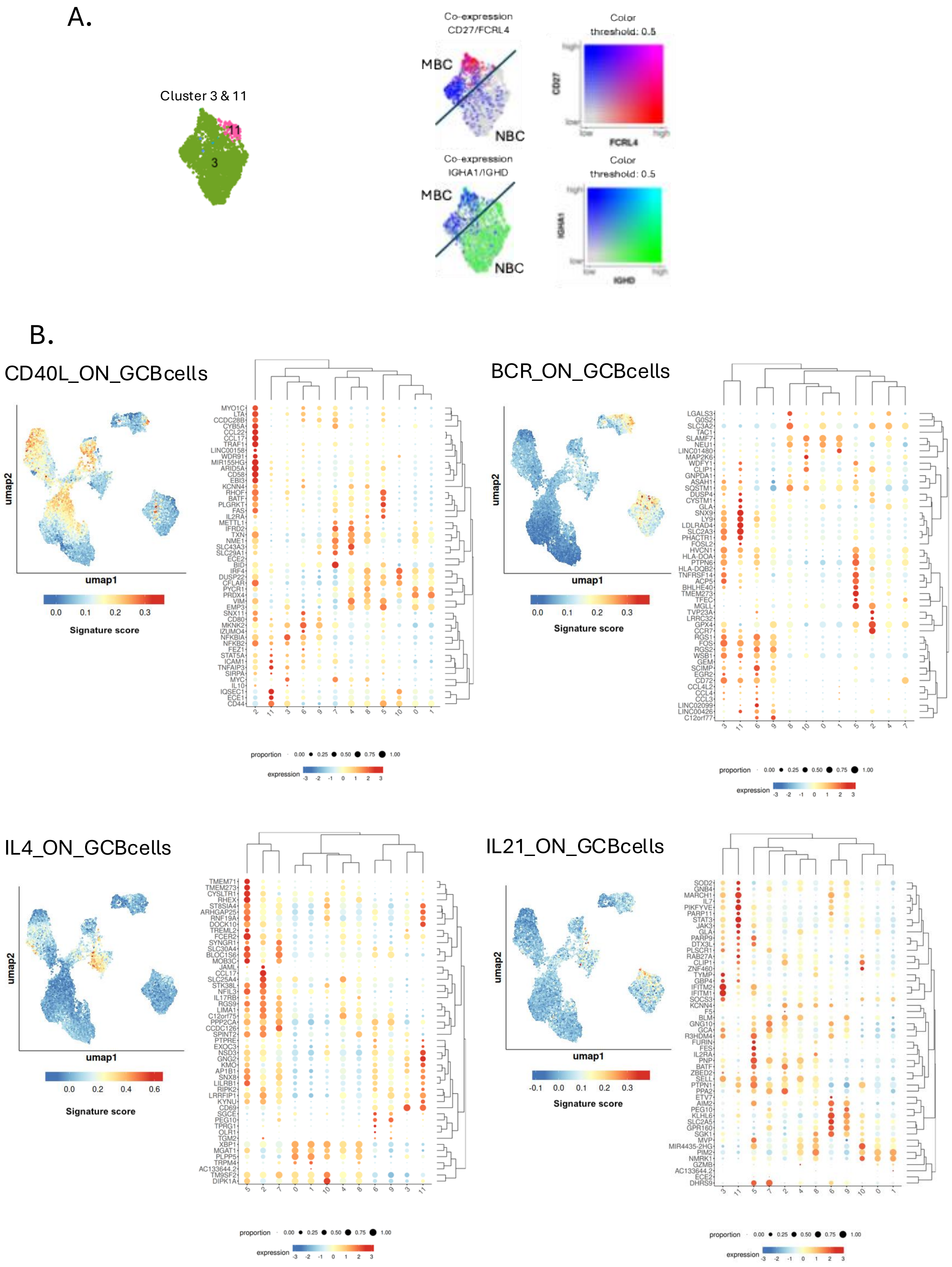

C.

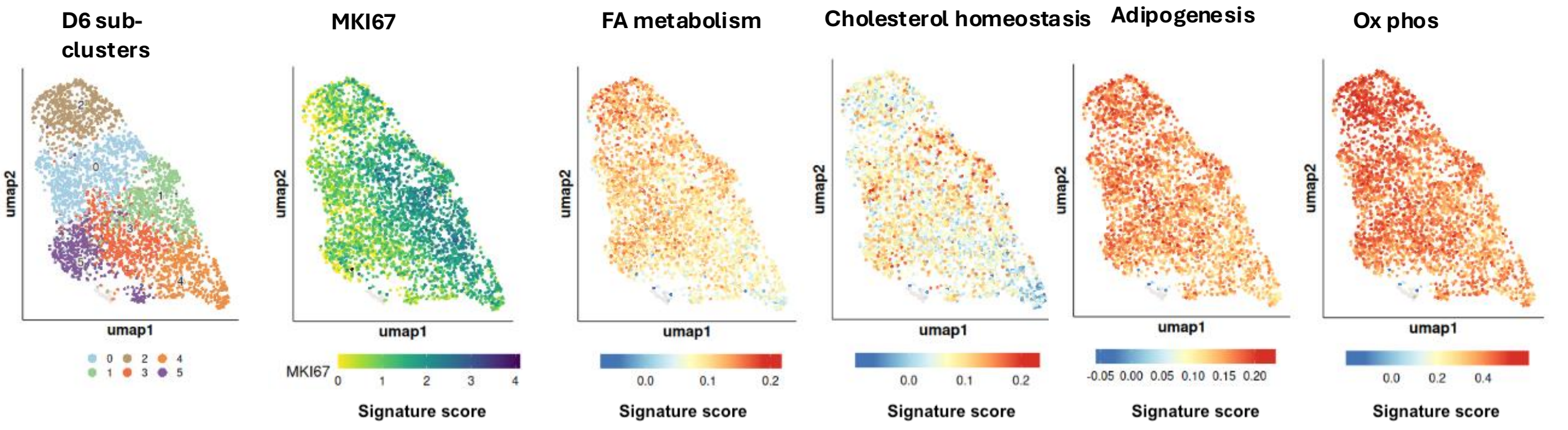

D.

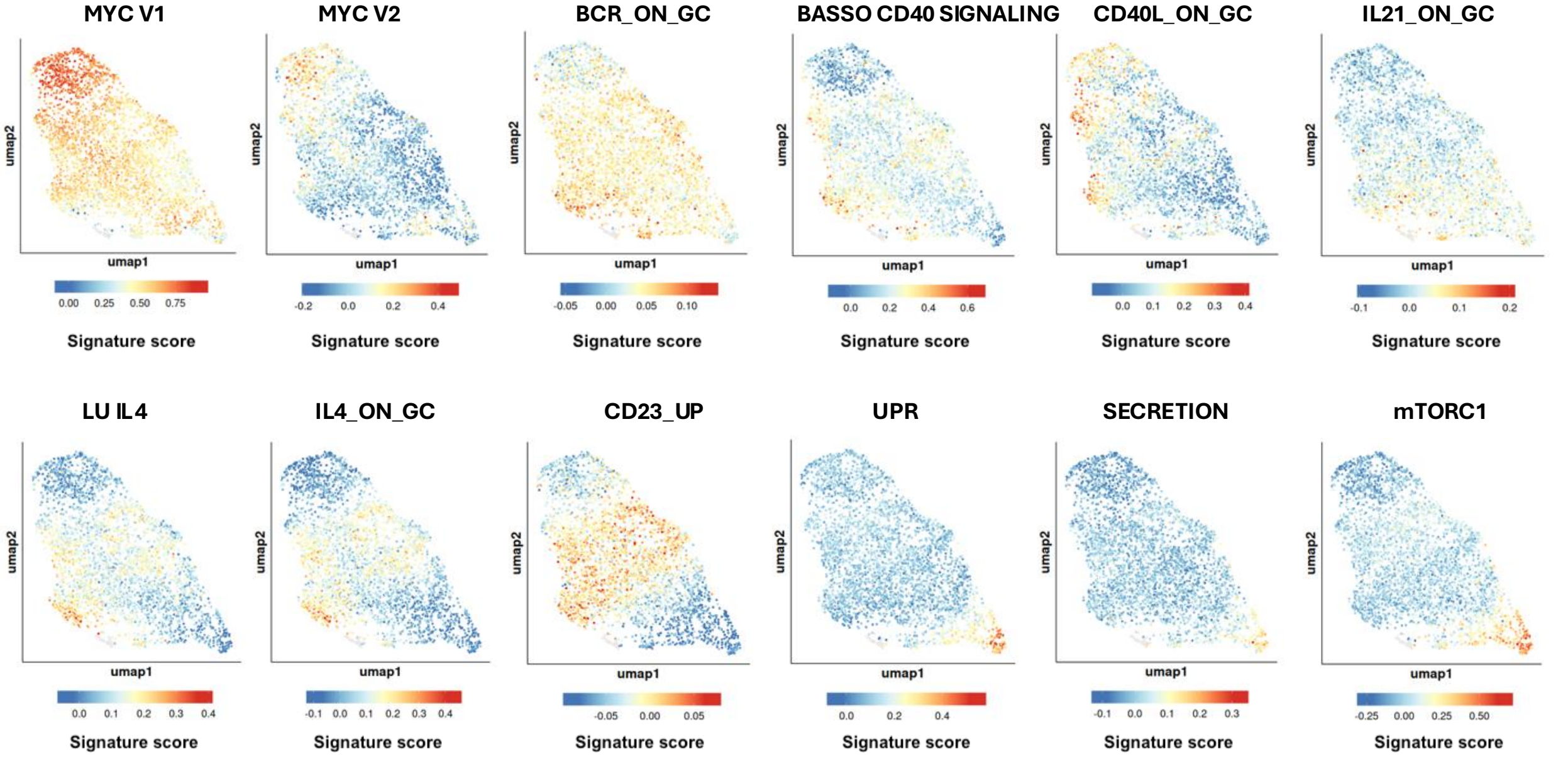

A) UMAP plots of day 0 clusters 3 and 11 highlighting IgD^{+} naïve B cells (NBC) and class-switched CD27^{+} and FCRL4^{+} memory B cells (MBC).

B) Bubble plots showing expression of genes from the CD40L_, BCR_, IL4_, and IL21_ ON_GCBcells signatures across clusters. The 50 most highly expressed genes from each gene set are displayed

C) UMAP plots of activated B cells (cluster 2) showing high MKI67 expression and enrichment of MSigDB Hallmark signatures associated with cellular metabolism in all subclusters.

D) UMAP plots illustrating enrichment of indicated molecular signatures in activated B cell subclusters.

Figure S3. Extended pseudotime and hdWGCNA analyses, related to Figure 3.

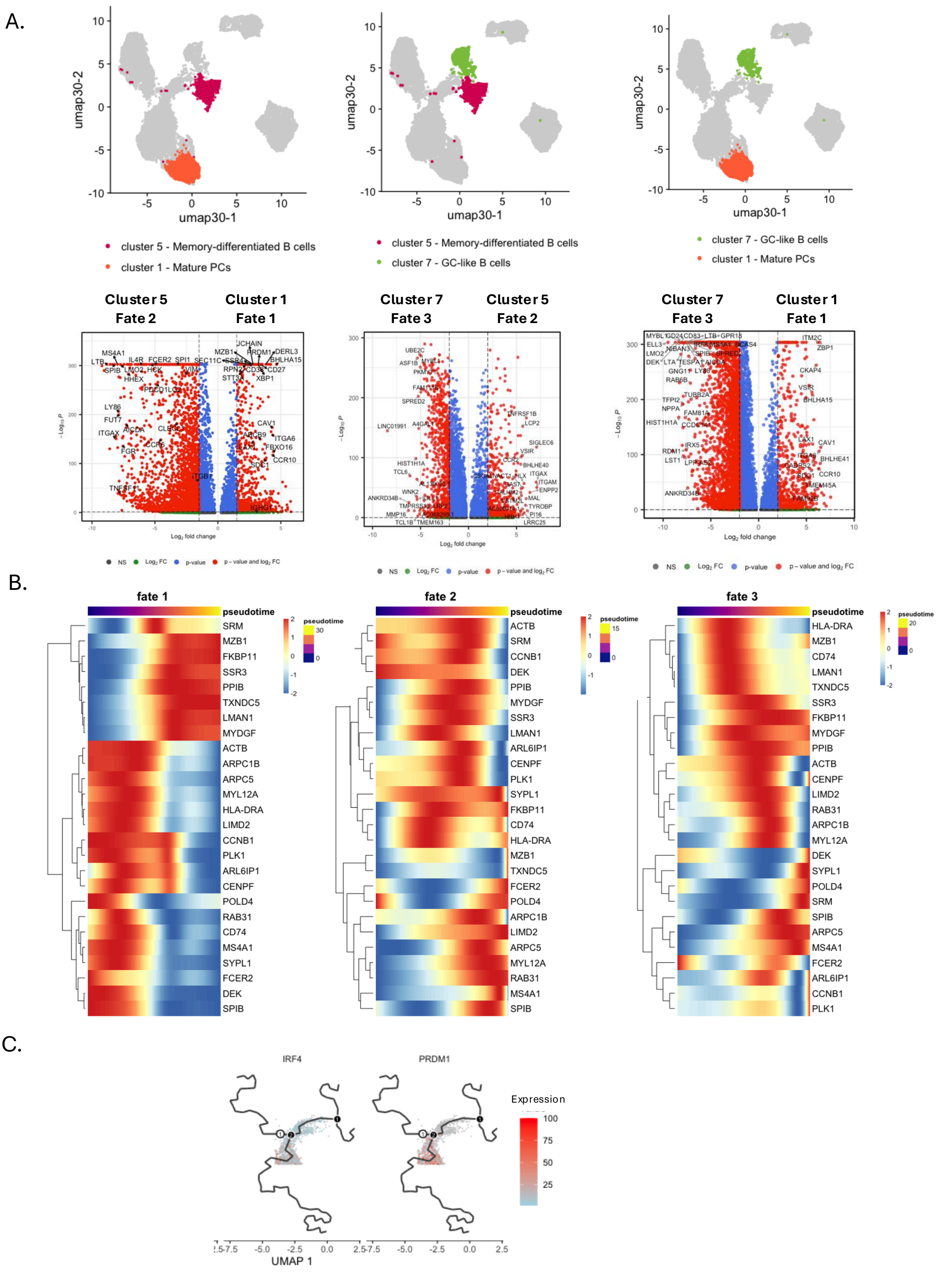

Supplemental Figure 3 (cont'd)

D.

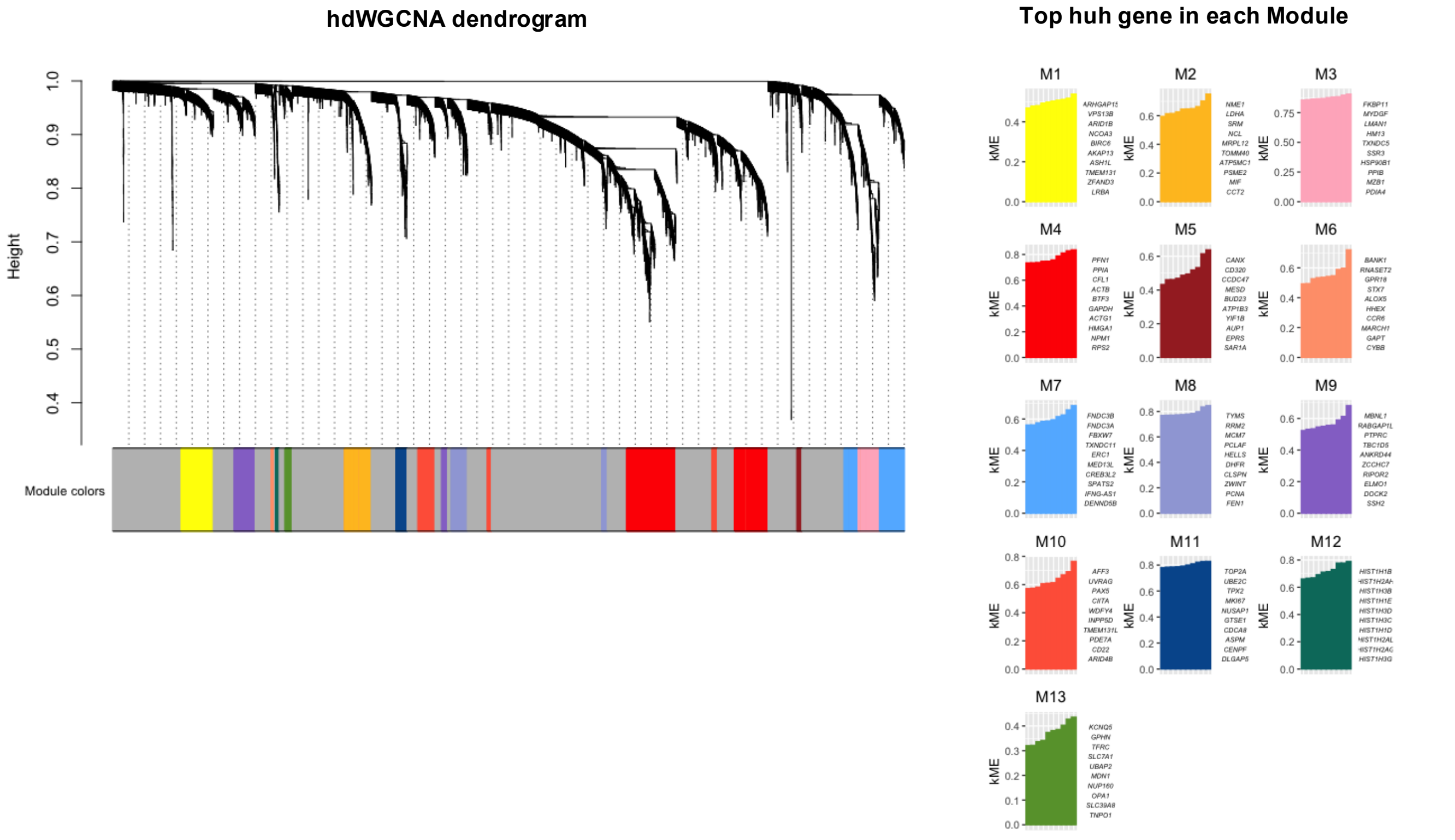

E.

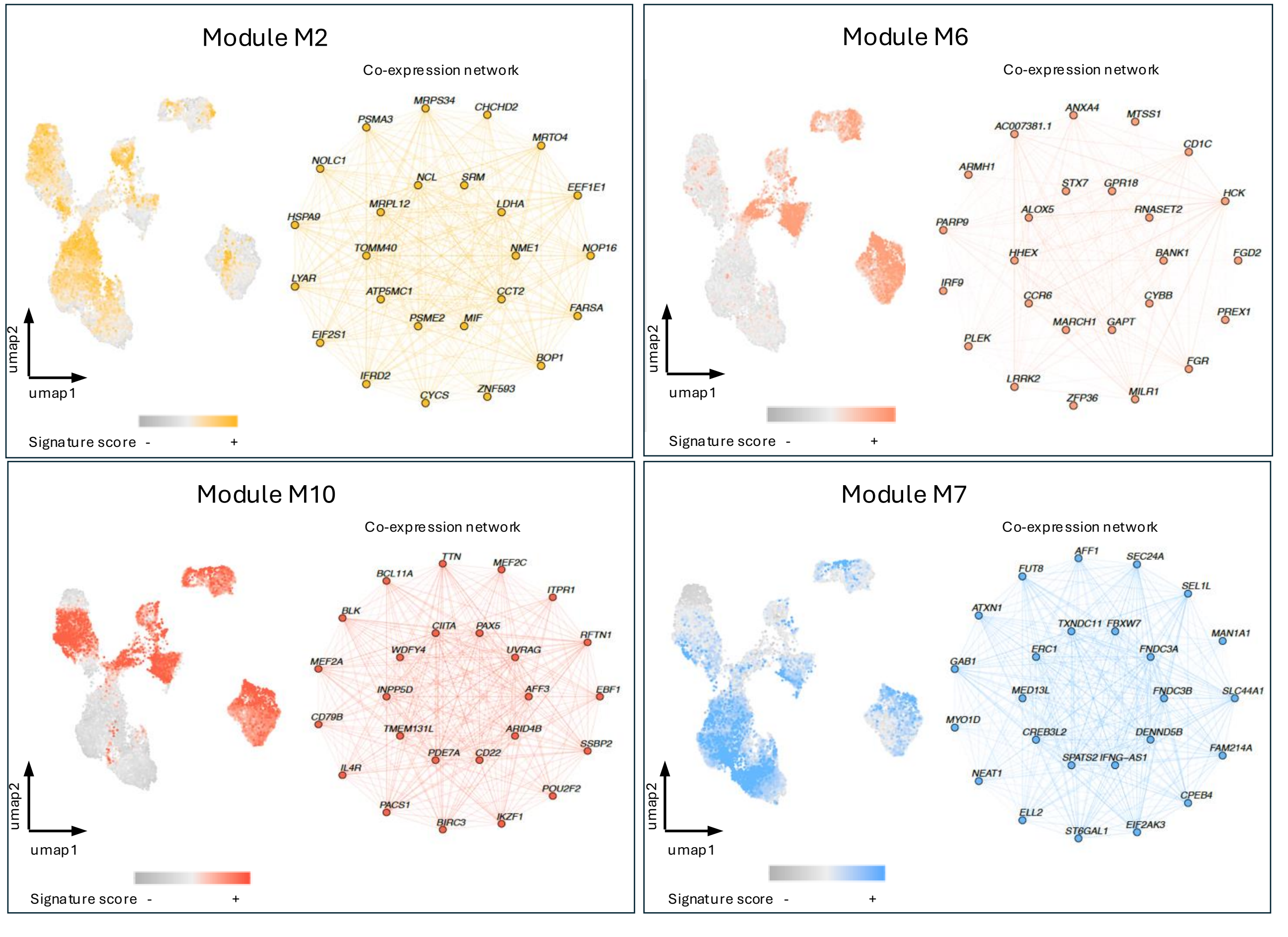

Supplemental Figure 3 (cont'd)

F.

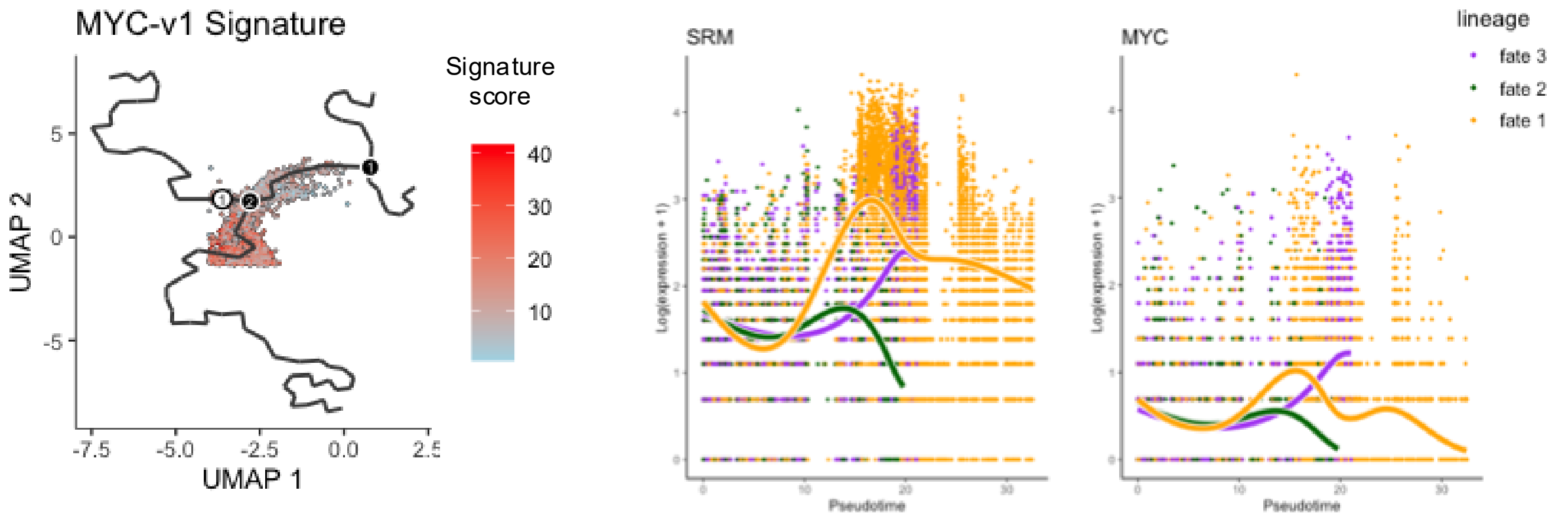

G.

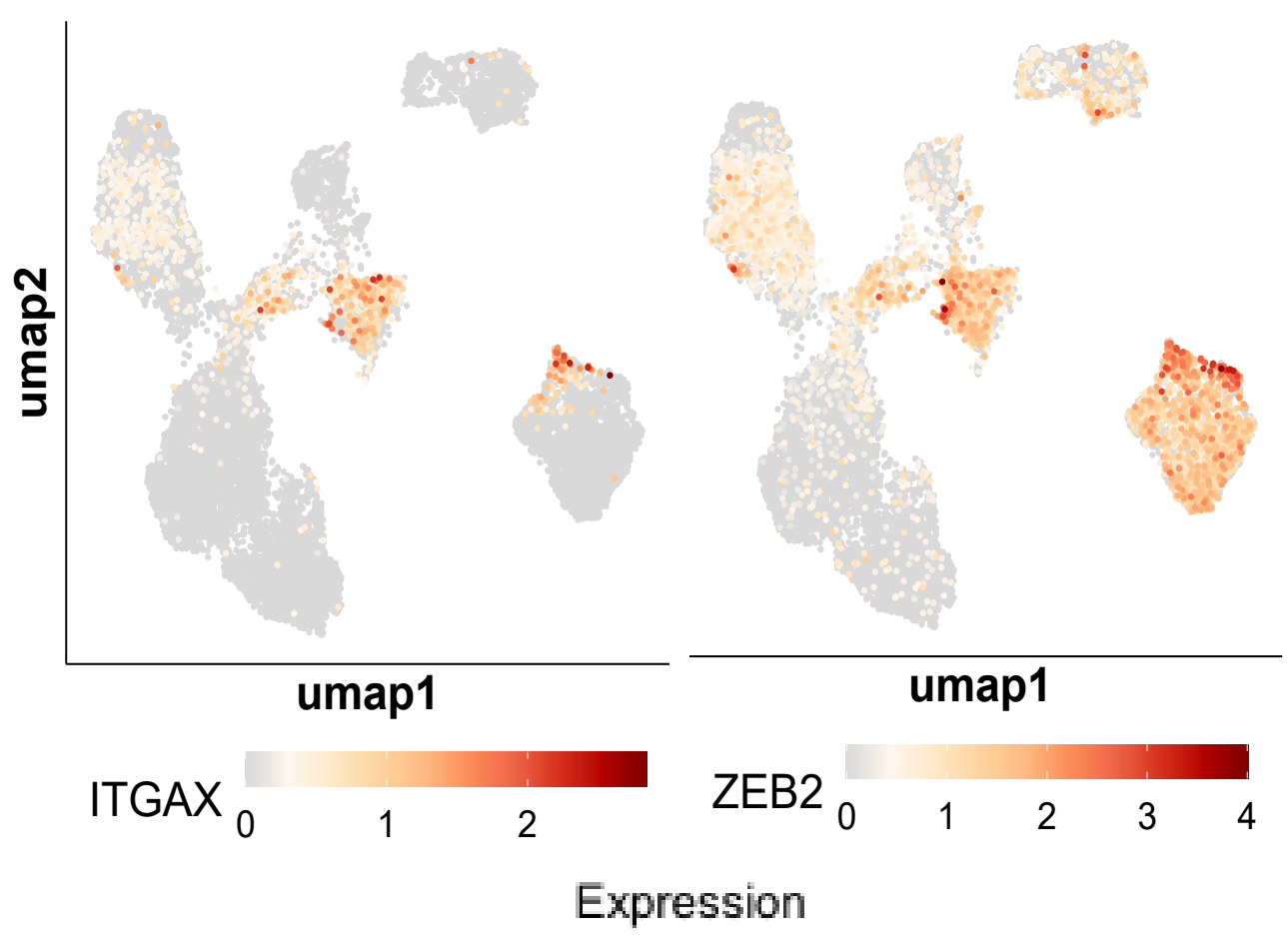

Supplemental Figure 3 (cont'd)

H.

Cluster	Score	Category	Term (ID)	Genes in list	Genes in term	% of list	p-value	Genes (XBP1 targets in M3)
1	15.53	Cellular Component	Membrane (KW-0472)	111	72	7.2%	1.7×10^{-11}	DPAGT1,CHPF,KDELR1,SLC35B1,CISD2,TXNDC15,LAPTM4A,KRTCAP2,CLCC1,SLC39A7,SEC62,CCDC163,SEC63,ALG5,ALG2,ALG14,MOGS,PDIA6,ARMCX3,PLPP5,SELENOS,KDELR2,PREB,COPB2,LRRC59,FKBP2,TMED10,RPN2,FICD,UBA5,RPN1,SLC38A10,SLC1A4,DNAJC25,UBE2J1,MTDH,MLEC,SARAF,NDUFV3,SEC31A,NUS1,XBP1,CAV1,SURF4,TVP23B,OSTC,DNAJC10,CALU,SRPRA,STT3A,SRPRB,STT3B,ACBD3,COPE,FKBP11,ARF4,KCNK6,B4GALT3,TRAM1,HERPUD1,UFL1,SEC61A1,SERP1,MAN1A2,STBD1,TMED2,TMED9,SEC13,SSR4,SSR2,SSR3,WIPI1,ARFGAP3,BET1L,DDOST,CREB3,DNAJC1,TMEM214,DAD1,GORASP2,B4GALT7,HM13,GOSR2,DERL3,USO1,DERL1,DERL2,SELENOK,TM9SF3,LMAN1,MSTO1,SLC17A9,LMAN2,TMEM208,MGAT1,MGAT2,RABAC1,SEC11C,YIPF5,SYVN1,LYSMD3,AIG1,TM9SF1,SPCS3,SPCS2,COPG1,P4HB,SEC24D,EBAG9,NUCB2,BET1
2	14.80	Cellular Component	Golgi apparatus (KW-0333)	151	38	24.6%	8.1×10^{-7}	ARF4,COPB2,B4GALT3,TMED10,CHPF,KDELR1,UBA5,GOSR2,USO1,MYDGF,LMAN1,PRRC1,MAN1A2,LMAN2,TMED2,MGAT1,MGAT2,SLC39A7,RABAC1,TMED9,CAV1,YIPF5,SURF4,WIPI1,LYSMD3,ARFGAP3,BET1L,CREB3,GORASP2,CALU,KDELR2,COPG1,ACBD3,EBAG9,B4GALT7,COPE,NUCB2,BET1
3	12.48	Biological Process	Transport (KW-0813)	88	21	26.6%	3.5×10^{-9}	KCNK6,ARF4,COPB2,TRAM1,TMED10,KDELR1,GOSR2,SLC35B1,USO1,CAV1,DERL1,HDLBP,SLC38A10,SLC1A4,SELENOK,LMAN1,SEC61A1,LAPTM4A,SERP1,SLC17A9,LMAN2,TMED2,SARAF,SLC39A7,CLCC1,NDUFV3,SEC62,SEC31A,SEC63,XBP1,TMED9,SEC13,YIPF5,SURF4,ARFGAP3,BET1L,PREB,KDELR2,COPG1,SEC24D,COPE,BET1
4	8.81	GO: Cellular Component	Focal adhesion (GO:0005925)	146	8	5.3%	0.009	PDIA3,HSPA5,CAV1,HYOU1,P4HB,CALR,PPIB,HSP90B1
5	4.69	GO: Molecular Function	ATPase binding (GO:0051117)	148	3	2.0%	0.004	SDF2L1,SELENOS,CAV1,DNAJC10,DERL1,SYVN1

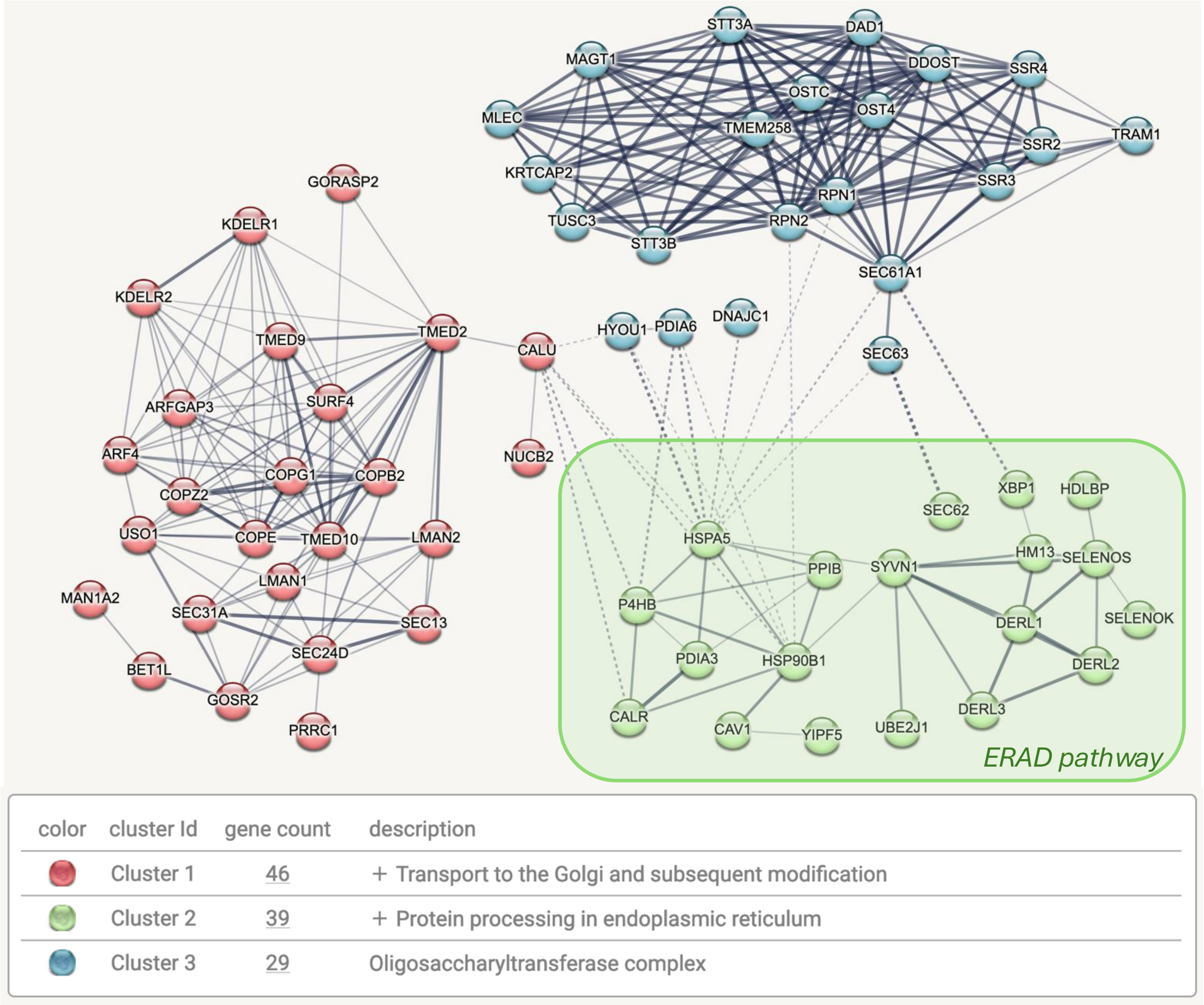

Supplemental Figure 3 (cont'd)

I.

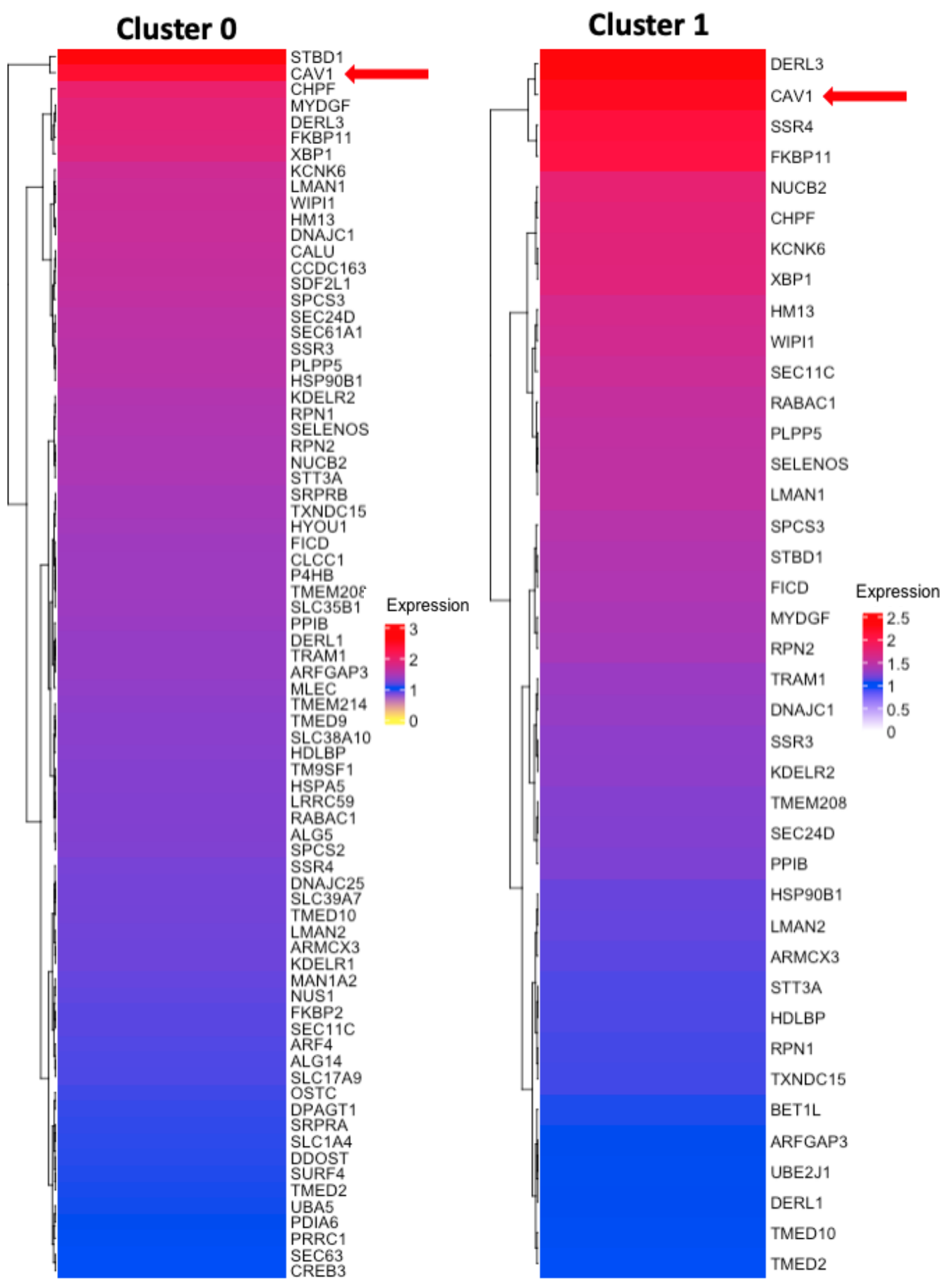

j.

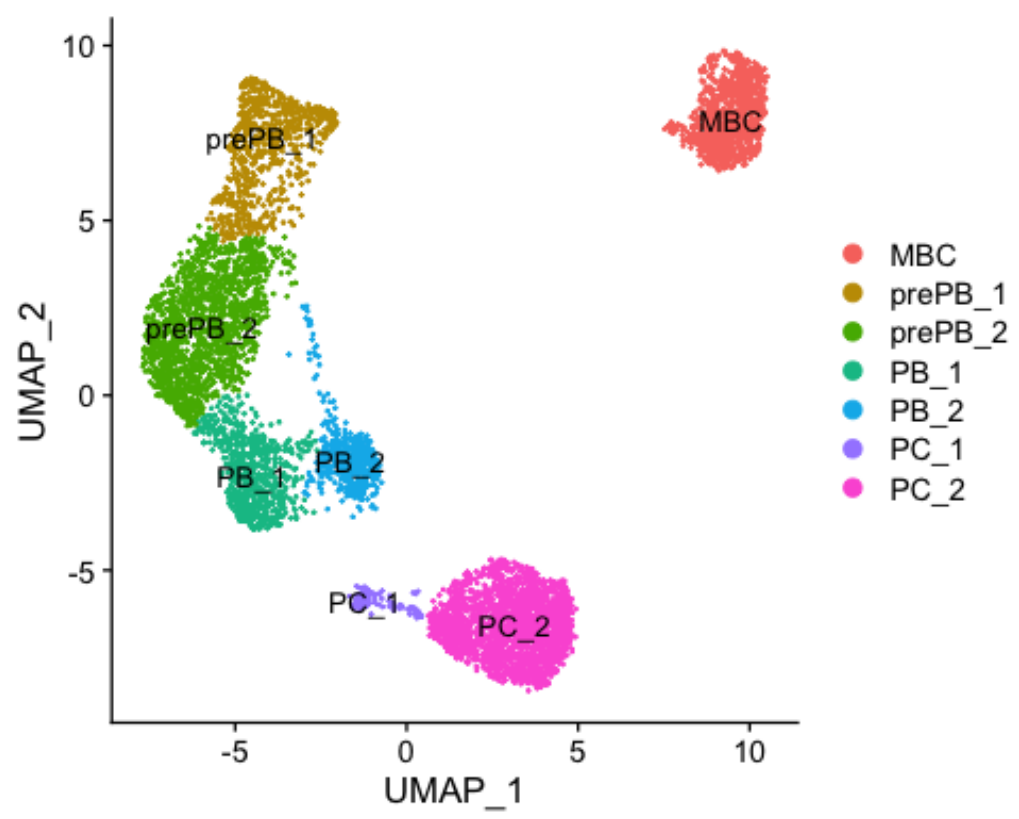

K.

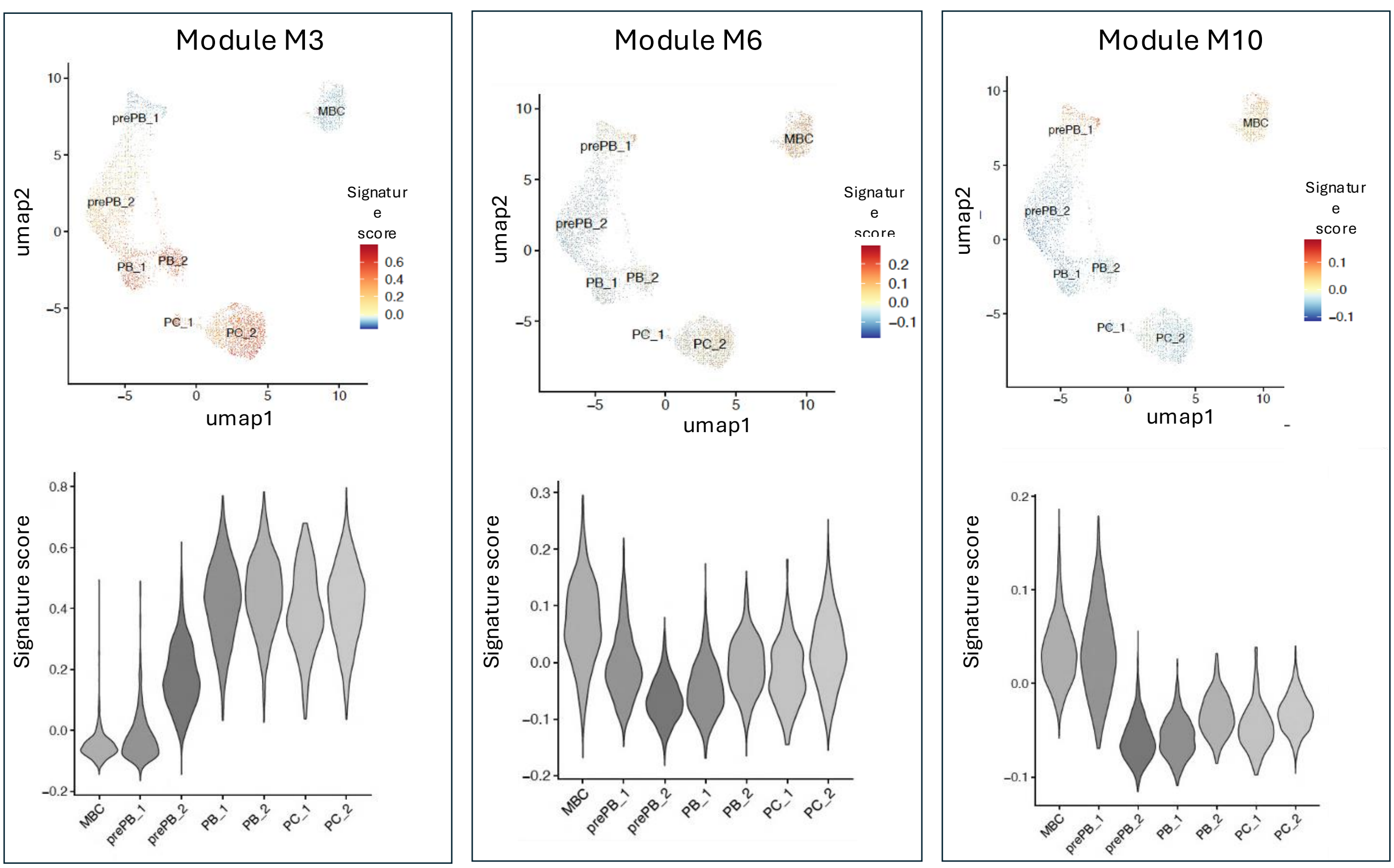

A) UMAP showing differentiation trajectories. Volcano plots depict differential gene expression between clusters defining fate decisions (Cluster 5 vs. Cluster 1; Cluster 7 vs. Cluster 5; Cluster 7 vs. Cluster 1).

B) Heatmaps of representative genes for each fate (Fates 1–3), illustrating dynamic expression along pseudotime.

C) Pseudotime trajectories highlighting IRF4 and PRDM1 expression.

D) hdWGCNA module identification. Left: Hierarchical clustering dendrogram of metacells with color-coded module assignments. Right: Bar plots representing the top 10 hub genes for each module (M1–M13), ranked by their network connectivity.

E) UMAP visualization of representative gene modules (M2, M6, M10, M7) with corresponding co-expression networks with top-hub genes.

F) MYC-V1 signature activity projected on UMAP (left) and along pseudotime (right) for representative SRM and MYC genes; colored trajectories denote distinct lineages.

G) UMAP of ITGAX and ZEB2 expression.

H) ChEA3 analysis of M3 module genes identifies XBP1 as enriched (121/294 genes). DAVID functional annotation reveals enrichment in membranes, Golgi, transport, and ATPase binding. STRING clustering groups XBP1 targets; CAV1 is within cluster 3 (ERAD pathway).

I) CAV1 expression in clusters 0–1 compared with other XBP1-enriched M3 genes ($p < 0.001$; $FC > 2$).

J) UMAP of differentiation stages from an independent in vitro B-to-PC scRNA-seq dataset (PMID: 38643512).

K) UMAP and violin-plot representation of top genes expression from M3, M6, and M10 in the independent dataset; color in the UMAP indicates module enrichment score.

Figure S4. Identification and validation of XBP1-binding motifs in *CAV1*, related to Figure 4

A.

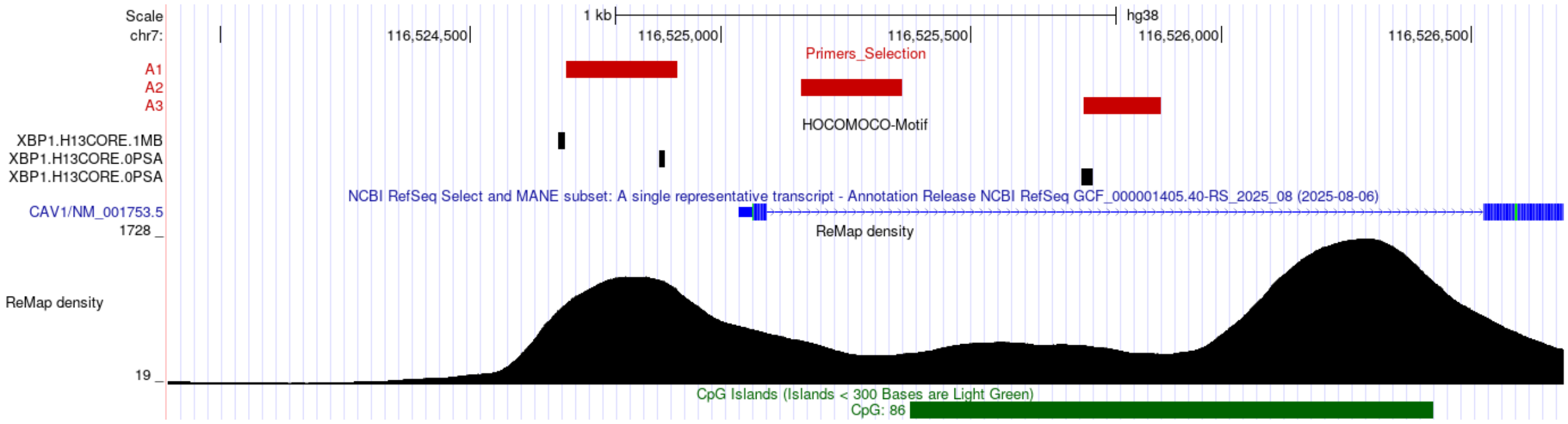

B.

Motif	Motif Logo	Gene (Human)	Quality	Motif Subtype	Data sources
XBP1.H13CORE.0.PS.A	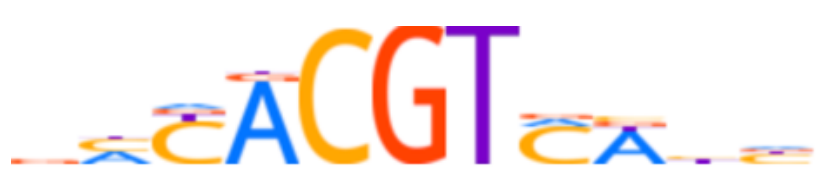	XBP1	A	0	P+S
XBP1.H13CORE.1.M.B	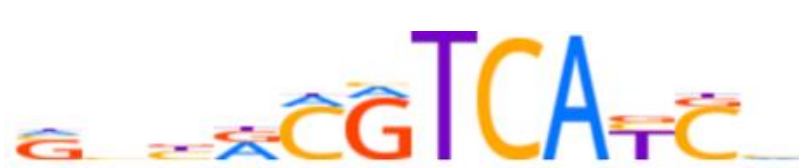	XBP1	B	1	M

C.

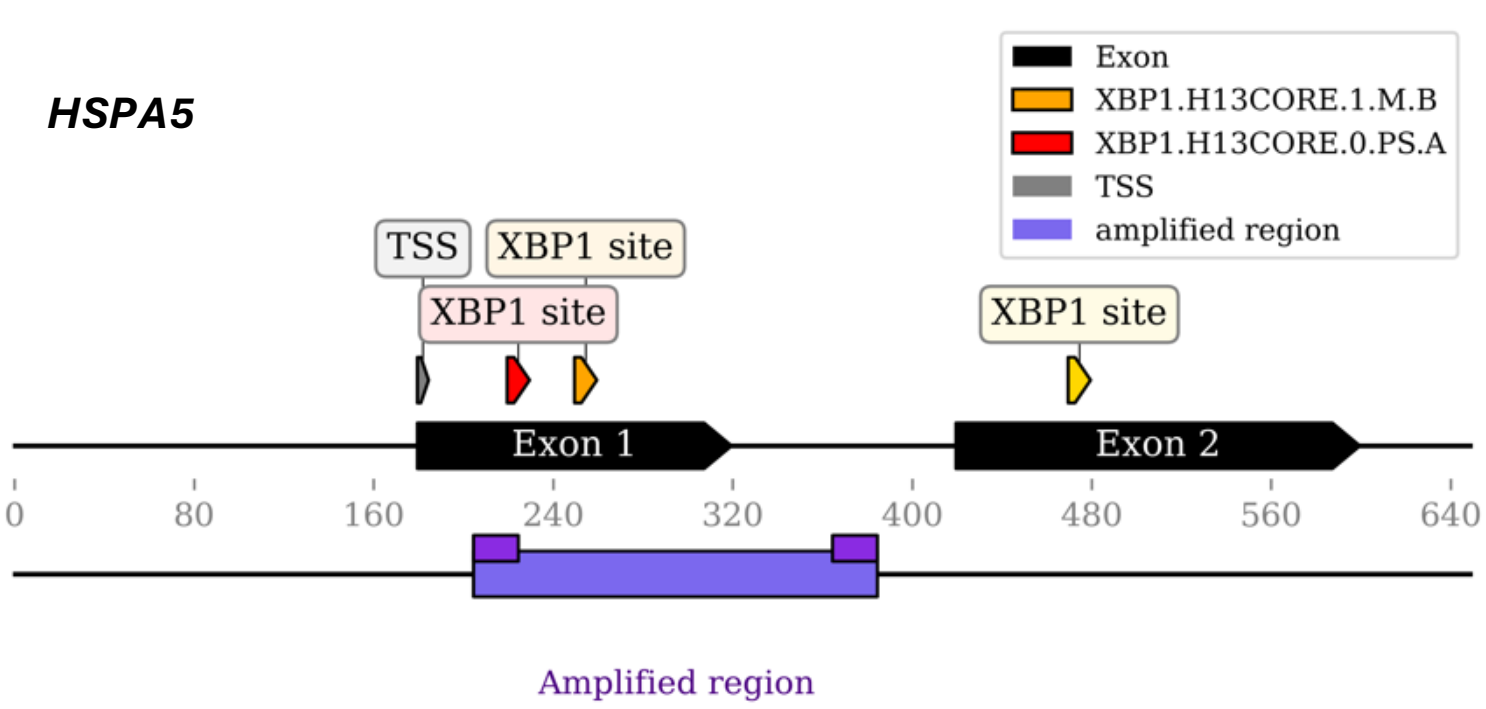

D.

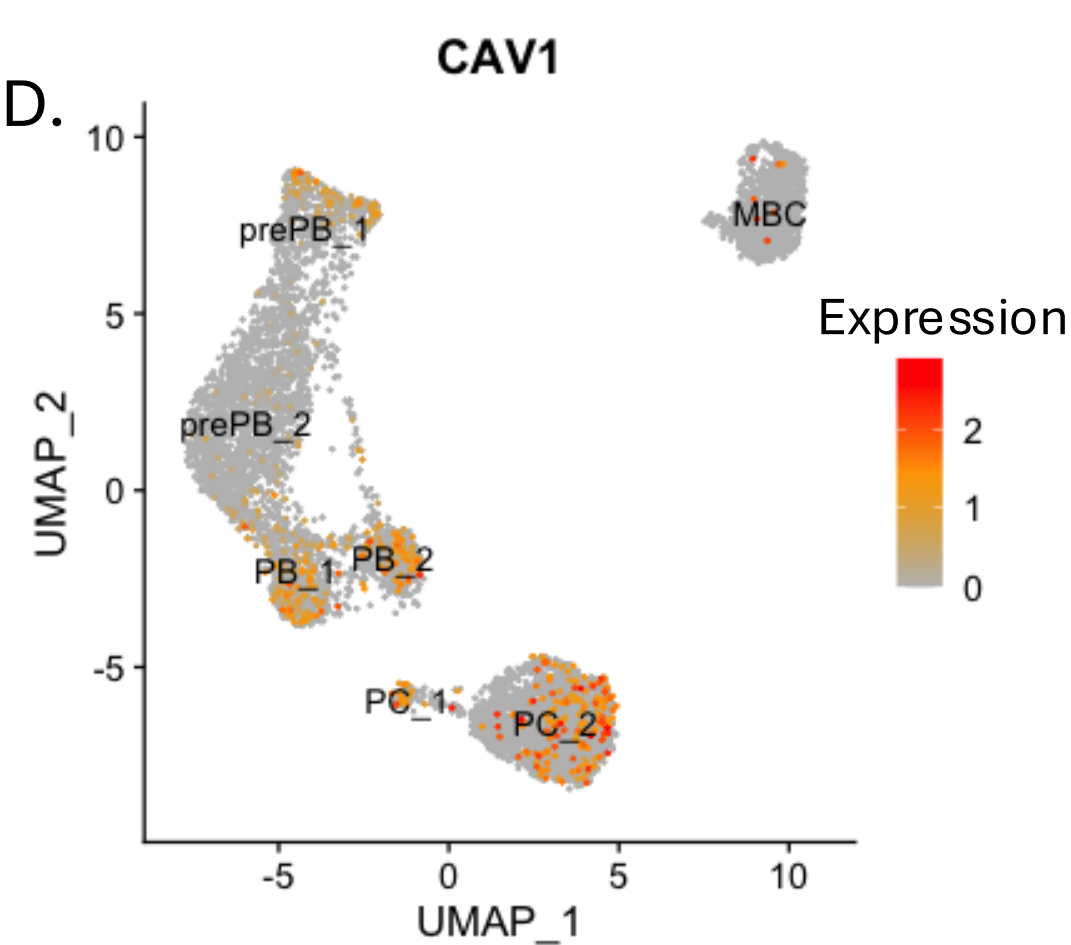

A) UCSC Genome Browser view of CAV1 locus (chr7, hg38) showing primer positions (red), TF motifs (black), RefSeq transcripts (blue), ReMap ChIP-seq density (black), and CpG islands (green).

B) XBP1 motif identification (H13CORE.1.M.B and H13CORE.0.PS) using HOCOMOCO v13. Quality scores: A = ChIP-seq + HT-SELEX, B = one method. Sources: P = ChIP-seq, S = HT-SELEX, M = Methyl-HT-SELEX. “0-rank” indicates most general variant.

C) Schematic HSPA5 locus (−200 bp to +500 bp relative to the TSS, Exons 1–2) showing XBP1.H13CORE consensus site.

D) UMAP of z-scored CAV1 expression across differentiation stages in independent in vitro plasma cell dataset.

Figure S5. Biphasic Cav-1 expression marks early activation and plasma cell differentiation in human B cells, related to Figure 5.

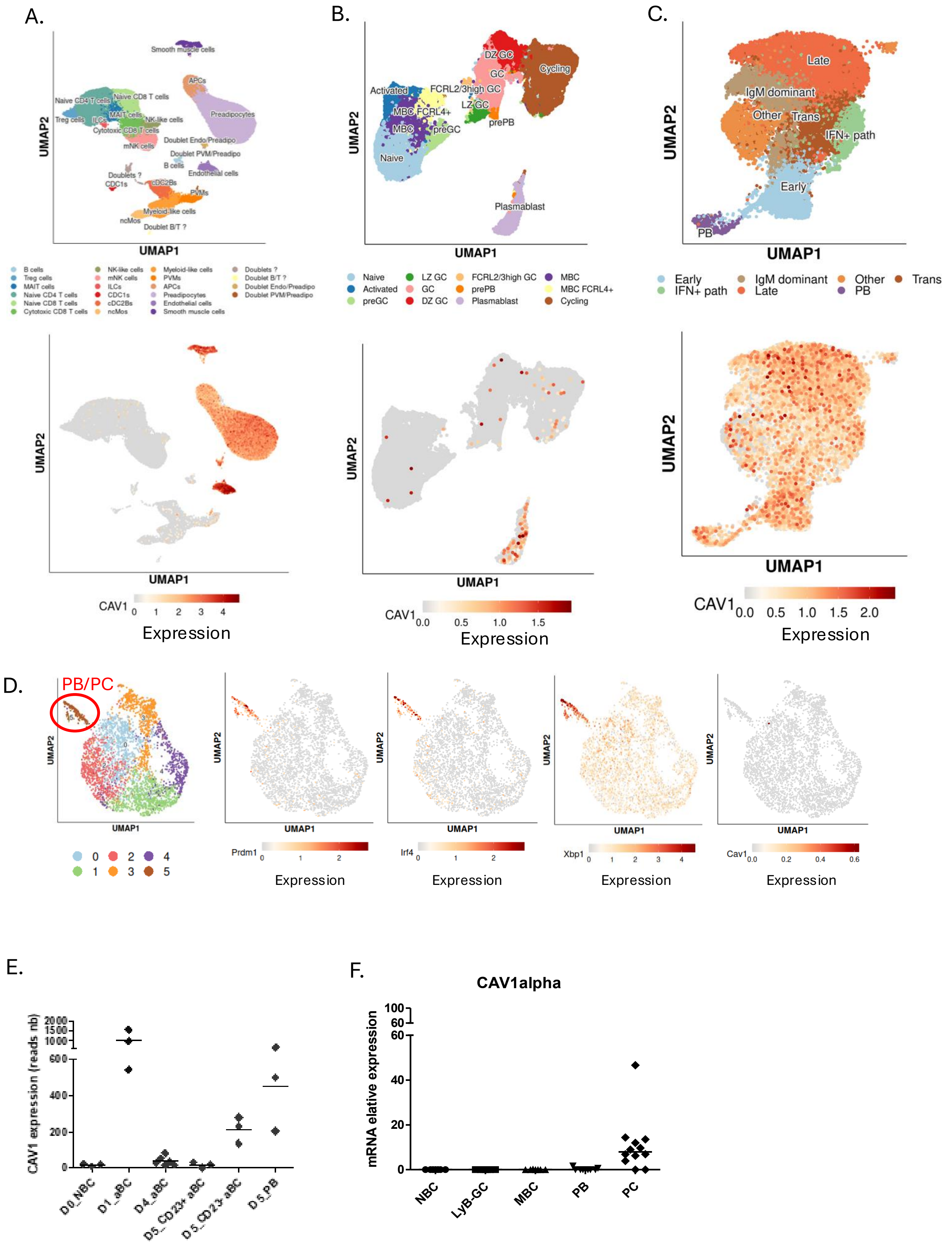

Supplemental Figure 5 (cont'd)

G.

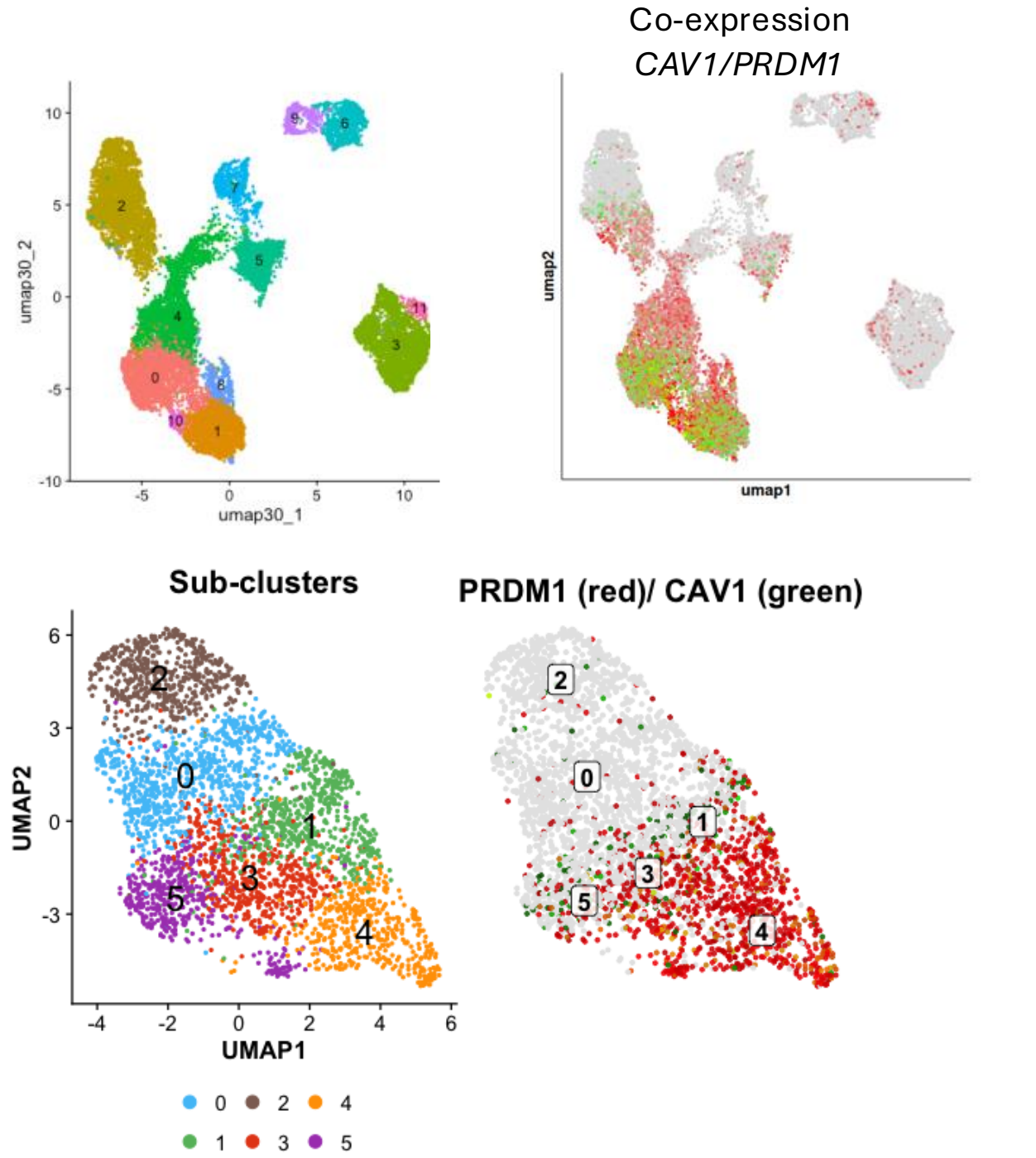

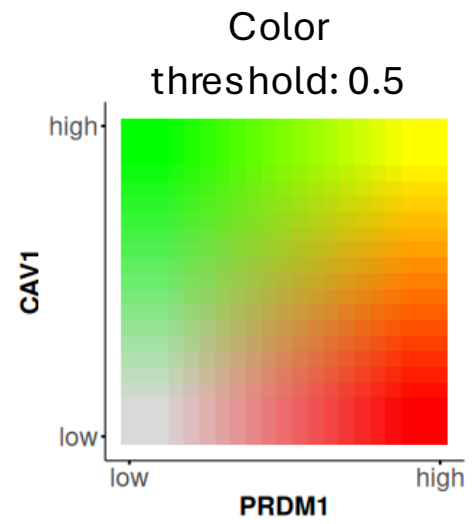

	expression>0	nCells	Percent
**Cluster 5** Memory-differentiated cells	both	12	0.88
	PRDM1	232	17.1
	CAV1	18	1.33
	None	1095	80.39
**SubCluster 4** Pre-plasmablasts	both	81	17.72
	PRDM1	304	66.52
	CAV1	8	1.75
	None	64	14
**SubClusters 1-3-5** CD40L & cytokines signaling	both	95	6.18
	PRDM1	506	32.9
	CAV1	112	7.28
	None	825	53.64
**SubClusters 0-2** Myc & BCR signaling	both	1	0.07
	PRDM1	14	1.02
	CAV1	39	2.83
	None	1325	96.08

H.

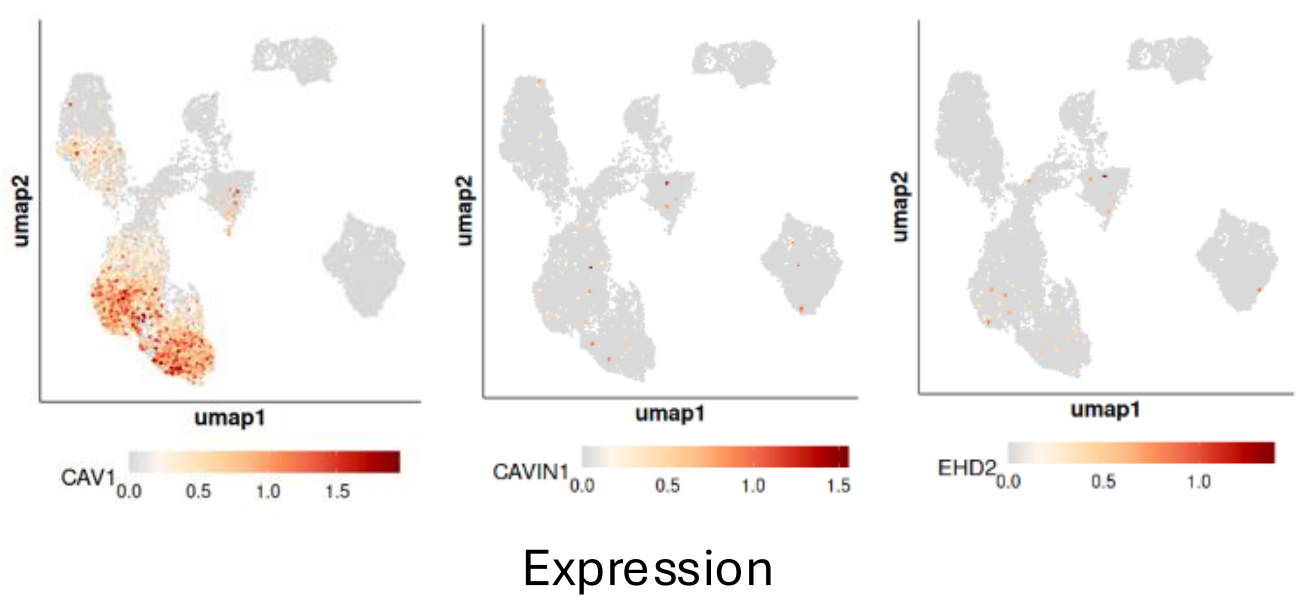

I.

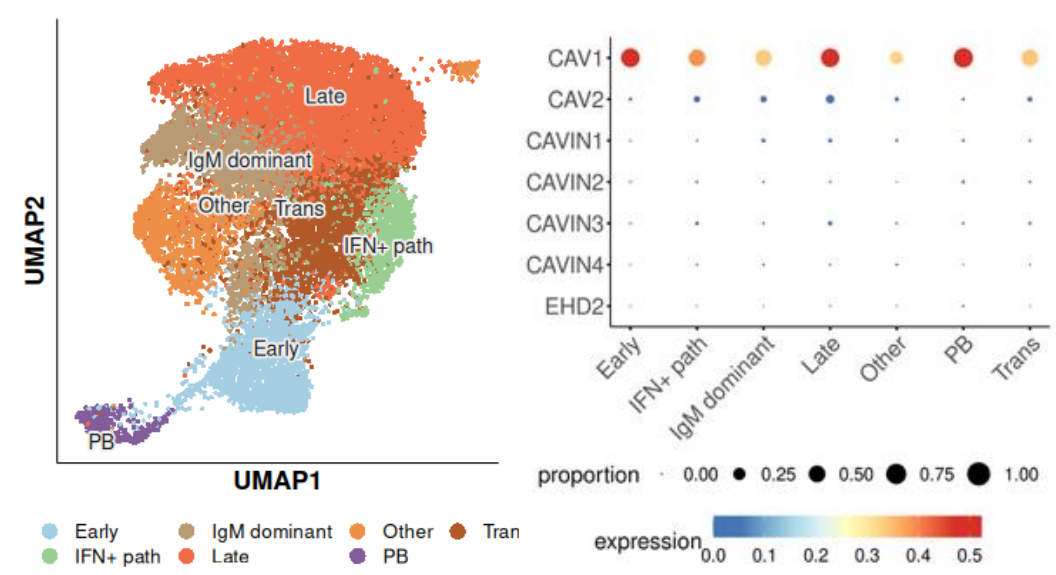

J.

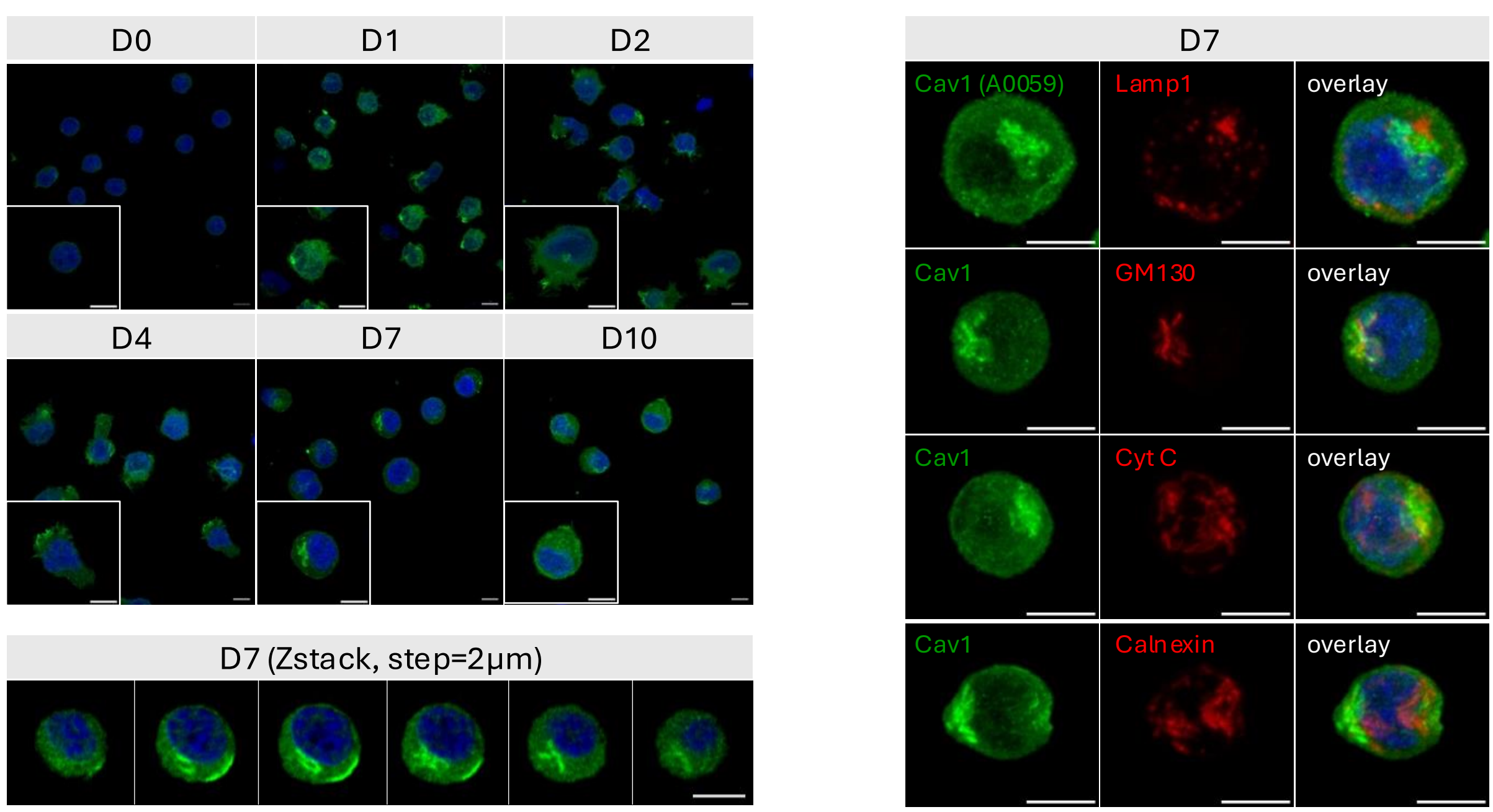

Caveolin1 (A0059) / DAPI

Supplemental Figure 5 (cont'd)

K.

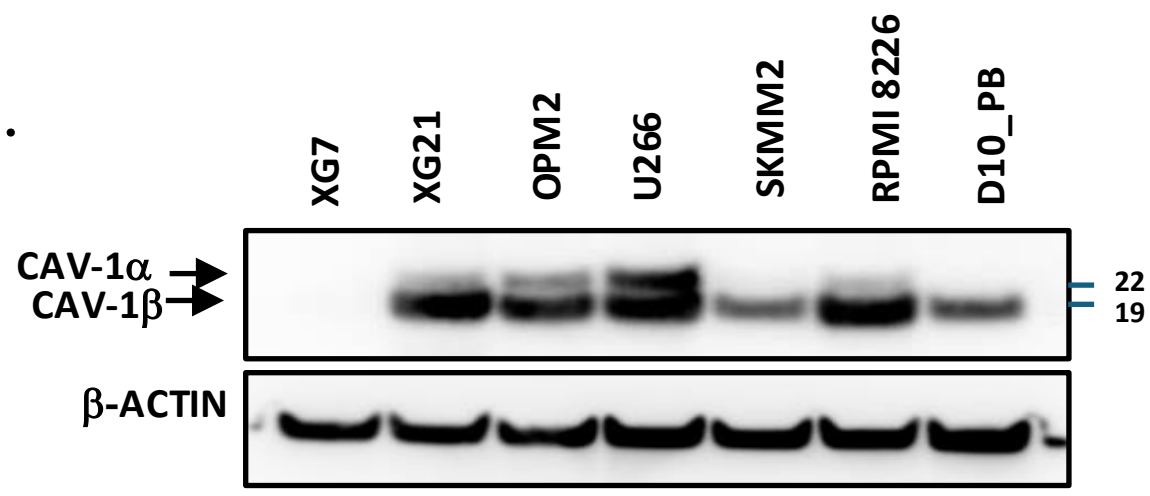

L.

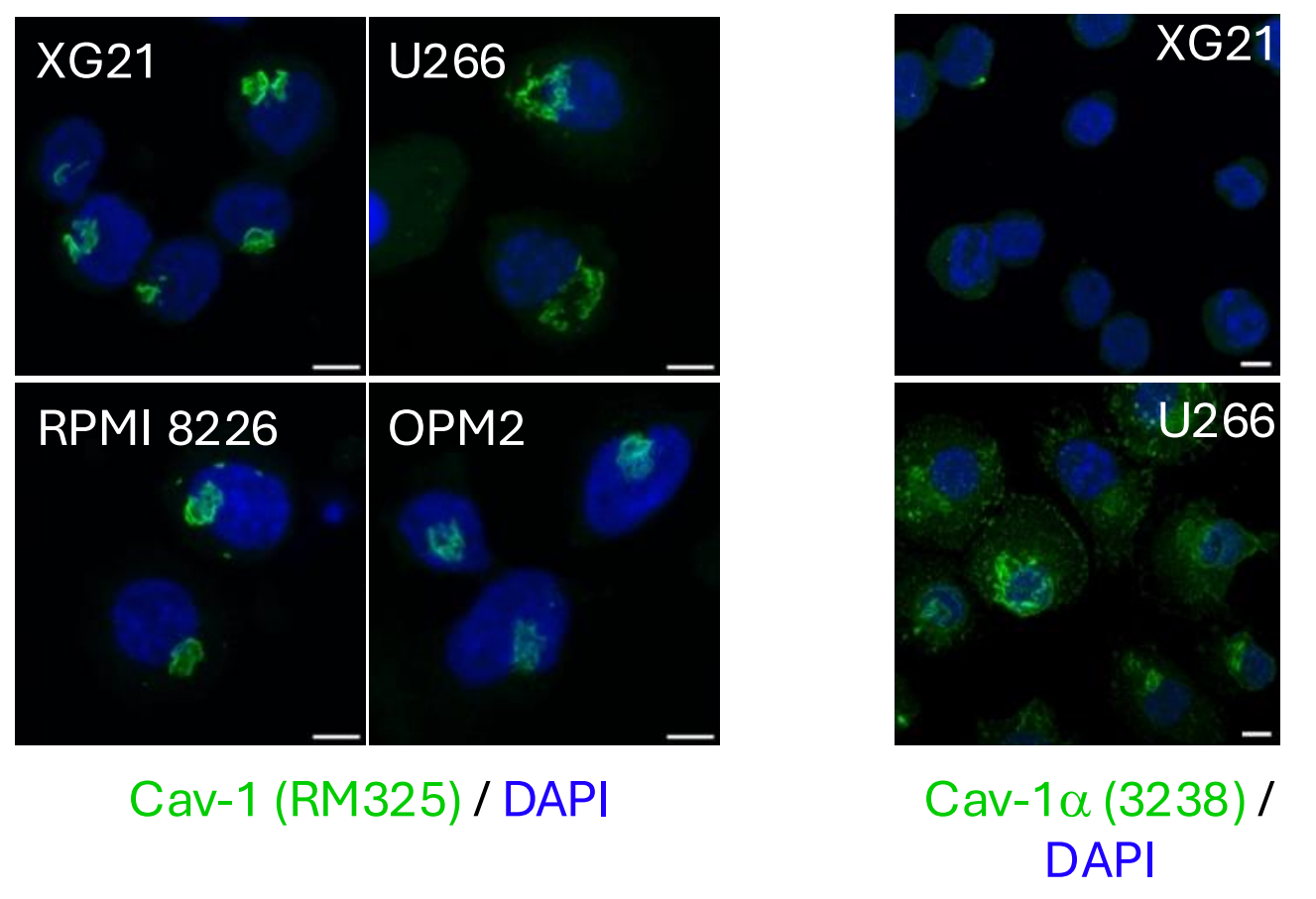

M.

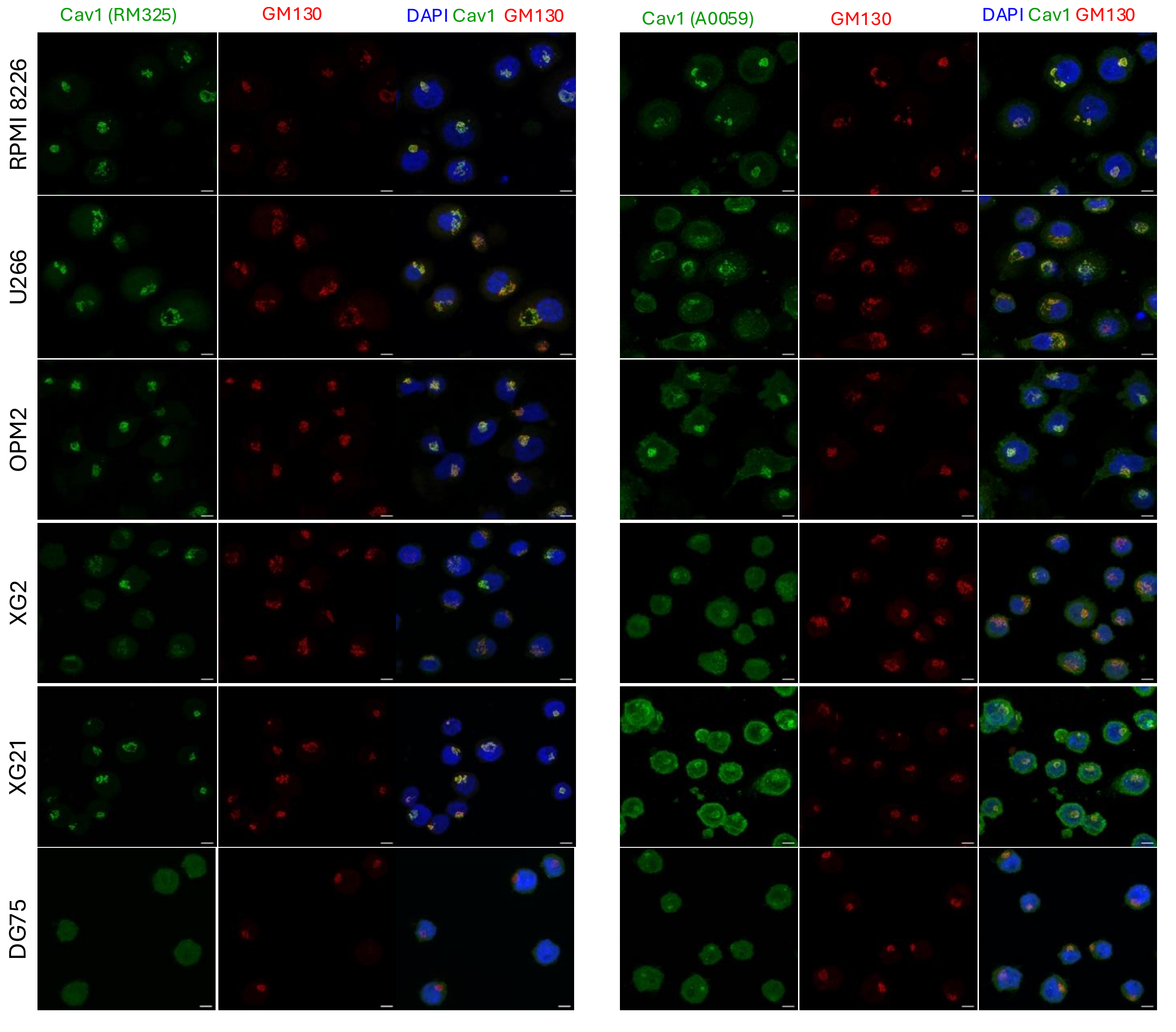

Supplemental Figure 5 (cont'd)

N.

Day1_RACE_sequences (CLUSTAL 0.1.2.4)

TSS Day 1

```
ENST00000614113.5_CAV1      -----------------------------------------------CTTC-------
2_RACE_J1_I15               --ACTCGCTCGGCAGCAGTGGTATCAACGCAGAGTACATGGGGGGACTCTCCGCCAGGC
4_RACE_J1-G15               TTTTTGTTATGGAAGCAGTGGTATCAACGCAGAGTACATGGGGG----------------
8_RACE_J1-E15               --GAGGTCTGGGAAGCAGTGGTATCAACGCAGAGTACATGGGGG----------------

ENST00000614113.5_CAV1      ---------CTCAGTTCCCTTAAAGCACAGCCCAGGG--AAACCTCCTC--ACAGTTT-T
2_RACE_J1_I15               GCCCAGACCGGCAGGTCCCGCA-GGCGGCGCGCGGTGTGTTTGCACTTTCCAAAGTTCTT
4_RACE_J1-G15               ------------------------------------------CACTTTCCAAAGTTCTT
8_RACE_J1-E15               ---CAGACCGGCAGGTCCCGCA-GGCGGCGCGCGGTGTGTTTGCACTTTCCAAAGTTCTT
                                                                        *:* *    *.****   *
```

EXON 1

```
ENST00000614113.5_CAV1      CATCCAGCCACGGGCCAGCATGTCTGGGGGCAAATACGTAGACTCGGAGGGGTGTTCCGA
2_RACE_J1_I15               GAACCATCTCAAGAACTCCT------TCTGCA-TCTTGGCGTCTGGCAGGGGTGTTCCGA
4_RACE_J1-G15               GAACCATCTCAAGAACTCCT------TCTGCA-TCTTGGCGTCTGGCAGGGGTGTTCCGA
8_RACE_J1-E15               GAACCATCTCAAGAACTCCT------TCTGCA-TCTTGGCGTCTGGCAGGGGTGTTCCGA
                             *:*** *  ...*..*: *:         *** :  : *  .*:** * *************

ENST00000614113.5_CAV1      GAGAGGTAGACCTCCCCTCCCCAAACTGCCACCATCACTTCCAACGCCCTCCACGCGCTG
2_RACE_J1_I15               GAGAGGTAGACCTCCCCTCCCCAAACTGCCACCATCACTTCCAACGCCCTCCACGCGCTG
4_RACE_J1-G15               GAGAGGTAGACCTCCCCTCCCCAAACTGCCACCATCACTTCCAACGCCCTCCACGCGCTG
8_RACE_J1-E15               GAGAGGTAGACCTCCCCTCCCCAAACTGCCACCATCACTTCCAACGCCCTCCACGCGCTG
                            ************************************************************

ENST00000614113.5_CAV1      GAGCTCTGCCCGGGTGTGGAAACCTCGTCTTCCAACACGTAGCTGCCCTTCAGCCACCCG
2_RACE_J1_I15               GAGCTCTGCCCGGGTGTGGAAACCTCGTCTTCCAACACGTAGCTGCCCTTCAGCCACCCG
4_RACE_J1-G15               GAGCTCTGCCCGGGTGTGGAAACCTCGTCTTCCAACACGTAGCTGCCCTTCAGCCACCCG
8_RACE_J1-E15               GAGCTCTGCCCGGGTGTGGAAACCTCGTCTTCCAACACGTAGCTGCCCTTCAGCCACCCG
                            ************************************************************

ENST00000614113.5_CAV1      CCCGCAGCCTGGGAGTGCCCTGAGGGTGGGTCGGGGGAGCTGCGCAGGGACATCTCTACA
2_RACE_J1_I15               CCCGCAGCCTGGGAGTGCCCTGAGGGTGGGTCGGGGGAGCTGCGCAGGGACATCTCTACA
4_RACE_J1-G15               CCCGCAGCCTGGGAGTGCCCTGAGGGTGGGTCGGGGGAGCTGCGCAGGGACATCTCTACA
8_RACE_J1-E15               CCCGCAGCCTGGGAGTGCCCTGAGGGTGGGTCGGGGGAGCTGCGCAGGGACATCTCTACA
                            ************************************************************
```

EXON 2

EXON 2_ATG isoform β

```
ENST00000614113.5_CAV1      CCGTTCCCATCCGGGAACAGGGCAACATCTACAAGCCCAACAACAAGGCCATGGCAGACG
2_RACE_J1_I15               CCGTTCCCATCCGGGAACAGGGCAACATCTACAAGCCCAACAACAAGGCCATGGCAGACG
4_RACE_J1-G15               CCGTTCCCATCCGGGAACAGGGCAACATCTACAAGCCCAACAACAAGGCCATGGCAGACG
8_RACE_J1-E15               CCGTTCCCATCCGGGAACAGGGCAACATCTACAAGCCCAACAACAAGGCCATGGCAGACG
                            ************************************************************
```

RACE primer

```
ENST00000614113.5_CAV1      AGCTGAGCGAGAAGCAAGTGTACGACGCGCACA
2_RACE_J1_I15               AGCTGAGCGAGAAGCAAGTGTACGACGCGCACA
4_RACE_J1-G15               AGCTGAGCGAGAAGCAAGTGTACGACGCGCACA
8_RACE_J1-E15               AGCTGAGCGAGAAGCAAGTGTACGACGCGCACA
                            *********************************
```

Day7_RACE_sequences (CLUSTAL 0.1.2.4)

TSS Day 7

```
ENST00000341049.7_CAV1-201      ------------------------------------------------------------
ENST00000405348.6_CAV1-205      ------------------------------------------------------------
7_20_RACE_D7_G15                ----TTACTTCTTTTGGTGGAGGAGTGGTATCAACGCAGAGTACATAGGGGAGGCGCGCC
7_17_RACE_D7_I15                ----TCTTCTCTTTAGTGGAGGAGTGGTATCAACGCAGAGTACATG-GGGAGGCGCGCC
20_RACE_D7_C19                  --TCTCAACCGGTATGGGCAAGCAGTGGTATCAACGCAGAGTACATG--GGAGGCGCGCC

ENST00000341049.7_CAV1-201      ------------------------------------------------------------
ENST00000405348.6_CAV1-205      ------------------------------------------------------------
7_20_RACE_D7_G15                CTGCAGAGTACAGAGGGGTGTGGTGTCCTCTGCGAGATCCTCTTAAAAAGCTGGCTACGC
7_17_RACE_D7_I15                CTGCAGAGTACAGAGGGGTGTGGTGTCCTCTGCGAGATCCTCTTAAAAAGCTGGCTACGC
20_RACE_D7_C19                  CTGCAGAGTACAGAGGGGTGTGGTGTCCTCTGCGAGATCCTCTTAAAAAGCTGGCTACGC

ENST00000341049.7_CAV1-201      ------------------------------------------------------------
ENST00000405348.6_CAV1-205      ------------------------------------------------------------
7_20_RACE_D7_G15                GCAGGCGGTTTCTGTGCACGGAGCCGTAGCTGTCGGAGCGGTTAGTTCGATTTCGAGCTC
7_17_RACE_D7_I15                GCAGGCGGTTTCTGTGCACGGAGCCGTAGCTGTCGGAGCGGTTAGTTCGATTTCGAGCTC
20_RACE_D7_C19                  GCAGGCGGTTTCTGTGCACGGAGCCGTAGCTGTCGGAGCGGTTAGTTCGATTTCGAGCTC

ENST00000341049.7_CAV1-201      ------------------------------------------------------------
ENST00000405348.6_CAV1-205      ------------------------------------------------------------
7_20_RACE_D7_G15                GAGGTTTCCCCCGCCGCCAGGCTGACTTCTCATCGCTTGTTTTTCTTTTTGCATTTTTCC
7_17_RACE_D7_I15                GAGGTTTCCCCCGCCGCCAGGCTGACTTCTCATCGCTTGTTTTTCTTTTTGCATTTTTCC
20_RACE_D7_C19                  GAGGTTTCCCCCGCCGCCAGGCTGACTTCTCATCGCTTGTTTTTCTTTTTGCATTTTTCC
```

EXON 1_ATG isoform α

```
ENST00000341049.7_CAV1-201      --------------------------ATGTCTGGGGGCAAATACGTAGACTCGGAGGGAC
ENST00000405348.6_CAV1-205      ------------------------------------------------------------
7_20_RACE_D7_G15                TCCCACCGCCGTTGCCGCCCTCCCCGTCCTGGCCGTCCGCCCTCCGCCCTCTGCAGGGAC
7_17_RACE_D7_I15                TCCCACCGCCGTTGCCGCCCTCCCCGTCCTGGCCGTCCGCCCTCCGCCCTCTGCAGGGAC
20_RACE_D7_C19                  TCCCACCGCCGTTGCCGCCCTCCCCGTCCTGGCCGTCCGCCCTCCGCCCTCTGCAGGGAC
```

EXON 2_ATG isoform β

```
ENST00000341049.7_CAV1-201      ATCTCTACACCGTTCCCATCCGGGAACAGGGCAACATCTACAAGCCCAACAACAAGGCCA
ENST00000405348.6_CAV1-205      -----------------------------------------------------------A
7_20_RACE_D7_G15                ATCTCTACACCGTTCCCATCCGGGAACAGGGCAACATCTACAAGCCCAA-----------
7_17_RACE_D7_I15                ATCTCTACACCGTTCCCATCCGGGAACAGGGCAACATCTACAAGCCCAACAACAAGGCCA
20_RACE_D7_C19                  ATCTCTACACCGTTCCCATCCGGGAACAGGGCAACATCTACAAGCCCAACAACAAGGCCA
```

RACE primer

```
ENST00000341049.7_CAV1-201      TGGCAGACGAGCTGAGCGAGAAGCAAGTGTACGACGCGCACA
ENST00000405348.6_CAV1-205      TGGCAGACGAGCTGAGCGAGAAGCAAGTGTACGACGCGCACA
7_20_RACE_D7_G15                ------------------------------------------
7_17_RACE_D7_I15                TGGCAGACGAGCTGAGCGAGAAGCAAGTGTACGACGCGCACA
20_RACE_D7_C19                  TGGCAGACGAGCTGAGCGAGAAGCAAGTGTACGACGCGCACA
```

Supplemental Figure 5 (cont'd)

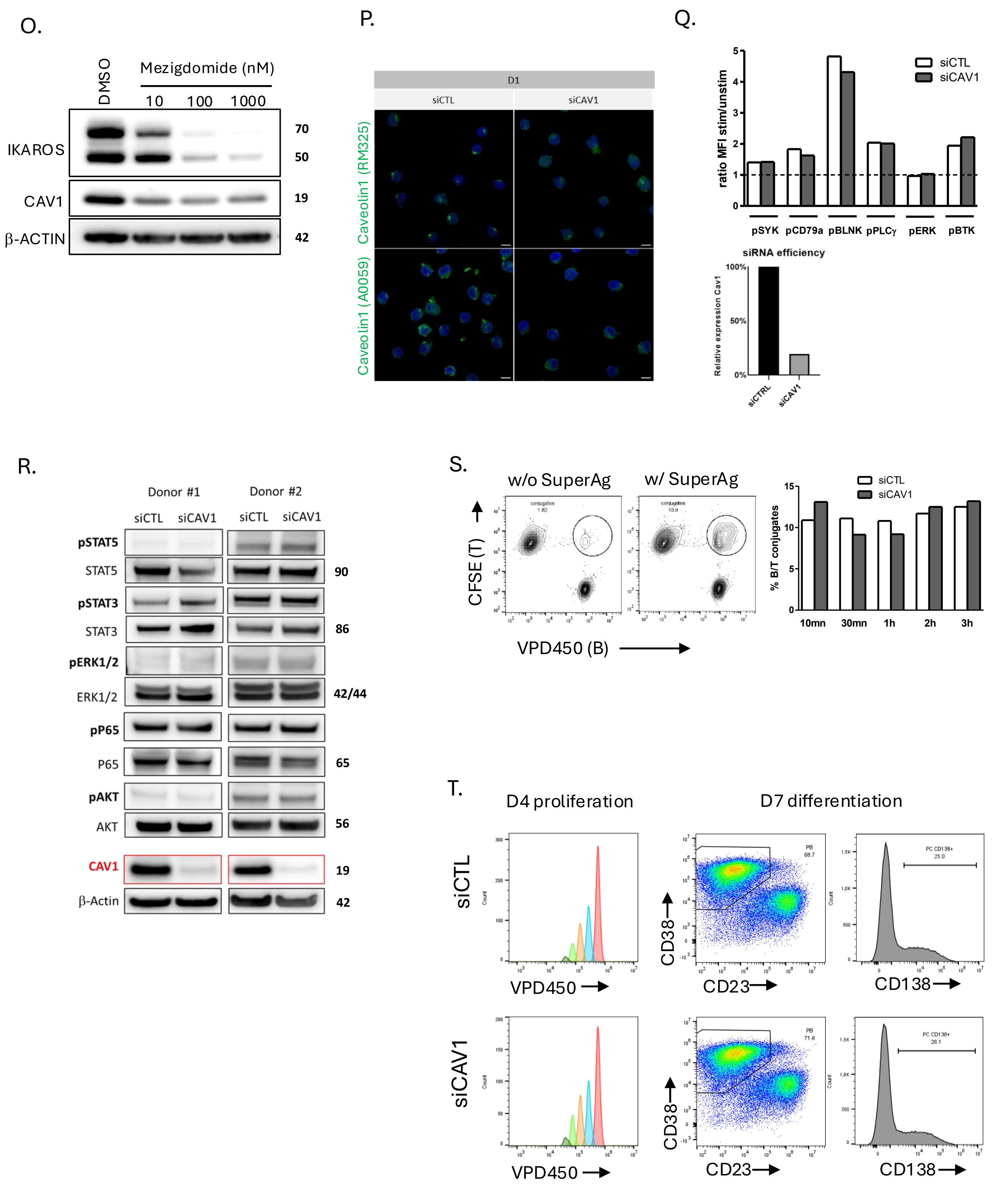

a–c) Upper panels: UMAP plots of published single-cell transcriptomic datasets: (a) human total adipose tissue cells (Hildreth et al., 2021; DOI: 10.1038/s41590-021-00922-4), (b) tonsillar B cells (King et al., 2021; DOI: 10.1126/sciimmunol.abe6291), and (c) bone marrow antibody-secreting cells (Duan et al., 2023; DOI: 10.1016/j.celrep.2023.112682). Clusters are colored by original annotations. Lower panels: Projection of *CAV1* expression onto UMAP; red intensity indicates higher log-normalized expression.

d) UMAP plot of murine B cells 7 days post-immunization (Duan et al., 2021, doi:10.1016/j.immuni.2021.08.028) with indicated gene expression.

e) *CAV1* expression levels across B cell subsets measured by bulk RNA-seq. Bars represent mean value.

f) Expression of *CAV1*α–specific transcripts in in vivo B cell subsets. Bars represent mean value.

g) UMAP plot of CAV1 (green) and PRDM1 (red) in the full B cell differentiation (upper) and activated B cells (lower) datasets. Table shows % cells expressing CAV1, PRDM1, both, or neither in non-plasma cell clusters.

h) UMAP plots of *CAV1*, *CAVIN1* and *EHD2* expression during B cell differentiation.

i) UMAP plot presenting clusters of bone marrow antibody-secreting cells and bubble plot showing *CAV1-2*, *CAVIN1-3* and *EHD2* expression in each cluster.

j) Immunofluorescence and z-stack analysis of Cav-1 (green, A0059 antibody) localization at indicated time points (left) and co-staining with organelle markers (red): LAMP1 (lysosomes), GM130 (Golgi), CytC (mitochondria), and Calnexin (ER) in day 7 plasmablasts. Nuclei counterstained with DAPI. Scale bars = 5μm.

k) Western blot detection of Cav-1 isoforms in multiple myeloma (MM) cell lines compared with in vitro–generated plasmablasts (day 10).

l) Immunofluorescence detection of total Cav-1 (green, RM325 antibody, left panel) and Cav-1α (green, 3238 antibody, right panel) in MM cell lines. Scale bars = 5μm.

m) Co-staining of Cav-1 (green, RM325 or A0059 antibodies) and the Golgi marker GM130 (red) in multiple myeloma cell lines. Nuclei counterstained with DAPI. Scale bars = 5μm.

n) Sequences of *CAV1* transcripts at day 1 and day 7 identified by RACE-PCR; primer positions in blue aligned to sequences published in Ensembl.

o) Western blot detection of Ikaros and Cav-1 in stimulated B cells treated 16h with different doses of Mezigdomide (nM) or DMSO.

p) Immunofluorescence detection of Cav-1 (green, RM325 or A0059) in day 1 activated B cells electroporated with siCAV1 or siCTL; nuclei stained with DAPI (blue). Scale bars = 5μm.

q) Left: flow-cytometric analysis of Syk, CD79a, BLNK, PLCγ, ERK1/2, and BTK phosphorylation after BCR stimulation with $F(ab')_2$ anti-IgA/IgG/IgM in CD40L-activated B cells electroporated with siCAV1 or siCTL. Results are expressed as the ratio of mean fluorescence intensity (MFI) between stimulated and unstimulated conditions. Right: siRNA efficiency assessed by CAV1 mRNA down-regulation.

r) Western blot analysis of STAT5, STAT3, ERK1/2, p65 (RelA), and AKT phosphorylation in day-1 activated B cells ± siCAV1.

s) Left: flow-cytometric dot plots showing B/T-cell conjugates stained (VPD450 B cells, CFSE T cells) ± superantigens (TSST/SEA/SEB). Right: % conjugates at indicated times, ± siCAV1. Representative of two independent experiments.

t) Flow-cytometric histograms and dot plots of B cell proliferation at day 4 (VPD450 dilution) and differentiation at day 7 (PB $CD23^{-}CD38hi$; PC $CD23^{-}CD38hiCD138^{+}$) ± siCAV1. Representative of two independent experiments.

Figure S6. Validation of Cav-1 silencing and functional analyses, related to figure 6.

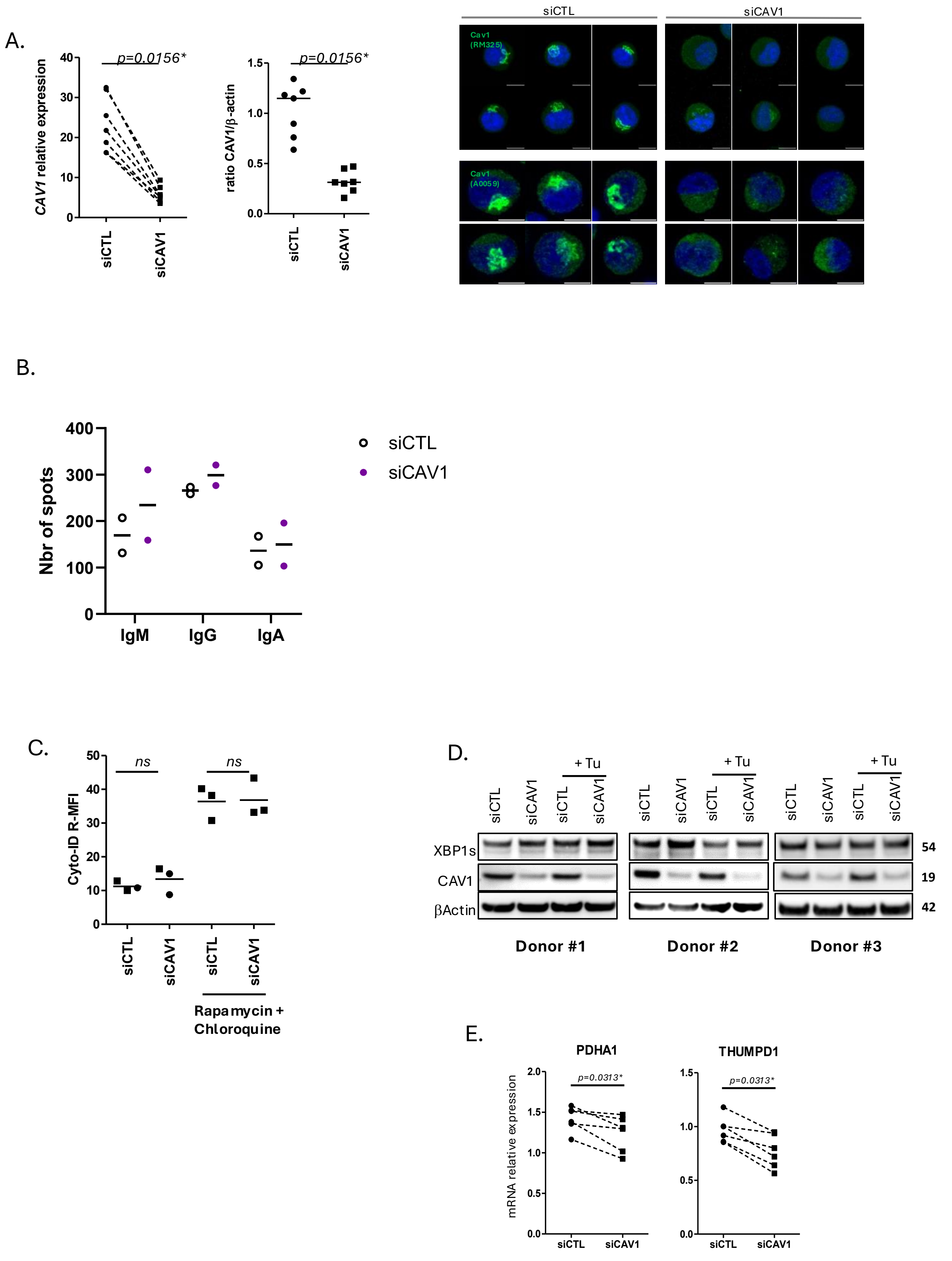

a) Validation of CAV1 silencing at the mRNA and protein levels. Left, RT-qPCR analysis of CAV1 mRNA expression. Middle, quantification of Cav-1/β-actin protein ratios from Western blots using ImageJ. Right, representative immunofluorescence staining confirming reduced Cav-1 expression following siCAV1 treatment.

b) Frequency of antibody-secreting cells following CAV1 silencing. IgM-, IgG-, and IgA-secreting cells were quantified by ELISPOT on day 7 after siCTL (squares) or siCAV1 (circles) electroporation (n=2 independent experiments). Each symbol represents one donor. A trend toward increased numbers of immunoglobulin-secreting cells was observed following CAV1 silencing. Bars represent mean value.

c) Autophagic flux assessed by Cyto-ID staining. Cells electroporated with siCTL or siCAV1 on day 5 were analyzed on day 8. Rapamycin (500 nM) plus chloroquine (10 μM) served as positive controls. Results are expressed as the ratio of mean fluorescence intensity (MFI) between stained and unstained cells. Bars represent mean value.

d) Western blot analysis of XBP1s and Cav-1 expression in day 7 B cells from three independent donors following siCAV1 or siCTL electroporation on day 5. When indicated, cells were treated with tunicamycin (4 μg/mL) for 4 h before protein extraction.

e) RT-qPCR analysis of PDHA1 and THUMPD1 mRNA expression in day 7 plasmablasts following siCAV1 or siCTL electroporation on day 5.

For panels a, b, and d, P values were determined using the paired Wilcoxon test (ns, not significant).

Figure S7. Cav-1β interacts with lysosomal and mTORC1-associated protein complexes, related to Figure 7.

A.

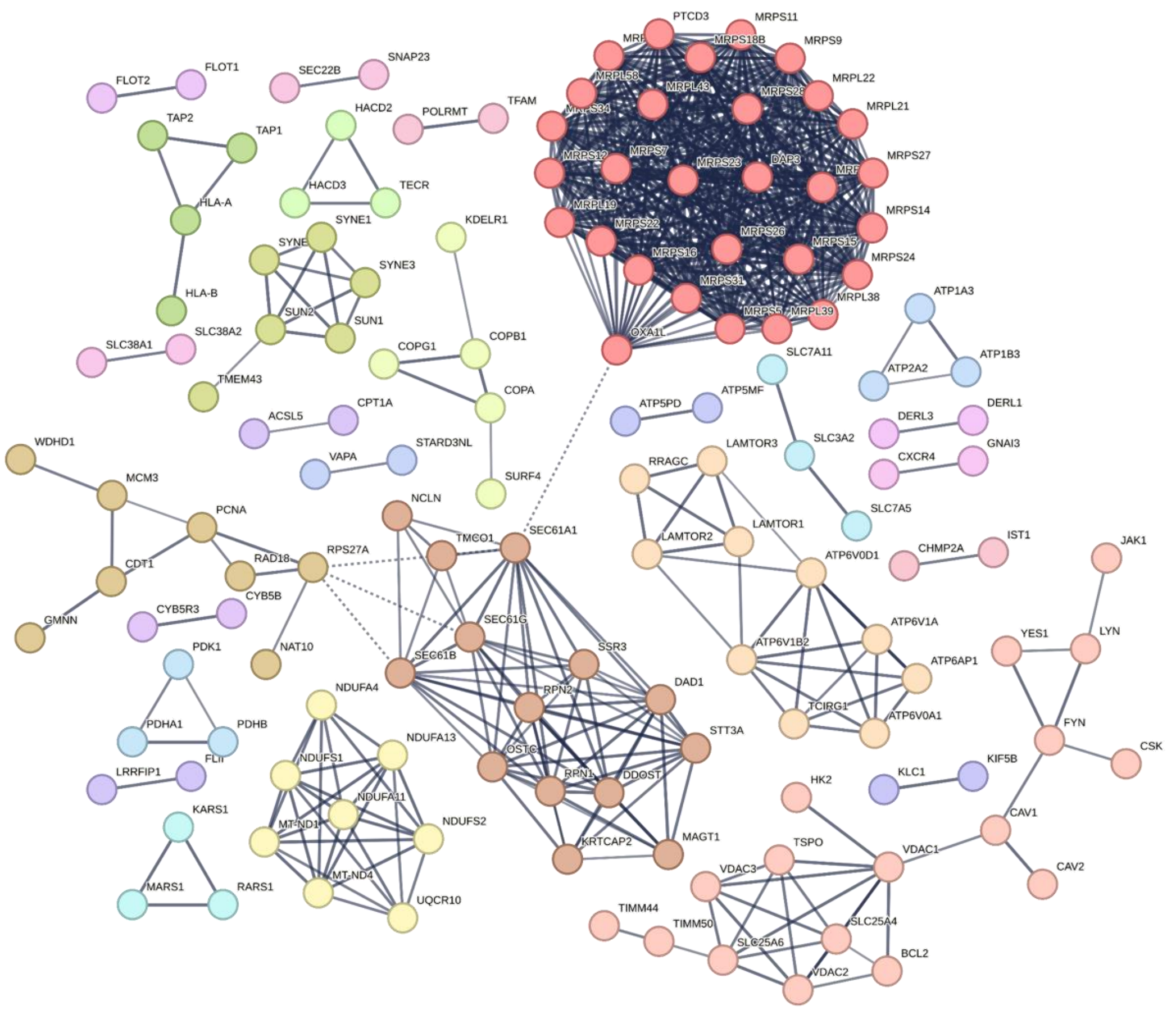

color	cluster Id	gene count	description
	Cluster 1	29	Mitochondrial translation
	Cluster 2	17	+ Mitochondrial transport
	Cluster 3	14	Oligosaccharyltransferase complex
	Cluster 4	10	+ Amino acids regulate mTORC1
	Cluster 5	8	Nuclear DNA replication
	Cluster 6	8	+ Oxidative phosphorylation
	Cluster 7	6	+ Cytoskeleton-nuclear membrane anchor activity
	Cluster 8	5	+ COPI-dependent Golgi-to-ER retrograde traffic
	Cluster 9	4	+ Antigen processing and presentation of endogenous peptide antigen via MHC cl...
	Cluster 10	3	+ Fatty acid elongation
	Cluster 11	3	+ Aminoacyl-tRNA biosynthesis
	Cluster 12	3	+ Basigin interactions
	Cluster 13	3	+ Mitochondrial pyruvate dehydrogenase complex
	Cluster 14	3	+ Cation-transporting ATPase complex
	Cluster 15	2	Endoplasmic reticulum-endosome membrane contact site, and C syndrome
	Cluster 16	2	+ CF(0)
	Cluster 17	2	+ Stress granule disassembly
	Cluster 18	2	LRRFIP family, and EF-hand domain-containing protein D1/2
	Cluster 19	2	ACSL5, CPT1A
	Cluster 20	2	Nitric-oxide synthase complex
	Cluster 21	2	+ Flotillin complex
	Cluster 22	2	+ Hrd1p ubiquitin ligase ERAD-L complex
	Cluster 23	2	CXCR4, GNAI3
	Cluster 24	2	+ Neutral amino acid:sodium symporter activity
	Cluster 25	2	+ SNARE interactions in vesicular transport
	Cluster 26	2	+ Mitochondrial transcription initiation
	Cluster 27	2	+ ESCRT III complex disassembly

B.

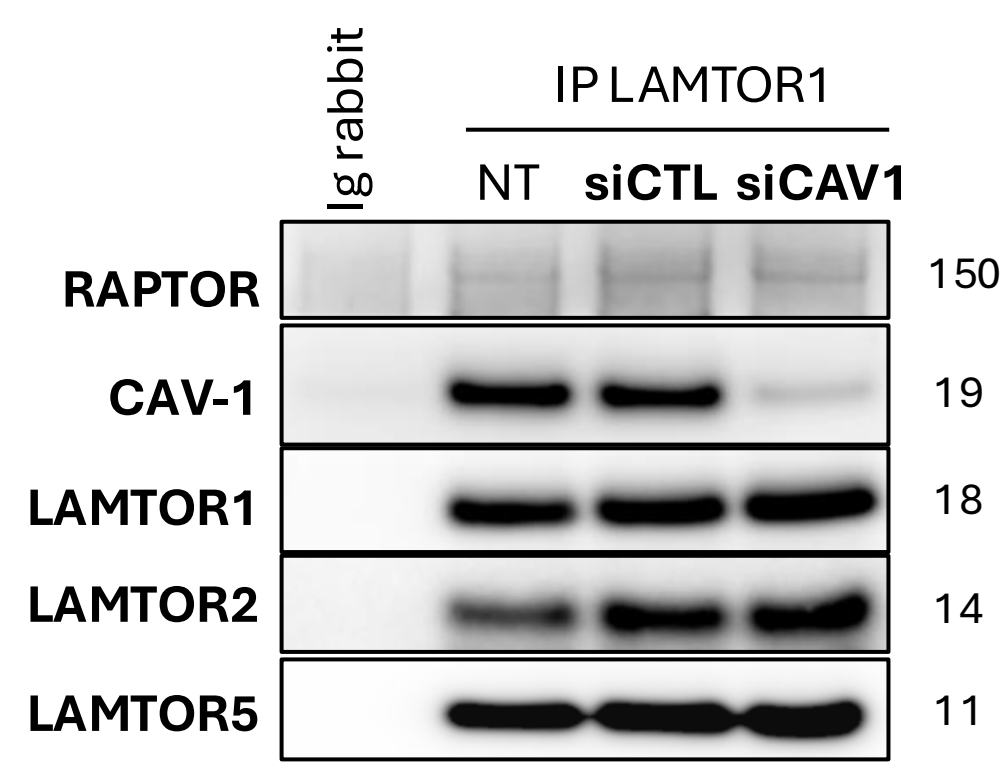

C.

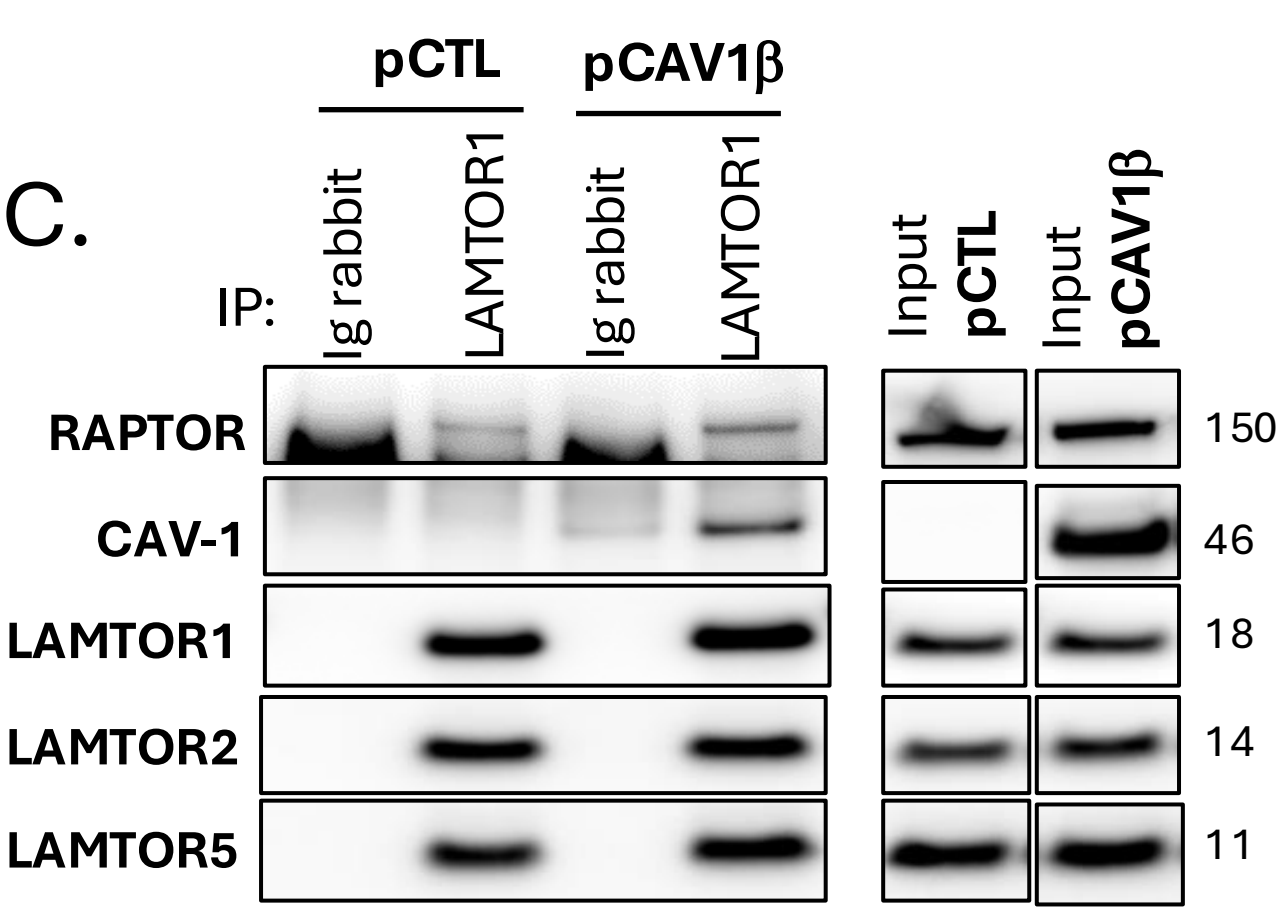

a) STRING interaction network of Cav-1 partners (score ≥0.7). Edges indicate membership in the same complex; line thickness reflects confidence. Colors indicate clusters (k-means); dotted lines represent interactions between clusters. Disconnected nodes are hidden for clarity.

b) XG21 cells transfected with siCTL or siCAV1 for 48 h, followed by detection of RAPTOR, Cav-1, LAMTOR1, LAMTOR 2, and LAMTOR5 by western blot after LAMTOR1 immunoprecipitation (representative of one experiment).

c) DG-75 Burkitt's lymphoma cells transfected with a plasmid expressing Cav-1β (pCAV1β) or control plasmid (pCTL) for 48 h, followed by detection of RAPTOR, Cav-1, LAMTOR1, LAMTOR2, and LAMTOR5 by western blot after LAMTOR1 immunoprecipitation (representative of one experiment).

Data S1. Western blots raw data for figures and supplemental figures

Fig 5B

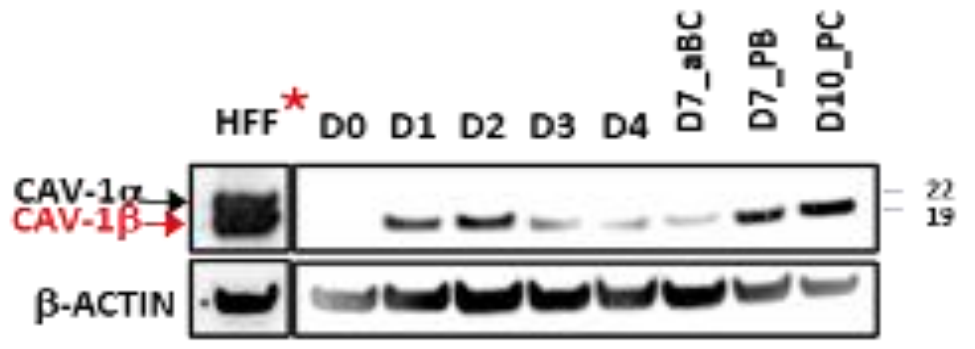

1 D0
2 D1
3 D2
4 D3
5 D4
6 D7_aBC
7 D7_PB
8 D10_PB

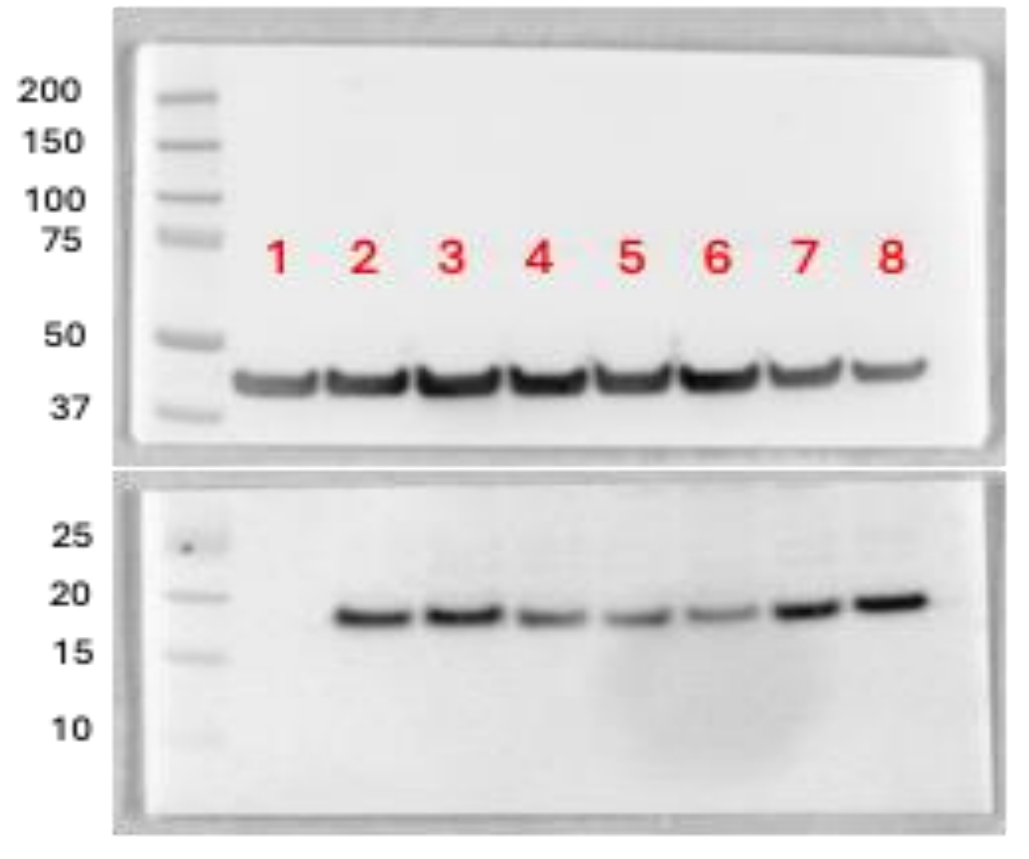

WB βActin

WB CAV1

*HFF -> see raw WB Fig S5K

Fig 5F

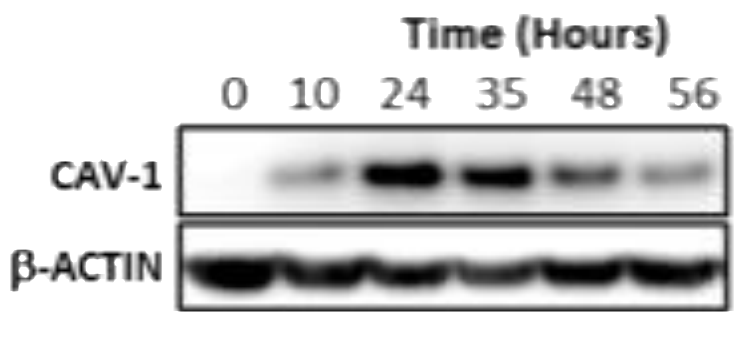

1 0h
2 10h
3 24h
4 35h
5 48h
6 56h

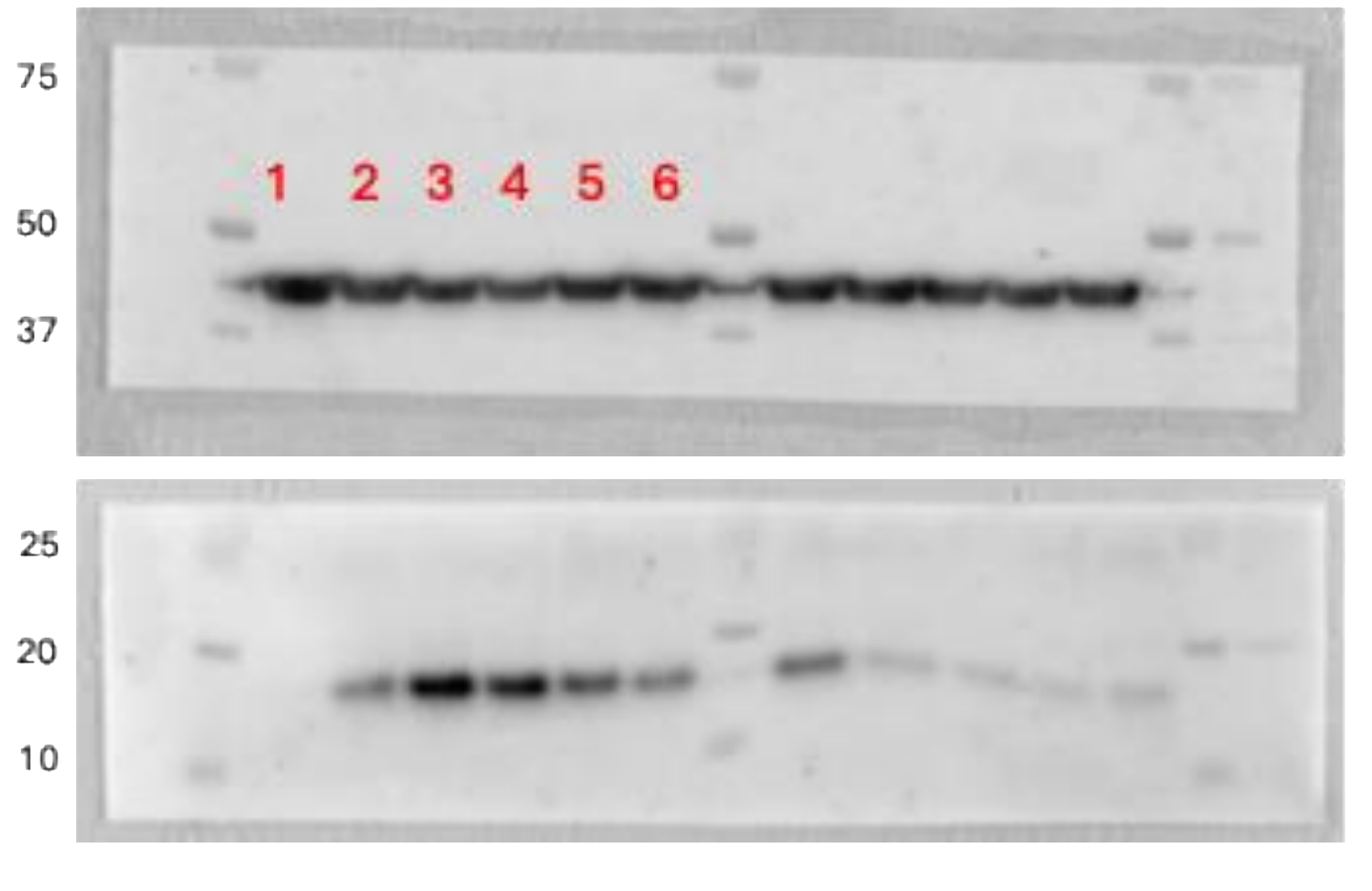

WB βActin

WB CAV1

Fig S5K

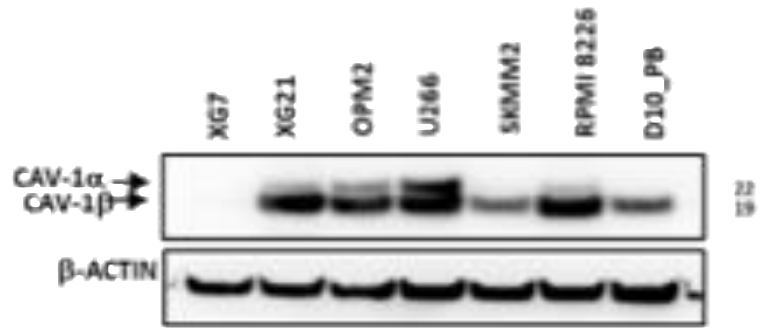

1 XG7
2 XG21
3 OPM2
4 U266
5 SKMM2
6 RPMI8226
7 D10-PB
8 HFF -> see Fig 5B

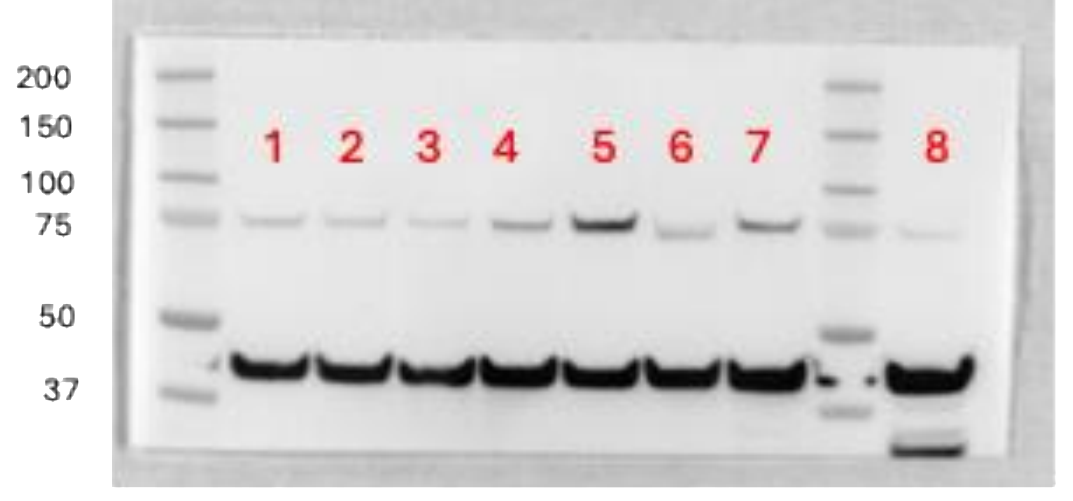

WB βActin

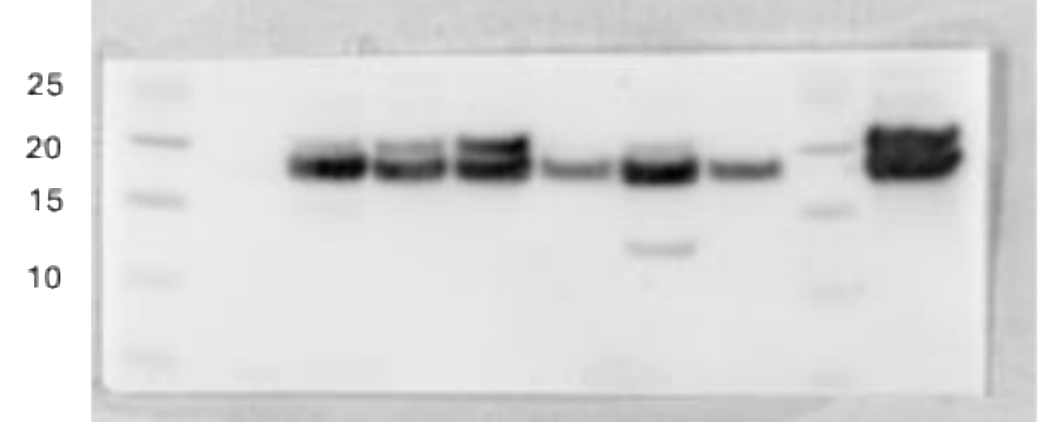

WB CAV1

Fig S5O

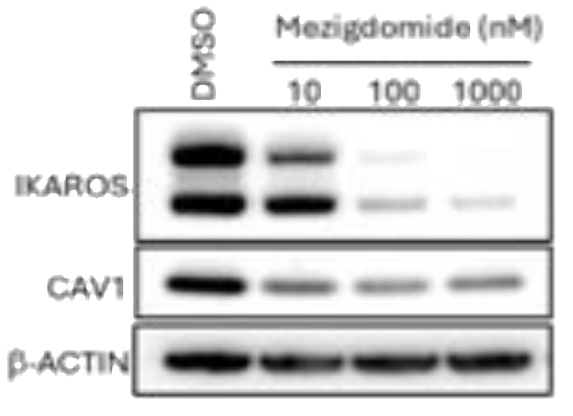

1 DMSO
2 Mez 10 nM
3 Mez 100 nM
4 Mez 1000 nM

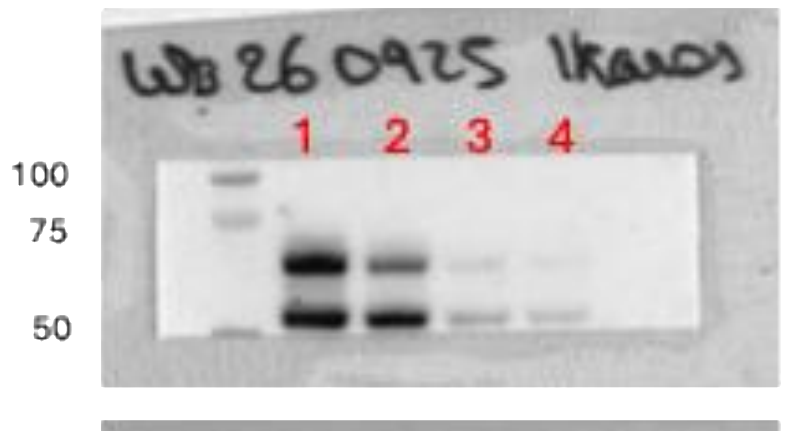

WB IKAROS

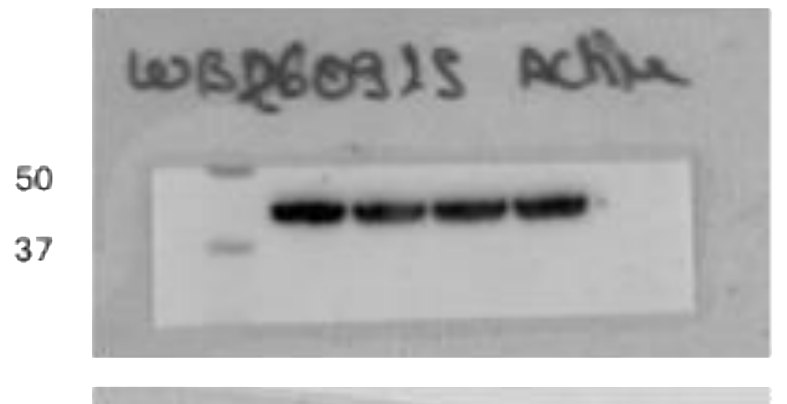

WB βActin

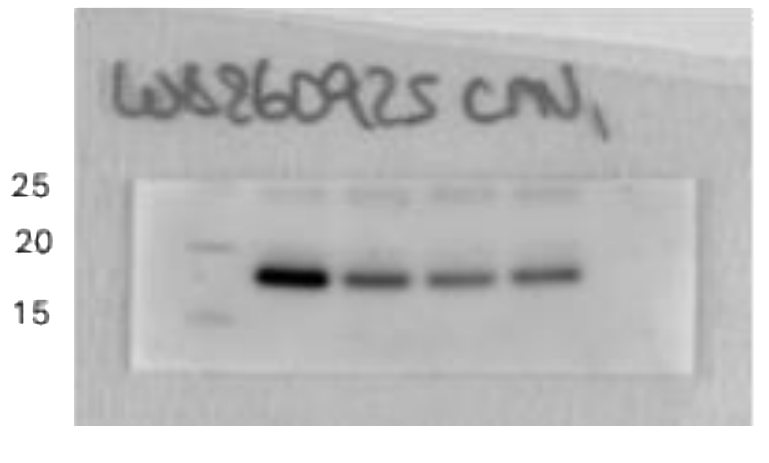

WB CAV1

Fig S5R

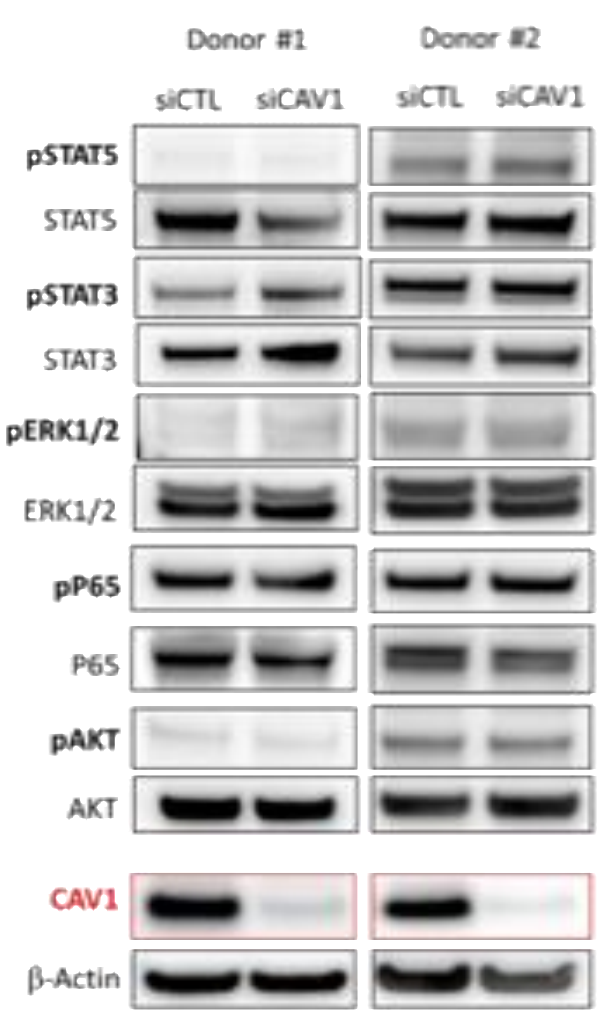
Donor #1
Donor #2
siCTL
siCAV1
siCTL
siCAV1
pSTAT5
STAT5
pSTAT3
STAT3
pERK1/2
ERK1/2
pP65
P65
pAKT
AKT
CAV1
β-Actin

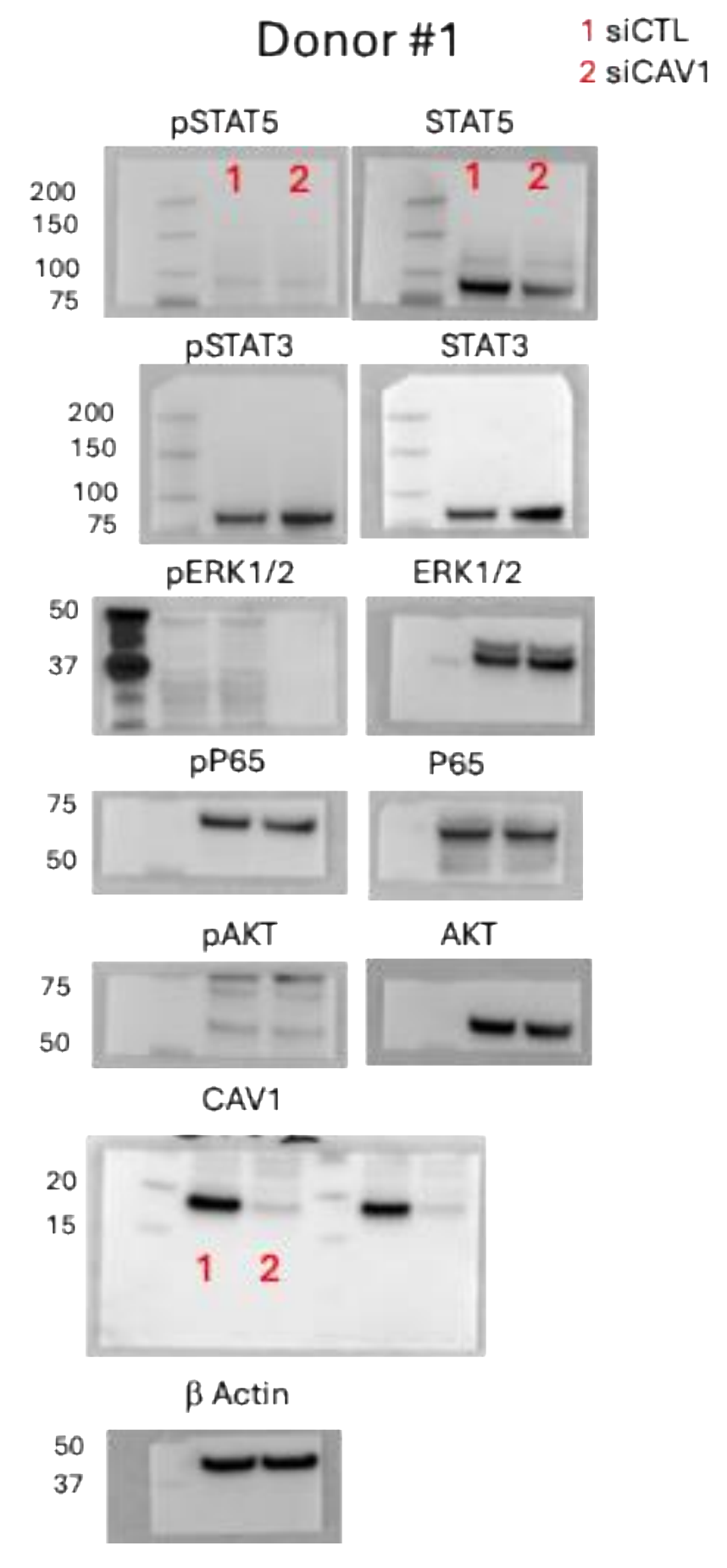
Donor #1
1 siCTL
2 siCAV1
pSTAT5
STAT5
1 2
1 2
200
150
100
75
pSTAT3
STAT3
200
150
100
75
pERK1/2
ERK1/2
50
37
pP65
P65
75
50
pAKT
AKT
75
50
CAV1
20
15
1 2
β Actin
50
37

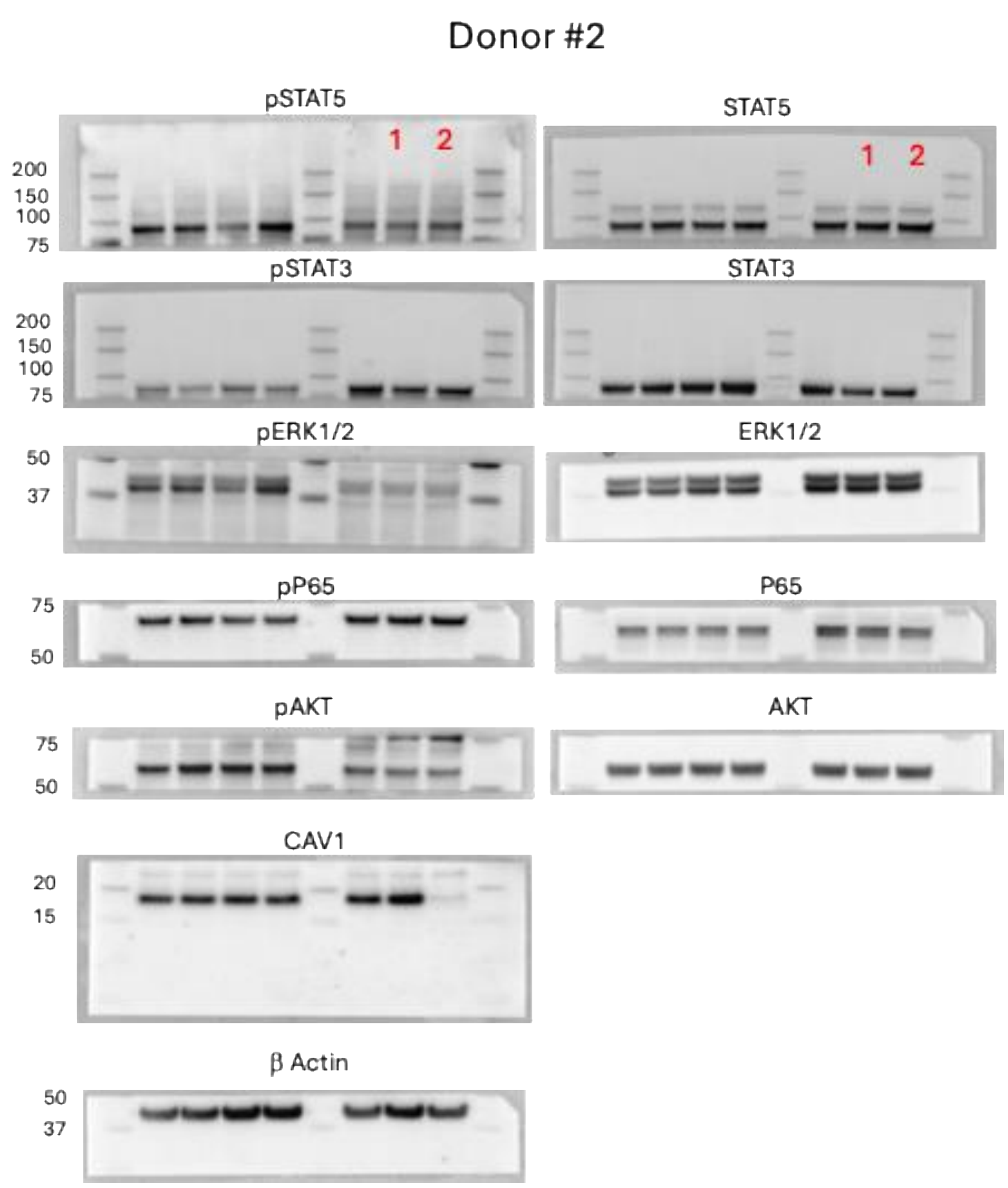
Donor #2
pSTAT5
STAT5
1 2
1 2
200
150
100
75
pSTAT3
STAT3
200
150
100
75
pERK1/2
ERK1/2
50
37
pP65
P65
75
50
pAKT
AKT
75
50
CAV1
20
15
β Actin
50
37

Fig 6C

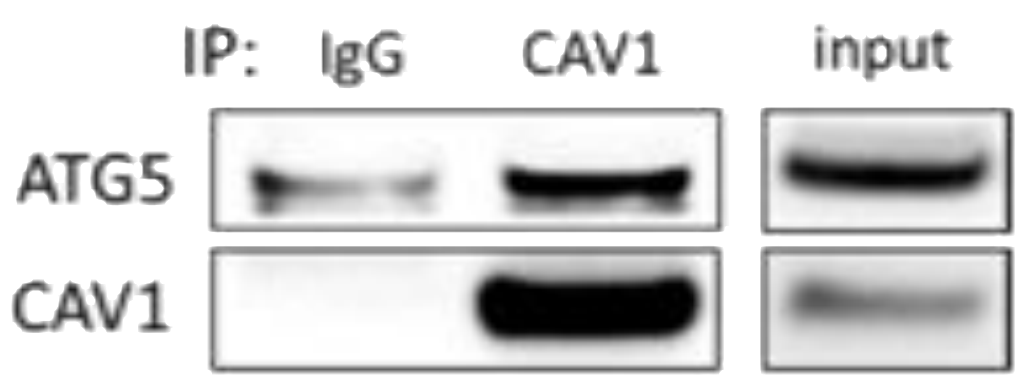

1 IP Ig Goat / D7 plasmablasts
2 IP CAV1 / D7 plasmablasts
3 input D7 plasmablasts

WB ATG5

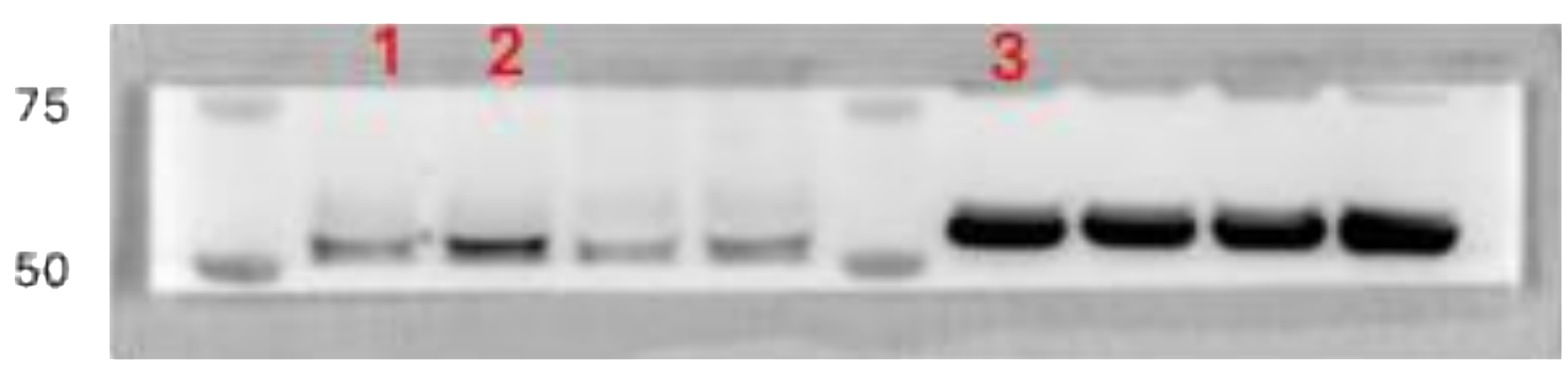

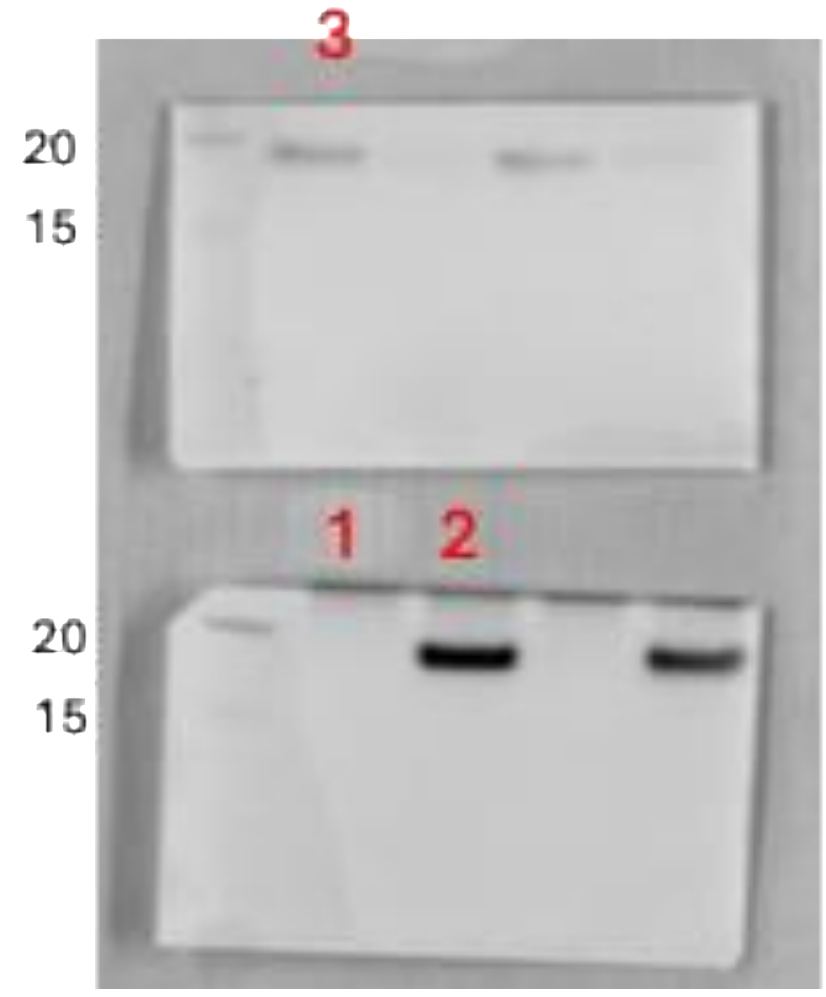

WB Cav-1

Fig S6D

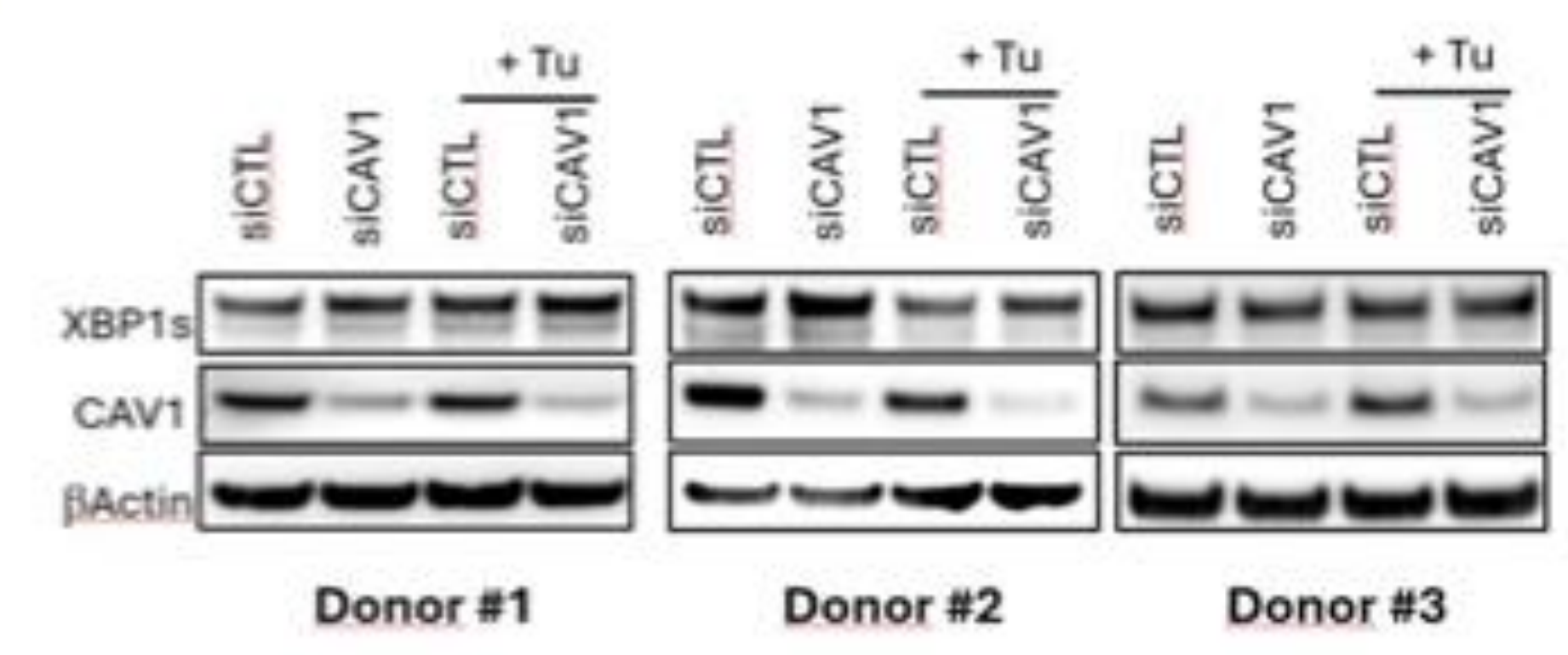

1 siCTL
2 siCAV1
3 siCTL+Tu
4 siCAV1+Tu

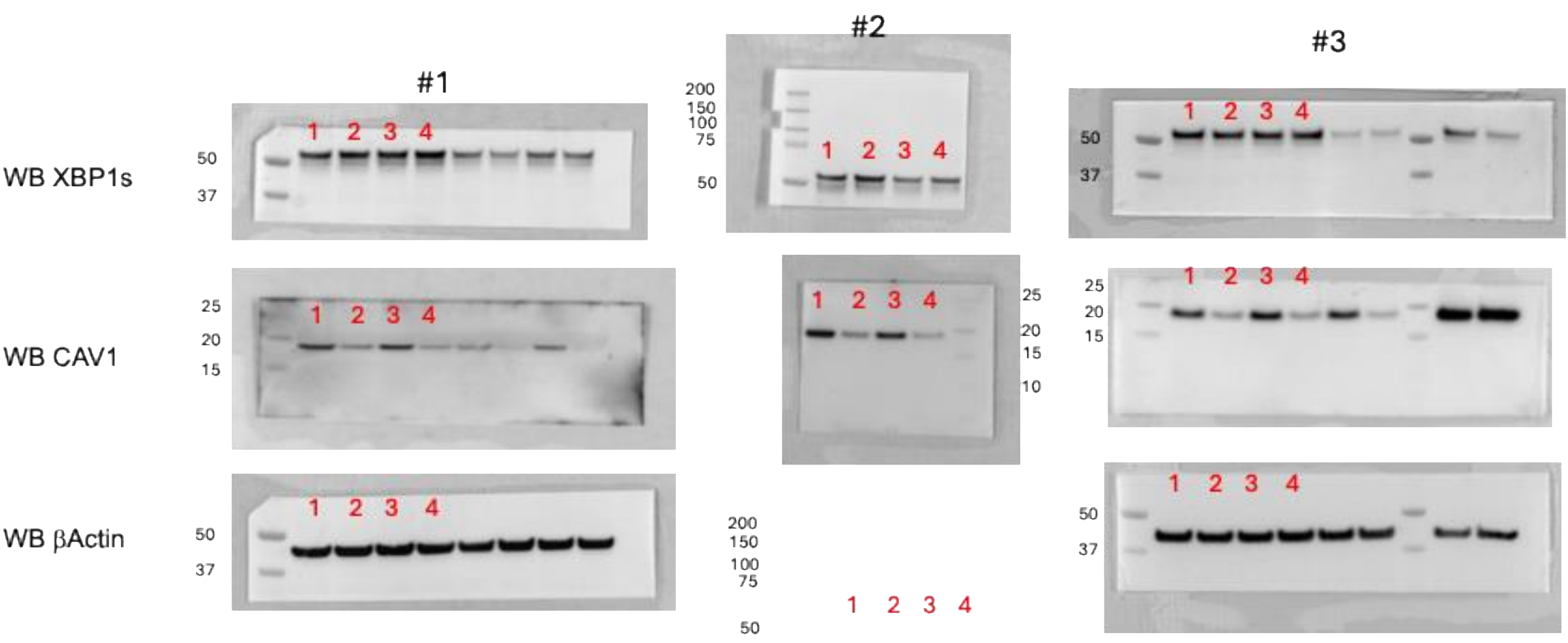

Fig 7D

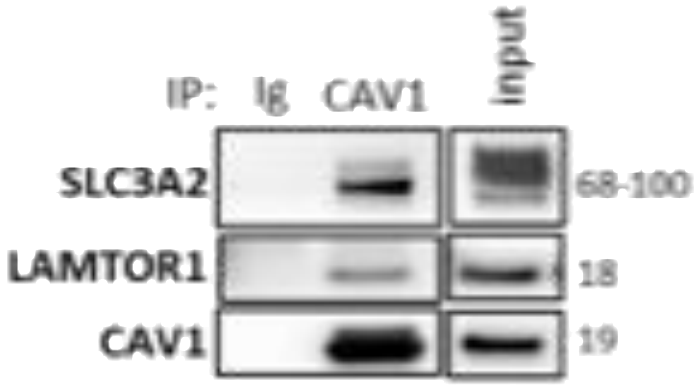

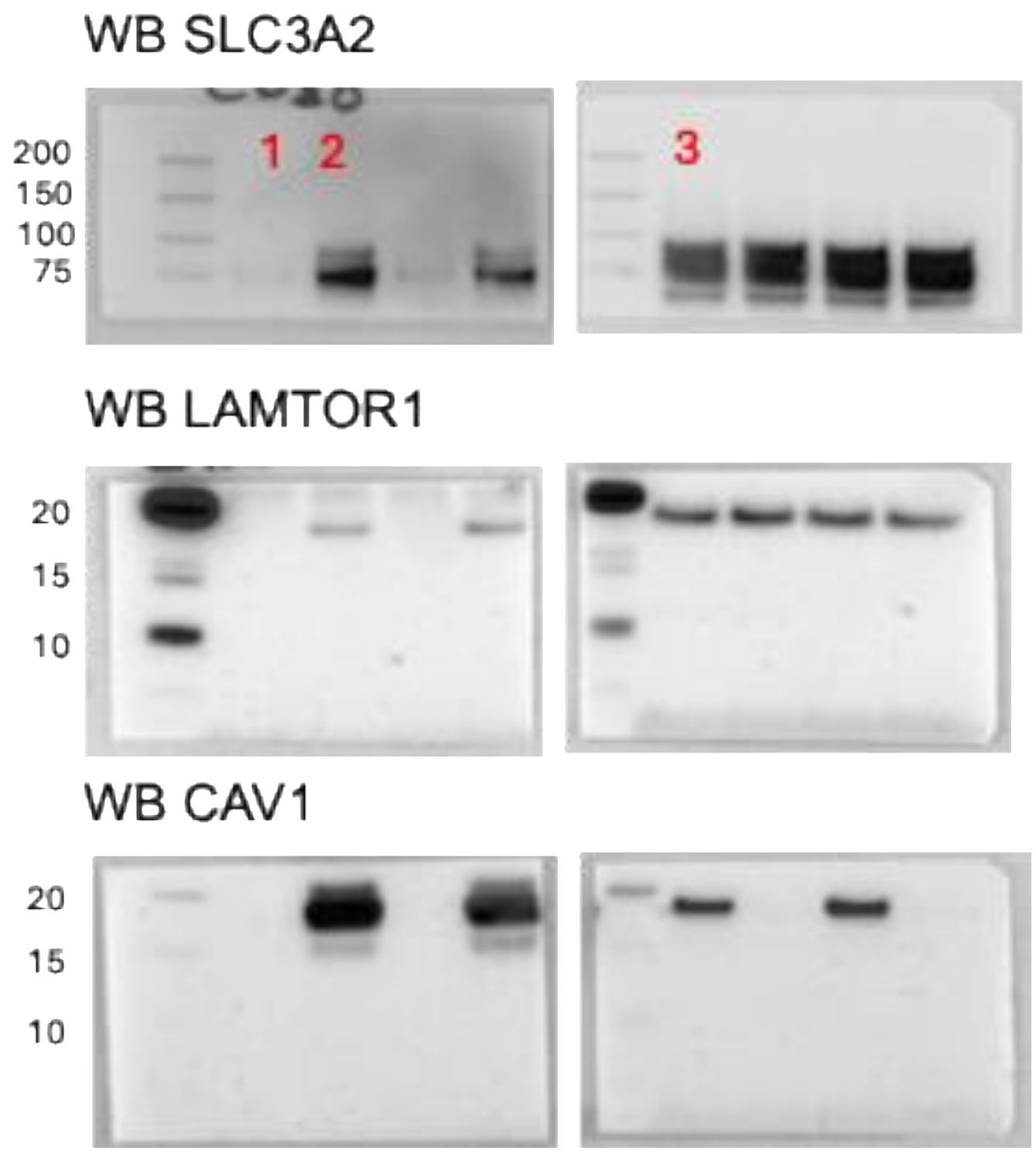

1 IP Ig Goat / XG21
2 IP CAV1 / XG21
3 input XG21

Fig 7E

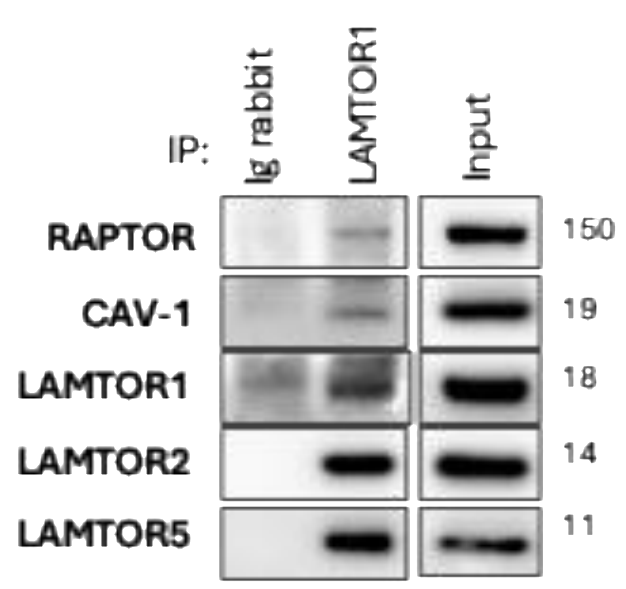

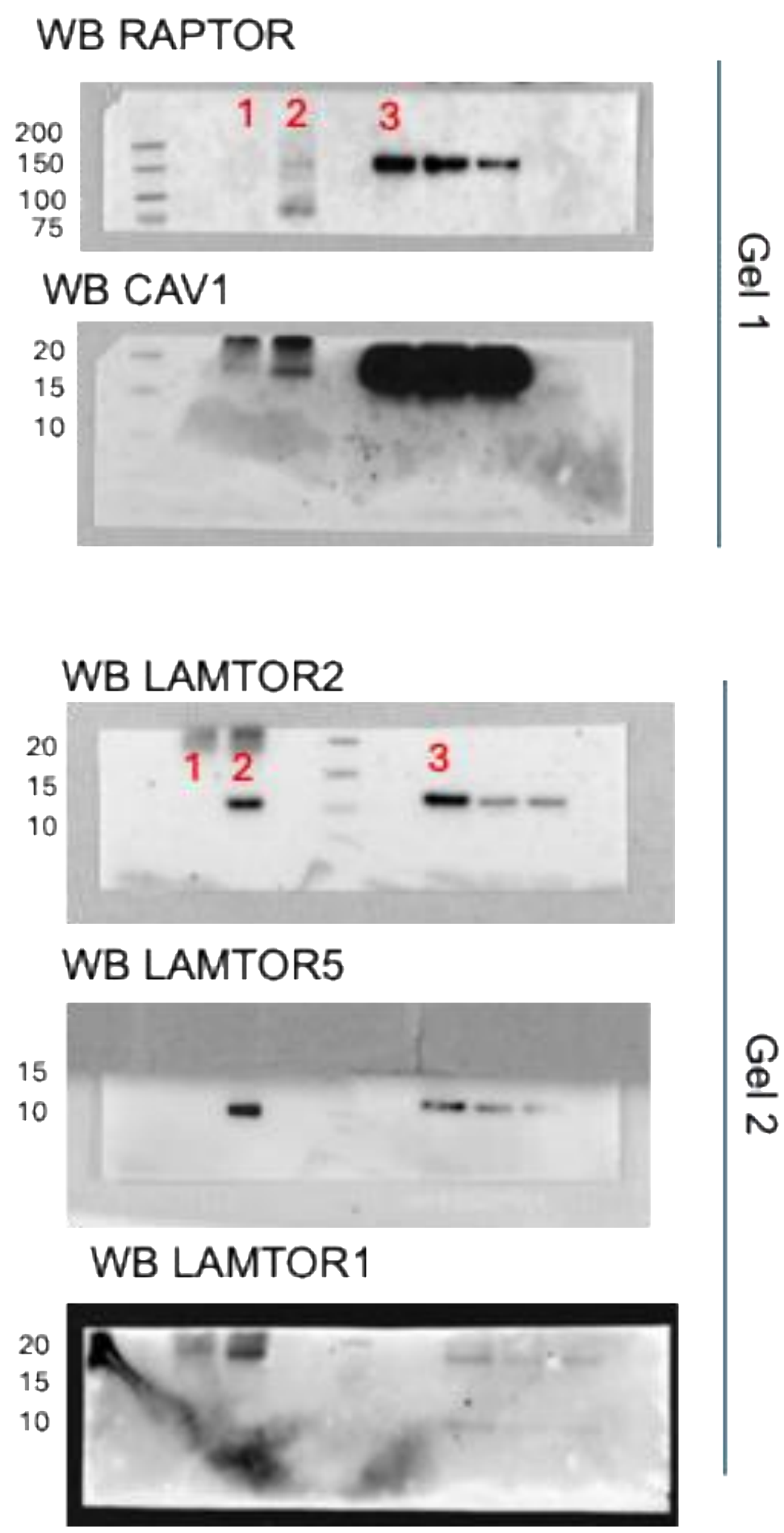

1 IP Ig Rabbit / XG21
2 IP LAMTOR1 / XG21
3 input XG21

Fig S7B

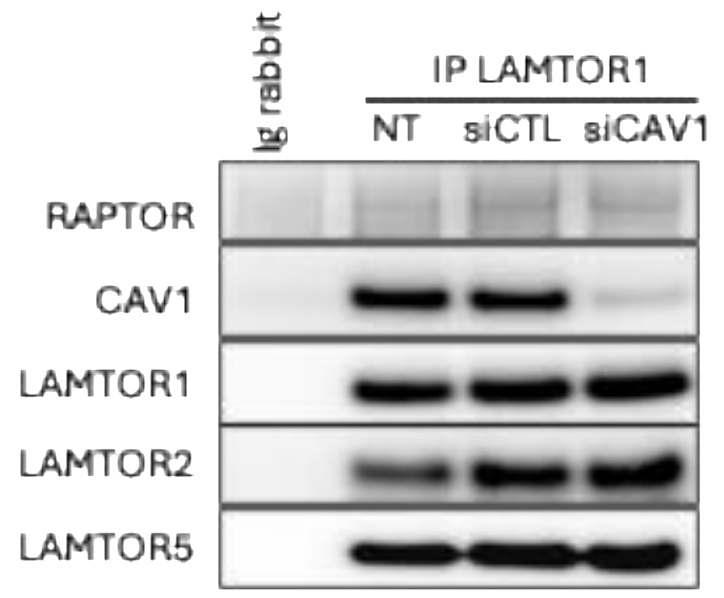

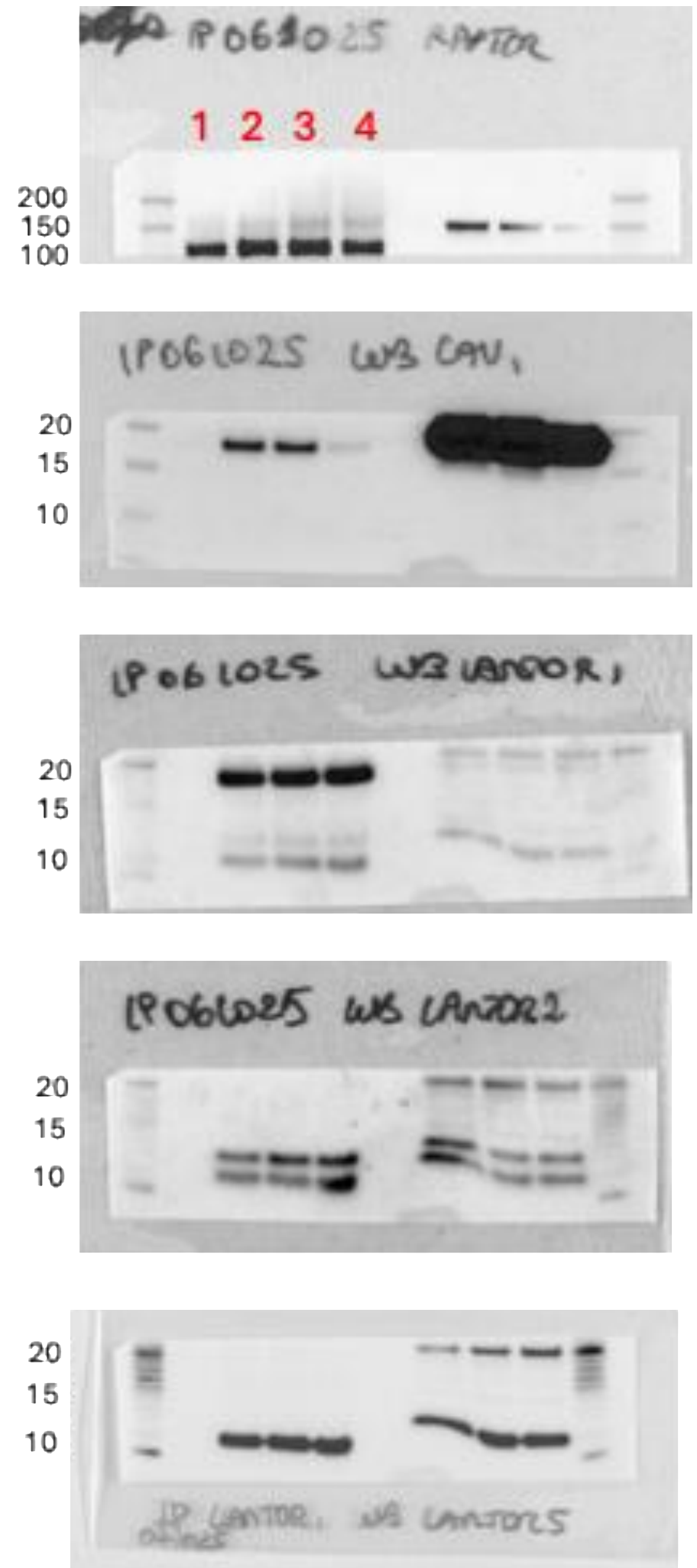

1 IP Ig Rabbit / non treated XG21
2 IP LAMTOR1 / non treated XG21
3 IP LAMTOR1 / siCTL treated XG21
4 IP LAMTOR1 / siCAV1 treated XG21

Fig S7C

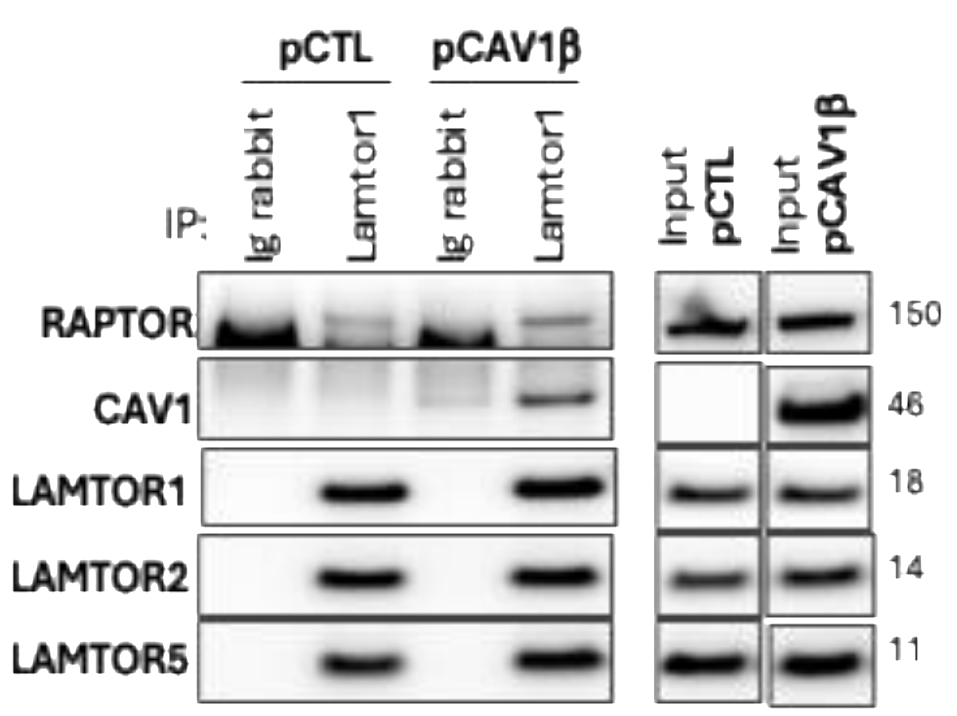

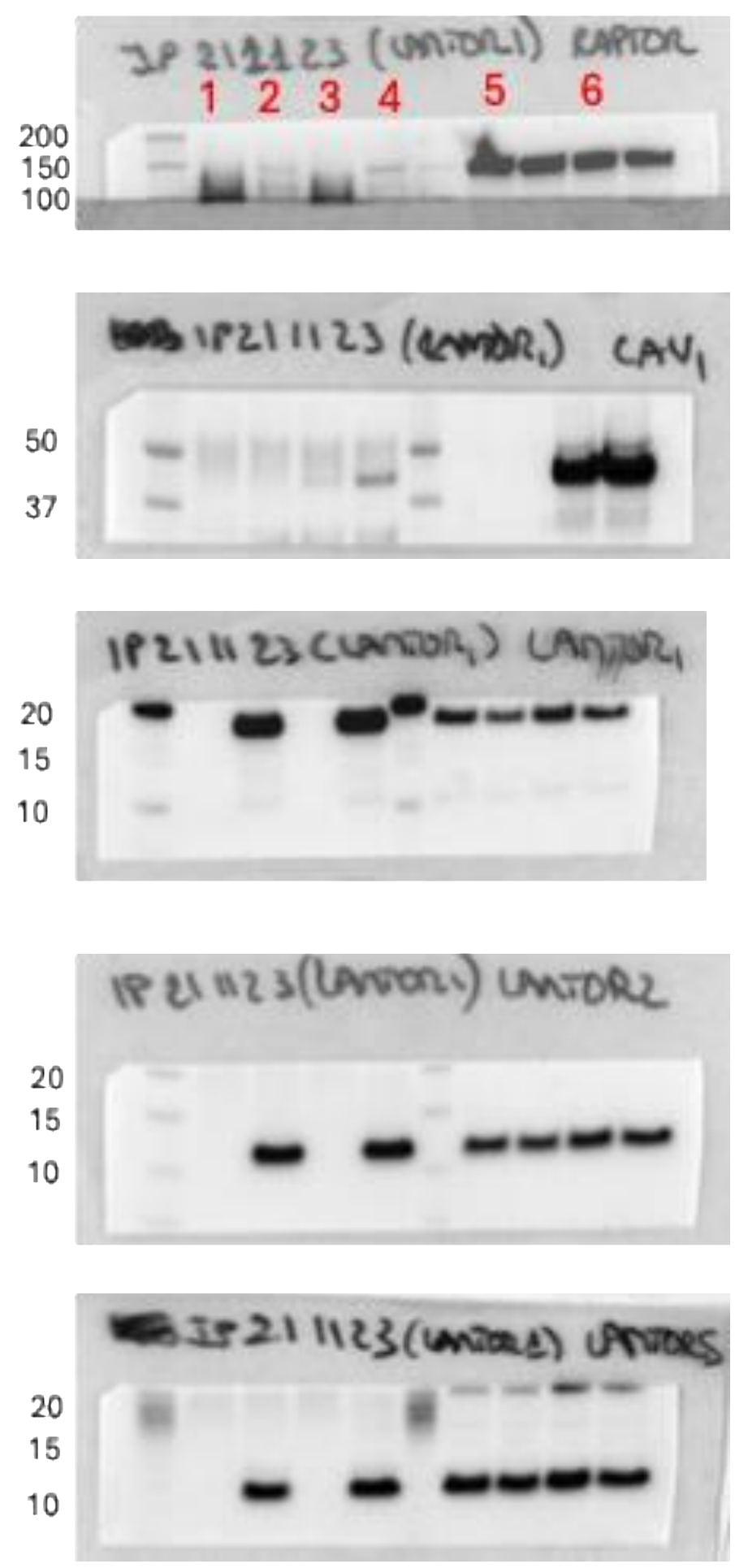

1 IP Ig Rabbit / DG75 plasmid control
2 IP LAMTOR1 / DG75 plasmid control
3 IP Ig Rabbit / DG75 plasmid CAV1b
4 IP LAMTOR1 / DG75 plasmid CAV1b
5 input DG75 plasmid control
6 input DG75 plasmid CAV1b
